# Minimalistic mycoplasmas harbor different functional toxin-antitoxin systems

**DOI:** 10.1371/journal.pgen.1009365

**Published:** 2021-10-21

**Authors:** Virginia Hill, Hatice Akarsu, Rubén Sánchez Barbarroja, Valentina L. Cippà, Peter Kuhnert, Martin Heller, Laurent Falquet, Manfred Heller, Michael H. Stoffel, Fabien Labroussaa, Joerg Jores

**Affiliations:** 1 Institute of Veterinary Bacteriology, University of Bern, Bern, Switzerland; 2 Graduate School for Biomedical Science, University of Bern, Bern, Switzerland; 3 Friedrich-Loeffler-Institute—Federal Research Institute for Animal Health, Jena, Germany; 4 Biochemistry Unit, University of Fribourg and Swiss Institute of Bioinformatics, Fribourg, Switzerland; 5 Proteomics and Mass Spectrometry Core Facility, Department for BioMedical Research (DBMR), University of Bern, Bern, Switzerland; 6 Division of Veterinary Anatomy, Department of Clinical Research and Veterinary Public Health, University of Bern, Bern, Switzerland; University of Gottingen, GERMANY

## Abstract

Mycoplasmas are minute bacteria controlled by very small genomes ranging from 0.6 to 1.4 Mbp. They encompass several important medical and veterinary pathogens that are often associated with a wide range of chronic diseases. The long persistence of mycoplasma cells in their hosts can exacerbate the spread of antimicrobial resistance observed for many species. However, the nature of the virulence factors driving this phenomenon in mycoplasmas is still unclear. Toxin-antitoxin systems (TA systems) are genetic elements widespread in many bacteria that were historically associated with bacterial persistence. Their presence on mycoplasma genomes has never been carefully assessed, especially for pathogenic species. Here we investigated three candidate TA systems in *M*. *mycoides* subsp. *capri* encoding a (i) novel AAA-ATPase/subtilisin-like serine protease module, (ii) a putative AbiEii/AbiEi pair and (iii) a putative Fic/RelB pair. We sequence analyzed fourteen genomes of *M*. *mycoides* subsp. *capri* and confirmed the presence of at least one TA module in each of them. Interestingly, horizontal gene transfer signatures were also found in several genomic loci containing TA systems for several mycoplasma species. Transcriptomic and proteomic data confirmed differential expression profiles of these TA systems during mycoplasma growth *in vitro*. While the use of heterologous expression systems based on *E*. *coli* and *B*. *subtilis* showed clear limitations, the functionality and neutralization capacities of all three candidate TA systems were successfully confirmed using *M*. *capricolum* subsp. *capricolum* as a host. Additionally, *M*. *capricolum* subsp. *capricolum* was used to confirm the presence of functional TA system homologs in mycoplasmas of the Hominis and Pneumoniae phylogenetic groups. Finally, we showed that several of these *M*. *mycoides* subsp. *capri* toxins tested in this study, and particularly the subtilisin-like serine protease, could be used to establish a kill switch in mycoplasmas for industrial applications.

## Author summary

Mycoplasmas belong to a class of cell-wall deficient bacteria characterized by minimal genomes acquired through regressive evolution. Historically, they were thought to lack many of the common bacterial virulence traits including classical exotoxins and toxin-antitoxin (TA) systems. In this work, we confirmed the presence of different functional TA systems in several isolates of the caprine pathogen *Mycoplasma mycoides* subsp. *capri*. Our data also indicate that TA systems are widespread in other mycoplasma species of veterinary importance. This work paves the way for the investigation of the biological role of TA systems during mycoplasma chronic infections as they are likely to contribute to the parasitic lifestyle of mycoplasmas, persistence in their hosts as well as the buildup of antimicrobial resistance, as recently observed. The availability of synthetic genomics tools to modify a range of *Mycoplasma* pathogens and well-established challenge models will foster future research and shed the light on the importance of TA systems in mycoplasmas.

## Introduction

Mycoplasmas are minute bacteria that evolved through regressive evolution from a Gram-positive ancestor with low G+C content related to the genus *Clostridia* [1]. This extensive loss of genes affected many metabolic pathways including the biosynthesis of the peptidoglycan cell wall and even their capacity to synthesize essential cellular building blocks and nutrients, which make them strictly dependent on their hosts [2]. As a consequence, mycoplasma genomes were historically considered as degenerated and streamlined genomes, mainly composed of essential genes. The recent construction of the minimal synthetic mycoplasma cell (Syn3.0) [3], based on the genome of the caprine pathogen *M*. *mycoides* subsp. *capri* (*Mmc*) GM12 [4], contradicted this paradigm. Indeed, the Syn3.0 genome is composed of only 473 genes but 80 of them (17% of its genome) still encode proteins of unknown function [5], confirming that mycoplasma genomes still carry many genetic features yet to be unraveled.

The genus *Mycoplasma* encompasses important human pathogens such as *M*. *genitalium* [6] and *M*. *pneumoniae* [7] as well as important veterinary pathogens such as *M*. *mycoides* subsp. *mycoides* [8], *M*. *capricolum* subsp. *capripneumoniae* (*Mccp*) [9], *M*. *gallisepticum* [10], *M*. *bovis* [11] and *M*. *hyopneumoniae* [12]. Although few mycoplasmas such as *Mccp* can cause acute diseases with a relatively short incubation time and high lethality [13], many mycoplasma infections result in a rather chronic form of disease associated with a long persistence of the causative agent [2,14]. Bacterial persistence is generally associated with a state of growth arrest resulting in non-dividing cells that can survive environmental stresses such as temperature, pH, nutrient starvation [15]. This phenomenon can also be triggered in response to external factors such as phage infections or antibiotic exposure [16]. In the absence of efficient vaccines for many mycoplasma infections, antibiotic treatment is the only viable option to treat the latter. The continuous rise of antibiotic resistance observed for several mycoplasma species [17] could be the result of an increased selection of persister cells as it was recently shown for several natural and laboratory strains of *E*. *coli* [18].

Toxin-antitoxin (TA) systems are involved in cell growth arrest and bacterial persistence in several bacteria including pathogens of the gastrointestinal, urogenital and respiratory tract such as *Clostridia*, *Enterobacteriaceae* and *Mycobacteria*, respectively [15]. Additionally, they were often associated with a reduction of the cellular metabolism and/or the build-up of biofilms [19]. Originally discovered as addiction modules involved in plasmid maintenance [20], TA systems were since linked to many additional cellular processes such as DNA replication, translation, or cell division among others. Currently, TA systems are grouped into six different types based on the on the molecular mechanism resulting in toxin inhibition [21]. All types have in common that the toxin consists of a stable protein usually encoded in an operon together with a less stable cognate antitoxin, which counteracts the toxin activity. Type I and type III systems have a noncoding small RNA-type antitoxin that neutralizes the toxin via base pairing with the mRNA of the toxin or via direct binding to the toxin, respectively. Type II systems, the most common TA systems reported so far, have a proteinaceous antitoxin that binds and thereby inactivates the toxin. In type IV systems the antitoxin and toxin compete for the same binding partner instead of interacting with each other, while in type V systems the antitoxin is an RNase that degrades the mRNA of the toxin. Type VI systems have a proteolytic adaptor antitoxin that promotes the degradation of the toxin. Most recently, a type VII has been proposed, which is characterized by an antitoxin that renders the toxin inactive via post-translational modification [22].

In a recent work, the presence of a candidate TA system in the strain Syn2.0, a minimized mycoplasma cell, controlled by a synthetic genome derived of strain GM12, has been identified using global transposon mutagenesis [23]. This system consists of a gene pair annotated as an AAA-ATPase (JCVISYN2_132) and a hypothetical protein carrying a subtilisin-like serine protease domain (JCVISYN2_133), considered as the putative antitoxin and toxin, respectively. Mycoplasma genomes carrying deletions of both the gene pair as well as the putative toxin JCVISYN2_133 resulted in viable cells, while the sole deletion of the antitoxin JCVISYN2_132 was shown to be lethal [23]. So far, functional TA systems have not been proposed in other mycoplasmas, especially not in pathogenic field strains, but *in silico* searches using the TASmania database identified candidate TA systems in different mycoplasmas based on sequence homology searches [24].

In this study, we investigated further the presence, distribution, and functionality of TA systems in mycoplasmas. First, we selected three candidate TA systems in *Mmc* GM12, including two TA systems identified *in silico* in addition to one candidate TA system reported recently in the Syn2.0 cell and described above. The overall distribution of these TA systems in *Mmc* was first assessed by performing whole genome sequencing on fourteen strains and subsequent mapping of TA system homologues. Next, we performed proteomic and transcriptomic analyses in *Mmc* GM12 to assess their expression profiles in different conditions, including heat stress. We subsequently used toxicity neutralization assays to assess the functionality of each TA module using different heterologous expression systems, including *E*. *coli*, *B*. *subtilis* and *Mycoplasma capricolum* (*Mcap*). Thereafter, we extended the search for homologous TA systems to all *Mollicutes* and successfully confirmed and tested the functionality of one system in *M*. *feriruminatoris*, *M*. *bovis* and *M*. *gallisepticum*. Finally, we used the previously identified toxins in a programmed cell death using an inducible expression system in mycoplasmas.

## Results

### *In silico* identification and selection of candidate TA systems

TASmania [24], which is a discovery-oriented database, was used to identify candidate TA systems in the genome of *Mmc* GM12. Our *in silico* search revealed 38 genes encoding candidate toxins or antitoxins but, despite high confidence scores, many of them were found as orphans (**S1 Table**). Two of them, namely TA_160/1_ and TA_752/3_ were identified as putative TA systems. Gene descriptions in the TASmania database referred to the TA_160/1_ partners as abortive infection proteins, AbiEii (T_160_) and AbiEi (A_161_), which belong to a well-described protein family (Abi) reported to act as type IV toxin-antitoxin systems in *e*.*g*. *Streptococcus agalactiae* [25] (**Table 1**). The T_752_-encoding gene was denominated as cell filamentation protein (Fic) whereas the one coding for the A_753_ protein was described as DNA-damage-inducible protein J presenting similarities with the *E*. *coli* RelB family [26]. In addition, we also selected a third candidate TA system, namely TA_132/3_, previously suggested to encode a type II AAA-ATPase/subtilisin-like serine protease TA module in the semi-minimal mycoplasma cell JCVI-syn2.0 [23] to further investigate its functionality.

**Table 1:** Candidate toxins and antitoxins of *M*. *mycoides* subsp. *capri* GM12 investigated in this study. The top two candidate TA systems were identified *in silico* by using the TASmania database.

Name	Mnmemonic/ designation in manuscript	Size [nt/aa]	Gene annotation from Ensemble	Pfam/ predicted TA type	E-value in TASmania
TA_160/1_	MMCAP2_0160/ T_160_	753 /250	Abortive infection protein	AbiEii / IV	1.2e-53
	MMCAP2_0161/ A_161_	597 /198	Abortive infection protein	AbiEi / IV	3.8e-49
TA_752/3_	MMCAP2_0752/ T_752_	573 /190	Cell filamentation protein	Fic / II	N/A
	MMCAP2_0753/ A_753_	273 /90	DNA-damage-inducible protein J	RelB / II	3.5e-9
TA_132/3_	MMCAP2_0133/ T_133_	2274 /757	Subtilisin-like serine protease	peptidase_S8 / II	N/A
	MMCAP2_0132/ A_132_	1062 /353	AAA-ATPase	AAA / II	N/A

TA system: toxin-antitoxin system, T: toxin, A: antitoxin, N/A not analyzed

### Genomic organization of TA systems

The close vicinity of the genes encoding each partner of the three TA elements suggested that they can be structured in operons, which is a common feature of many TA systems. The presence of antitoxin-encoding genes upstream of their toxin counterparts, correlated with the absence of intergenic regions in between the two partners, confirmed that hypothesis for both TA_132/3_ and TA_160/1_. In contrast, the TA_752/3_ system presented a different genomic organization with the gene coding for the toxin upstream of its antitoxin partner, spaced by an intergenic region of 207 bp. An analysis of this intergenic region using BPROM identified one possible promotor, including putative -10 and -35 boxes, indicating that the antitoxin may have its own promotor.

To confirm these preliminary findings, we first tested the presence of transcripts covering the genes encoding the three candidate TA systems using *Mmc* GM12 RNA preparations **(S1 Fig)**. Transcripts of all three TA systems were successfully detected via reverse-transcription PCR performed on complementary DNA (cDNA) using primers targeting the genes encoding the candidate toxins and antitoxins (**S1A Fig**) but also using primers spanning an overlapping region between the two partners (**S1B Fig)**. Fainter amplifications were observed for the T_752_ and TA_752/3_ transcripts_._ Primer specificity was confirmed when used to amplify the corresponding regions on *Mmc* GM12 genomic DNA **(S1C and S1D Fig).**

To conclude on this, RNA-seq data were successfully generated and deposited in the public domain (NCBI project number PRJNA765891). Based on these data, we used two independent softwares, namely RockHopper and ANNOgesic, to predict operon structures. Both tools had very similar results and predicted 188 operons and 186 operons, respectively. Of the three candidate TA systems described here, only the TA_132/3_ pair was confirmed as an operon.

### PacBio sequencing, genome assembly, annotation and genome organization of *Mmc* strains

To further investigate the distribution of the candidate TA systems in the subspecies *Mmc*, we sequenced and analyzed fourteen additional strains showing a wide diversity of origins **(Table 2)**. We obtained between 67,246 and 203,646 PacBio long reads per genome sequenced, with at least 231x coverage **(S2 Table)**. Each of the genomes was assembled into one circularized chromosome and the key features associated to each of them are displayed in the **Table 2**. The size of the 14 genomes ranged from 1,019,889 to 1,172,610 bp, with a G+C content of 23.7%. All genomes contained 30 tRNAs, two ribosomal RNA operons and 1 tmRNA and encoded between 789 and 915 proteins coding sequences. The functional annotation, based on a BLASTP analysis of the coding sequences against UniProtKB, revealed an average of 122 hypothetical proteins (~15% of the total annotated CDS) for each genome. We also observed a high level of synteny between all the genomes and only the strains 152/93 and PG3 carried a large inversion of more than 100 kbp, which was confirmed by PCR amplifications **(S2 Fig)**. Interestingly, the strain 152/93 contained a plasmid (GenBank accession number CP068011) with a size of 1,875 bp, which was highly similar to the previously described pKMK1 [27] and p*Mmc*-95010 plasmids [28].

**Table 2:** *M*. *mycoides* subsp. *capri* strains used in this study.

Strain name	Year of isolation	Country of isolation	Host	No. of protein coding genes	Genome Size (bp)	G+C content (%)	GenBank accession number
152/93	1993	Canary islands	Goat	816	1,044,454	23.7	CP068010
171/93	1993	Spain	Goat	801	1,030,242	23.7	CP065586
7730	1994	France	Goat	865	1,114,563	23.8	CP065584
80/93	1994	Spain	Goat	796	1,019,889	23.7	CP065583
83/93	1993	Spain	Goat	789	1,026,354	23.7	CP065582
95010	1995	France	Goat	907	1,153,998	23.8	FQ377874
G1283.94	1994	Germany	Barbary sheep	816	1,049,305	23.7	CP065580
G1313.94	1994	Germany	Barbary sheep	814	1,048,951	23.7	CP065579
GM12	1979	USA	Goat	839	1,089,202	23.9	CP001621
IVB-X	unknown	unknown	unknown	853	1,084,275	23.7	CP065578
M-18	1988	Croatia	Goat	915	1,165,233	23.8	CP065577
M-5	1988	Croatia	Goat	849	1,097,914	23.9	CP065588
My-325	1986	Croatia	Goat	900	1,159,653	23.8	CP065576
My-I	1986	Croatia	Goat	879	1,172,610	23.8	CP065575
PG3	1950	Turkey	Goat	789	1,035,494	23.7	CP065581
Wi8079	2009	Germany	Goat	796	1,034,934	23.7	CP065574

### Mapping of the three candidate TA systems in the *Mmc*

**Fig 1:**
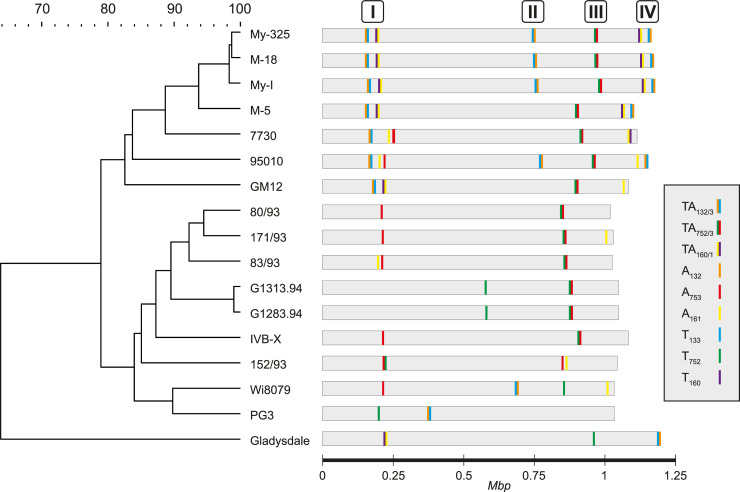
Cartoon displaying the genomic localization of candidate TA systems of *M*. *mycoides* subsp. *capri* GM12 in different *M*. *mycoides* subsp. *capri* strains and in one *M*. *mycoides* subsp. *mycoides* strain. Tree displaying the phylogenetic relationship of the strains based on whole genome sequence data is presented (left). *M*. *mycoides* subsp. *mycoides* strain Gladysdale was used as an outgroup. The tree was constructed in Bionumerics 8.0 using the comparative genomics tool applying standard parameters. The genomic locations of the different copies of the three candidate TA systems investigated in this study are depicted with vertical lines of different colors as detailed in the figure legend (right). The four main genomic regions harboring candidates TA modules are indicated as TA regions I to IV.

These newly sequenced *Mmc* strains, as well as the genomes of the two well-characterized strains GM12 and 95010 available in GenBank, were used to map the locations of all the homologs of the three putative TA systems (**Fig 1**). We constructed a phylogenetic tree of all 16 *Mmc* strains using whole genome data and carried out a TBLASTN analysis to identify the TA homologs (**Fig 1 and S1 File**). At first, the candidate TA modules were found unevenly distributed throughout the genomes but, strikingly, they appeared to cluster into four genomic regions, arbitrarily named “TA regions I to IV” **(Fig 1)**. The phylogenetic tree split in two main branches, one containing the GM12 strain (referred as ‘GM12 group’) and one harboring the PG3 strain (referred as the ´PG3 group’) and the distribution of each of the three candidate TA systems was found extremely divergent between the two groups. For instance, both the TA_132/3_ and TA_160/1_ were found in multiple copies in the ‘GM12 group’ but, besides the presence of few orphans, both were completely missing in the ‘PG3 group’. Three copies of the TA_132/3_ module were identified in the TA regions I, II and IV of the My-325, M-18, My-1 and 95010 while the other strains of the ‘GM12 group’ harbored only 1 or 2 copies. Except for 95010, most of the strains of the ‘GM12 group’ harbored 1 or 2 copies of the TA_160/1_ system located in the TA regions I and IV. Lastly, the TA_752/3_ pair was found as a single copy in all strains of the ‘GM12 group’, as well as more than half of the strains included in the ‘PG3 group’, always located in the TA region III. Only the strains 152/93, Wi8079 and PG3 did not harbor the full TA_752/3_ module but orphans (T_752_ or A_753_) were detected. Finally, the presence of the TA_132/3_ and TA_160/1_ systems in the phylogenetically closely-related *M*. *mycoides* subsp. *mycoides* strain Gladysdale, used as an outgroup, gave some indications that other mycoplasma species might also harbor TA modules in their genomes.

### Genomic context of the three candidate TA systems in *Mm*c

**Fig 2:**
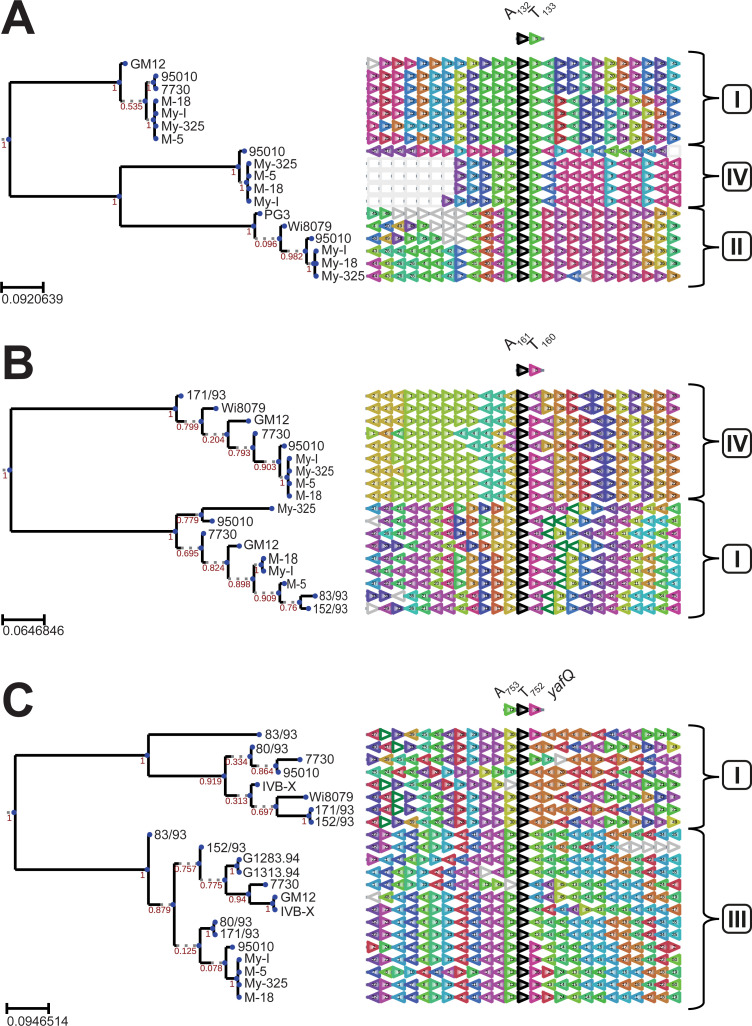
Genomic neighborhood and phylogenetic analysis of the candidate TA systems in *Mycoplasma mycoides subsp*. *capri*. The software FlaGs was used to infer the conservation of the genes flanking each candidate TA system, using each antitoxin as query (black arrowheads) and default parameters. Phylogenetic analyses of the different genomic neighborhoods are centered on each candidate antitoxin and are displayed for (A) the TA_132/3_ system, (B) the TA_160/1_ system, and (C) the TA_752/3_ system. Genomic loci are colored and numbered according to their homology (see S3 File for the identity of clusters with flanking gene accession numbers). Nonconserved genes are not colored. Local support values are estimated with FastTree (default settings) included in FlaGs. Numbers (I-IV) refer to the four main TA regions previously identified on *Mmc* genomes.

We investigated the genomic context in which the TA systems and their copies are located in the *Mmc* strains studied. For this purpose, the antitoxin gene was used as query in the software FlaGs to define flanking genes, which were then used to build a phylogenetic tree (**Fig 2 and S2 File**). The tree on one hand highlighted the sequence similarities between homologs across the different strains indicated by matching flag colors and numbers. On the other hand, it showed the variation of genetic context across different copies/paralogs of a given TA system within genomes. Thereby, three clusters were observed for TA_132/3_ (**Fig 2A**) which reflect the three different TA regions (I, II and IV) previously identified for on the *Mmc* genomes **(Fig 1)**. First, a close look at the TA region I allowed us to identify the presence of two direct repeats **(S2A Fig)**. Additionally, IS elements indicated by the flags 7 and 9 are located in close vicinity of TA_132/3_ in the TA regions II and IV (**S2B and S2C Fig**). Multiple tandem copies of flag 1 are present in the TA region IV. These genes are part of a genomic loci encoding several proteins annotated as hypothetical proteins but they share high similarities with several variable surface proteins (Vsps) identified on the well-annotated GM12 genome. Another specific observation are the alternating multiple copies of flags 2 and 3 in TA region II. An annotation analysis of these flags showed that they corresponded to candidate virulence factors, more specifically to the MIB-MIP pairs [29]. The TA_132/3_ copy of *Mmc* 95010 in TA region IV shows a unique genomic context compared to all others indicating a weaker synteny compared to the rest of its group in the corresponding tree.

The genomic context of the AbiEii/AbiGi-like TA_160/1_ was analyzed using again the antitoxin gene as query in FlaGs (**Fig 2B**). The inferred tree highlights the two genomic copies in TA regions I and IV as distinct paralogs. Several orphans of this A_160_ can be found (**Fig 1**). Interestingly, the flag 1 was present in multiple copies which showed similarities with variable membrane proteins of the *vpmA* locus previously reported in *M*. *agalactiae* [30] but also in other closely-related bacteria, the phytoplasmas [31] **(Figs 2B and S3D and S3E).**

Last, a closer look into the gene neighborhood of TA_752/3_ system (**Fig 2C**) showed the plasticity and complexity of the loci encoding this TA system. The antitoxin encoding gene A_753_ used as query appeared mostly as orphan in TA region I (**Fig 1**) and the different orthologs across the strains cluster together in the tree. Like all the other strains *Mmc* 83/93 did not show a T_752_ toxin but only the orphan A_753_ in TA region I. However, A_753_ in this strain was neighbored downstream by a gene annotated as *yafQ* toxin (flag 36). Interestingly, we detected the gene *yafQ* downstream of the TA_752/3_ paralog located in TA region III in strains 95010, My-I, M-5, My-325 and M-18. The presence of several IS elements/transposases was also identified in the close vicinity of this TA system **(S3F and S3G Fig)**.

Taken together these gene-level results with the phylogenetic analysis at the genome level **(Fig 1)**, strongly pointed towards horizontal gene transfer to be involved in TA systems dispersal and showed the plasticity of the toxin and antitoxin pairings.

### Detection of proteins encoded by candidate TA systems using a proteomic approach

**Fig 3:**
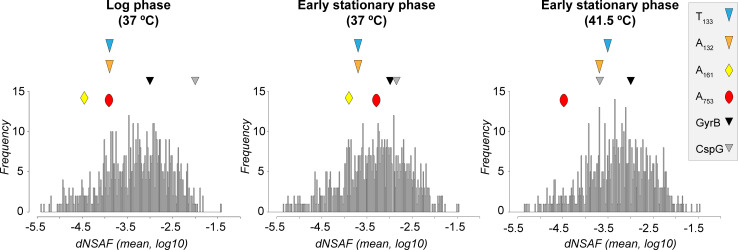
Detection of proteomic signatures of three TA systems investigated in *in vitro* grown *Mmc* GM12. The histogram shows relative protein abundance in strain GM12 [frequency over distributed normalized spectral abundance factor (dNSAF)], with candidate toxins (T) and antitoxins marked with different symbols. The analysis is based on three biological replicates. The protein abundance was tested in early log phase, early stationary phase and early stationary phase during heat stress using SP5 medium. Candidate toxins and antitoxins not displayed were below detection limits or absent. We included the house keeping proteins CspG and GyrB for comparison. The figure illustrates the dynamic changes of TA proteomic signatures over time, the TA_132/3_ had the strongest signatures and its antitoxin signatures was getting smaller compared to its toxin pair under heat stress.

We investigated the expression levels of the candidate toxins and antitoxins in *Mmc* GM12 using a shot gun proteomic approach. First, a culture of *Mmc* GM12, grown at 37°C, was harvested at mid-logarithmic phase as served as a reference. Two additional *Mmc* GM12 cultures were grown until early stationary phase at either 37°C and 41.5°C, the latter mimicking a heat stress similar to the fever episodes encountered in their natural hosts during infections. The expressions of the TA proteins in the three different conditions were analyzed and plotted using the mean distributed normalized spectral abundance factor (dNSAF) **(Fig 3)**. In order to compare between all three conditions, the expression of the house-keeping enzyme DNA gyrase subunit B (GyrB) and the cold shock protein (CspG) was used. When grown at 37°C until mid-log phase, they presented a log_10_ (mean dNSAF) value of -3.0 and -2.0, respectively. In comparison, the A_132_, A_753_ and A_161_ as well as T_133_ proteins were detectable in low abundance with log_10_ (mean dNSAF) values below -3.7. The other candidate toxins and antitoxins were below the detection limit of the mass spectrometry analysis (**Fig 3 and S3 File**). During the heat shock treatment, the house keeping control protein GyrB abundance remained constant, while the CspG protein’s abundance strongly decreased, as expected. The latter was also observed for the antitoxins A_161_ and A_753_ with the A_161_ even going below detection limits. The changes in the expression levels of the TA_132/3_ proteins was less obvious as the heat shock treatment resulted in a small shift in the toxin-antitoxin expression ratio with the toxin becoming slightly more abundant than its cognate antitoxin.

### Functionality testing of candidate TA systems using phylogenetically distant heterologous expression systems

**Fig 4:**
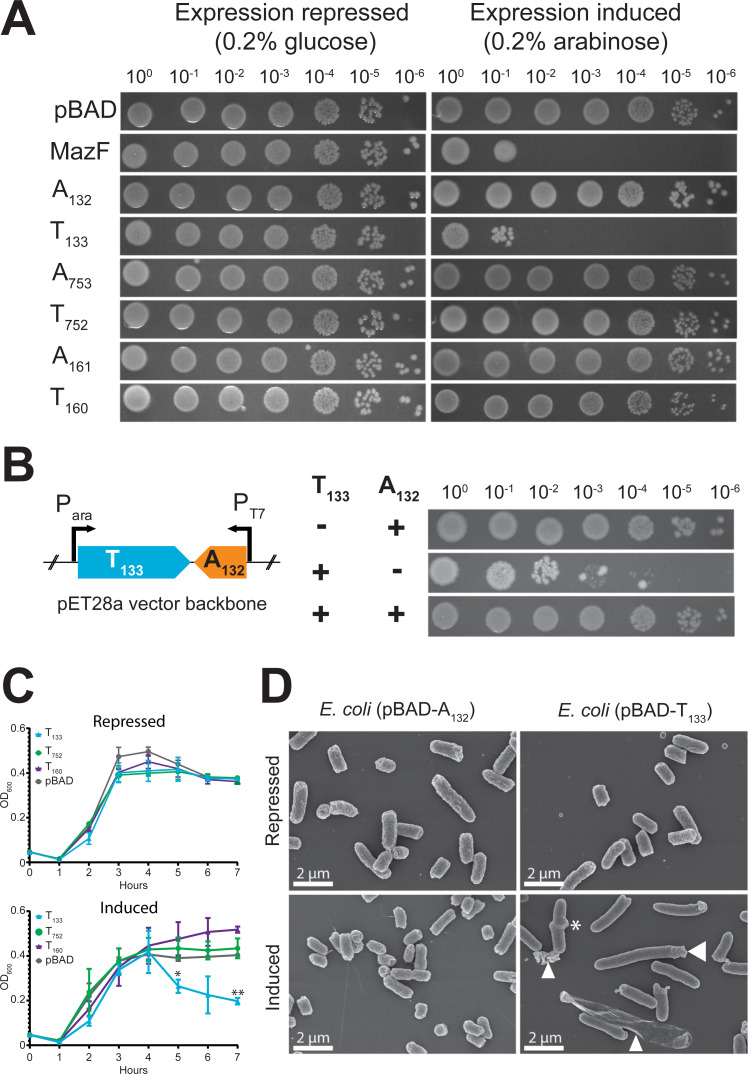
Toxicity of recombinant candidate TA systems in *E*. *coli*. (A) Spot assays of *E*. *coli* LMG194 in response to induction or repression of heterologous expression of the different toxins, antitoxins and entire TA systems. The empty vector pBAD/His was used as negative control and the toxin MazF was used as positive control. Each data point represents the mean of three biological replicates, bars indicate standard deviation. (B) Neutralization experiments employing a dual expression system based on the pET28a vector enabling the expression of the two TA partners individually or in combination. Schematic representation of the construct is displayed (left) while the spot assay results are presented (right). A toxic effect is only observed after induction of the toxin but neutralization of the toxin activity was obtained via concomitant expression of the antitoxin. (C) Growth curves of *E*. *coli* LMG194 in response to induction or repression of heterologous expression of T_133_, T_160_, T_752_ and empty vector pBAD/His. Each data point represents the mean of three biological replicates, bars indicate standard deviation. The p-values are displayed (* *p* ≤ 0.05, ** *p* ≤ 0.01).). (D) Scanning electron micrograph (magnification 10,000x) displaying morphological changes of *E*. *coli* LMG194 observed 7 hours after the induction of T_133_ and A_132_ recombinant proteins. Blebbing at the pole is indicated by an arrow, z-shaped cells by asterisk and cell debris by arrowheads.

First, codon-optimized synthetic genes encoding candidate mycoplasma toxins and antitoxins were individually cloned into *E*. *coli*. The codon optimization was necessary since the mycoplasmas use a genetic code that differs from the universal genetic code resulting in the introduction of premature opal codons (UGA) once introduced in *E*. *coli* [32]. Candidate encoding genes were inserted under the control of the arabinose-inducible *araBAD* promoter of the pBAD/His expression system. The *E*. *coli* endoribonuclease toxin MazF [33] and the empty pBAD/His vector were used as positive and negative controls, respectively. Expression of the recombinant proteins was either repressed using glucose or induced using L-arabinose and cultures were incubated at 37°C for 5 hours followed by spot dilutions onto plates using ten-fold dilutions up to 10^−6^ (**Fig 4A**). No growth difference was observed between the different clones when the *araBAD* promoter was repressed. Bacteria concentrations were all ranging between 1.5 x 10^8^ and 3 x 10^8^ cells/mL (**Fig 4A**, left panels). In contrast, a 5-log reduction in bacteria concentration was observed upon induction of the recombinant *E*. *coli* MazF toxin and the candidate *Mmc* T_133_ (**Fig 4A**, right panels). No toxic activity was recorded for the other *E*. *coli* clones upon induction of the remaining TA toxins and antitoxins. The expression of each candidate His6-tagged mycoplasma protein was confirmed by immunoblotting (**S4 Fig**). Variable expression levels were observed but all proteins were expressed at least three hours after induction. We further investigated the capacity of the antitoxin A_133_ to neutralize the toxic activity of the candidate T_132_. To do so, we built an *E*. *coli* dual expression system based on the pET28a expression system (**Fig 4B**). This system allows the individual or concomitant expression of the two proteins using different inducers (arabinose and IPTG). The functionality of this system was tested for the individual expression of each protein. As expected, we observed a 3-log reduction in bacteria concentration when the expression of the T_133_ protein was induced whereas no growth difference was observed upon induction of the A_132_ counterpart (**Fig 4B**). More importantly, a complete neutralization of the T_133_ toxic activity was obtained when the two proteins were expressed together (**Fig 4B**). Expression of recombinant proteins was confirmed. We then phenotypically characterized the toxic activity observed for the T_133_. Therefore, we followed the growth of the *E*. *coli* clone carrying the pBAD-T_133_ for seven hours under repressed (**Fig 4C**) or induced conditions (**Fig 4C**). Under repressed conditions, no growth defect was observed for this clone when compared to the other *E*. *coli* clones carrying the pBAD-T_160_ or the pBAD-T_752_. When arabinose was added, all three clones grew similarly until late exponential phase (4 hours post-induction). However, four hours post-induction, the OD_600_ of *E*. *coli* clone harboring the pBAD-T_133_ construct started to decrease drastically compared to the various controls (**Fig 4C**). Interestingly, the growth defect observed for this clone was also associated with a clear clumping of cells in the liquid culture. To better characterize this phenotypic observation, we visualized these *E*. *coli* cells at 7 hours post-induction using scanning electron microscopy (SEM) (**Fig 4D**). Micrographs revealed that the cells expressing the T_133_ were presenting several morphological alterations when compared to the cells expressing the A_132_ or those where the T_133_ expression was repressed. Cells were abnormally elongated and showed atypical z-shape morphologies (**Fig 4D**, indicated by an asterisk). Obvious blebbing was also observed at one cell pole indicating early signs of cell death (**Fig 4D**). Lastly, burst *E*. *coli* cells were present as well as cellular debris (**Fig 4D**).

Altogether, these results confirmed that one out of the three candidate *Mmc* TA systems, namely the TA_132/3_ system, was functional in *E*. *coli*. Despite lacking a cell wall, mycoplasmas are closely related to the gram-positive bacteria. Therefore, we wanted to use the closely related *Bacillus subtilis* to assess the functionality of the three candidate *Mmc* TA modules, in particular for those that could not be previously characterized.

The expression of the candidate TA partners, either individually or in combination, was attempted using the commercially available pHT01-based expression system transformed in *B*. *subtilis* strain 168. The monitoring of the optical density of individual TA elements showed only a lag in growth for clones harboring pHT01-T_752_ and pHT01-A_132_ (**S4A Fig**). This phenotype was confirmed by the spot dilution assay (**S4B Fig**). Next, we tested the expression levels in *B*. *subtilis*, to confirm expression and to correlate expression with a growth phenotype. Despite several attempts, we failed to obtain detectable levels of expression for all candidates except for recombinant A_132_ and T_133_ proteins (**S4C Fig**). Even though expression of the T_752_ protein was not visible in a Coomassie-stained gel, SEM micrographs showed an altered cell morphology compared to cells harboring the empty vector (**S4D Fig**). A large fraction of cells harboring T_752_ burst, shrunk or presented holes at their surface. In contrast we did not observe a phenotype related to cell death in the clones harboring A_132_.

### Functional characterization of candidate *Mmc* TA systems using *M*. *capricolum*

**Fig 5:**
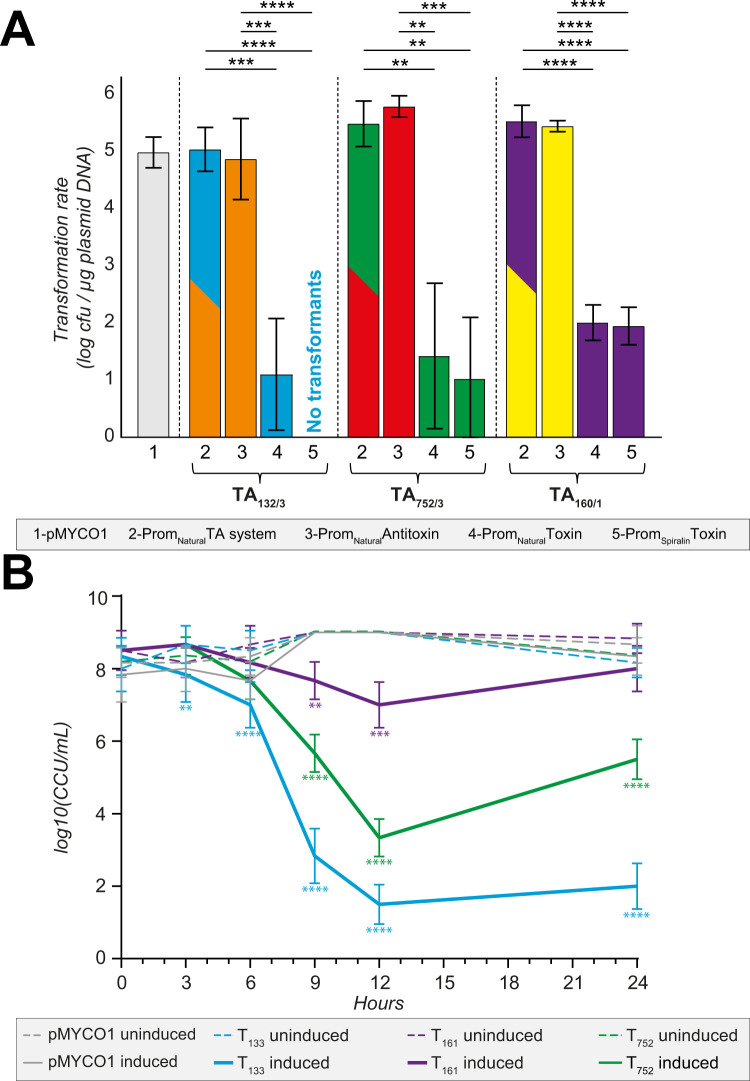
Effect of cloned toxins, antitoxins and TA systems on the transformation rate into *M*. *capricolum* subsp. *capricolum*. (A) *M*. *capricolum* subsp. *capricolum* ATCC 27343^T^ was transformed with different plasmid constructs harboring either entire TA operons (TA_132/3_, TA_752/3_ and TA_160/1_), individual toxins or antitoxins. Empty pMYCO1 plasmid was used as a positive control. Each column represents the mean of three independent biological replicates and bars indicate standard deviations. Significance is indicated (** *p* ≤ 0.01, *** *p* ≤ 0.001, **** *p* ≤ 0.0001). (B) *M*. *capricolum* subsp. *capricolum* ATCC 27343^T^ was transformed with different plasmid constructs harboring the toxins (T_133_, T_752_ and T_160_) under the control of an inducible promotor. Empty pMYCO1-Chlo^R^ plasmid was used as control. Graphs represents the mean of three independent biological replicates and bars indicate standard deviations. Significance is indicated (** *p* ≤ 0.01, *** *p* ≤ 0.001, **** *p* ≤ 0.0001).

In light of the issues encountered in using phylogenetically distant heterologous systems such as *E*. *coli* or *B*. *subtilis*, we opted to ultimately characterize all three candidate *Mmc* TA systems directly in mycoplasmas. We took advantage of the availability of the mycoplasma-based replicating plasmid pMYCO1 [34], as well as previously developed transformation protocols [35], to study the function of each TA partner in *Mcap*. We first constructed a set of four plasmids for each of the three candidate TA systems. These constructions included the gene(s) encoding (i) the entire TA module, (ii) the antitoxin only, (iii) the toxin only, all under the control of their natural promoter regions, as well as (iv) the toxin only under the control of the strong spiralin promotor [36]. Sequence-verified plasmids (**S1 File**) were transformed into *Mcap* and transformation efficacies were monitored to assess toxicity (**Fig 5**). Transformations with plasmids carrying the antitoxins yielded the same amount of transformants (~10^5^−10^6^ transformants per μg of plasmid) than those performed with the empty vector (**Fig 5A**). The transformation rates significantly dropped (3–3.5 logs difference) when plasmids harboring the toxins only were used. The number of transformants harboring the toxin with the natural promotor compared to the ones containing the spiralin promoter did not differ significantly, except when pMYC01-pSpi-T_133_ was transformed as no transformants were isolated. Interestingly, transformation rates obtained with pMYCO1 plasmids harboring both TA partners were identical to the empty control, conclusively showing the neutralization capacity of all the antitoxins (**Fig 5A**). A maximum of five transformants per construct and per experiment were selected, passaged and sequenced. No mutations were found in the sequences of the genes coding the antitoxins and entire TA systems. *Mcap* cells transformed with the toxin pMYCO1-pNat-T_133_ were found to not contain the toxin gene. About half of the transformants harboring the pMYC1-pNat-T_752_ were intact whereas none of the pMYCO1-pSpi-T_752_ ones contained the toxin genes anymore. No mutations were found in the nucleotide sequences of the constructs containing the toxin T_160_ (both versions).

### Induction of cell death in mycoplasmas

The identification of three functional *Mmc* toxins prompted us to test if such toxic proteins could be used to trigger cell death in mycoplasmas. We built additional plasmid constructs based on the only available inducible promoter developed for mycoplasmas, namely the tetracycline-inducible promoter TetR-Pxyl/tetO_2_ [37,38]. A chemically-synthetized sequence of this inducible promoter was introduced in the pMYCO1-Chlo^R^ where a chloramphenicol counterpart replaced the original tetracycline resistance cassette, thus enabling the selection of the transformants. Each of the three toxin-encoding genes was inserted under the control of the tetracycline-inducible promoter and plasmids were transformed in *Mcap* as previously described. Resulting clones were grown until late logarithmic phase then tetracycline (1 μg/mL) was added to induce the expression of the toxins. In parallel, the same *Mcap* clones were also grown in absence of tetracycline and were used as negative controls. Samples were taken at different time-points; ten-fold serially diluted up to 10^−9^ in non-selective SP5 medium and incubated at 37°C in 96-well plates. Color changing units (CCUs) were calculated as the last dilution where an acidification (from red to yellow) of the medium due to *Mcap* growth was recorded. The results of such experiments are displayed on Fig 5B. The induction of all three toxins resulted in a significant decrease of bacteria concentrations as early as 6 hours post-induction. The maximal toxic activity was recorded 12 hours post-inoculation. The toxin T_133_ was particularly effective in triggering cell death as *Mcap* concentrations dropped from 10^9^ CCU/mL at T0h to as low as 10^1^ CCU/mL at T12h (**Fig 5B**). The toxin T_752_ was also effective and 5–6 logs difference in bacterial concentrations were consistently observed at T12h. In the absence of tetracycline, all cultures were able to survive at least for 24 hours in stationary phase before entering the death phase.

### Functional TA systems are unequally distributed in mycoplasmas

**Fig 6:**
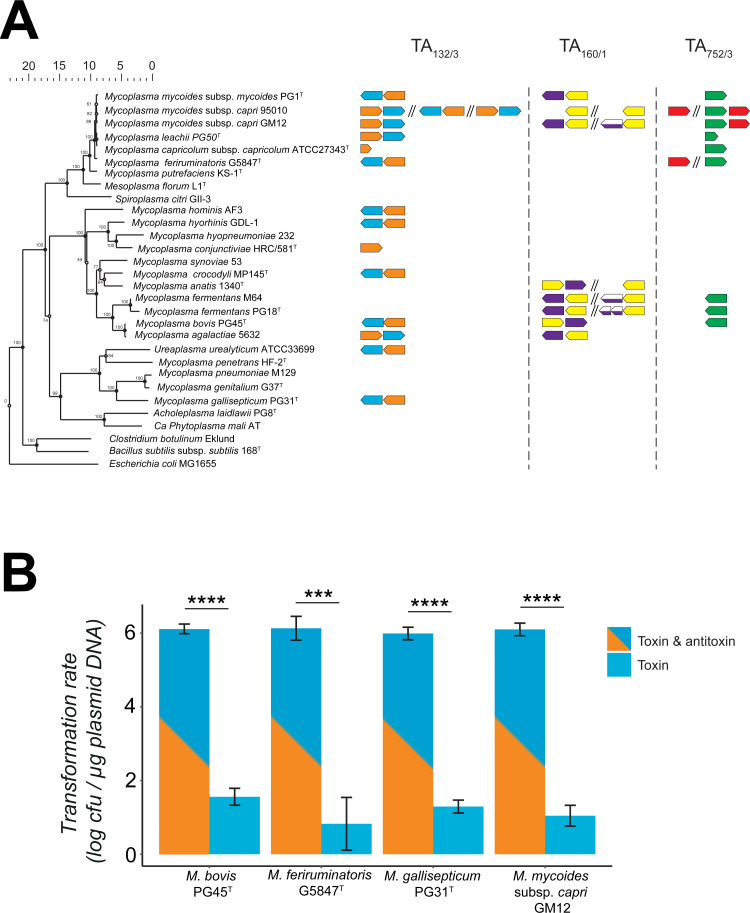
Detection of homologues of the three investigated TA systems in selected mycoplasmas and the functional analysis of homologues of the TA_132/3_ in *M*. *bovis*, *M*. *feriruminatoris*, and *M*. *gallisepticum*. (A) A tree highlighting the phylogenetic relationship of the different species based on 16SrRNA gene sequences is displayed (left). The outgroup species *Clostridium botulinum*, *Bacillus subtilis* and *Escherichia coli* were included. Tree was constructed using Bionumerics 8.0. Bootstrap values indicated at the nodes were calculated using 500 simulations. Identification of the *Mmc* TA system homologs (right) was done using Molligen 4.0. (B) *M*. *capricolum* subsp. *capricolum* ATCC 27343^T^ was transformed with different plasmid constructs based on pMYCO1 harboring the T_133_ and TA_132/3_ homologues of *M*. *bovis*, *M*. *feriruminatoris* and *M*. *gallisepticum* under the control of the natural promotor of *M*. *mycoides* subsp. *capri*. Control plasmids were pMYCO1 plasmid harboring T_133_ and TA_132/3_ from *M*. *mycoides subsp*. *capri*. Graphs represents the mean of three independent biological replicates and bars indicate standard deviations. Significance is indicated (*** *p* ≤ 0.001, **** *p* ≤ 0.0001).

We have previously shown that phylogenetically closely related strains within the same mycoplasma subspecies, namely *M*. *mycoides subsp*. *capri*, presented highly divergent genetic profiles when it came to the distribution and numbers of genetic loci encoding the three different TA systems (**Fig 1**). We wanted to expand on these findings and investigated the distribution of TA system homologs in other mycoplasma species, including many veterinary pathogens of importance (**Fig 6A**). We first exploited the genomic comparative tools present in the Molligen database to search for highly significant homologs in mycoplasma species belonging to the Spiroplasma, Hominis and Pneumoniae group spanning the entire class of *Mollicutes*. The results of our *in silico* analyses are presented in the Fig 6A and all the outputs used to generate it are included in the **S5 File**. Overall, homologs of the three *Mmc* TA partners were found in other mycoplasma species. The least conserved TA system was found to be the TA_752/3_. No complete TA module was found outside of the *Mmc* species but homologs of the T_752_ were observed in several mycoplasma species including three strains of *M*. *fermentans*, two strains of *M*. *bovis* and *Mcap* ATC27343^T^. Based on their amino acid conservation (~60% or lower), it is possible that these genes encode nonfunctional toxins or that other antitoxins can neutralize their activity *in trans*. Hits for the TA_160/1_ system were recorded, but were concentrated in a small phylogenetic cluster including *M*. *anatis*, *M*. *fermentans*, *M*. *bovis* and *M*. *agalactiae*. *M*. *mycoides* subsp. *mycoides* PG1^T^ was the only other member besides *Mmc* of the ‘*Mycoides* cluster’ where an entire TA system homolog was found. Additional copies of the A_161_ were detected for some of the previously cited mycoplasma species and it was also the case for *Mmc* strain 95010. The TA_132/3_ is by far the most widespread TA module of the three investigated. It is well conserved within the ‘*M*. *mycoides* cluster’ and it is present in all species except *Mcap*. Homologs for this system were found in all phylogenetic groups. These included *M*. *hominis*, *M*. *hyorhinis*, *M*. *bovis* and *M*. *agalactiae*, all representatives of the Hominis group, as well as two members of the Pneumoniae phylogenetic group, namely *M*. *gallisepticum* and *Ureaplasma urealyticum*. We then took full advantage of the absence of homologs for this TA system in *Mcap* to test if some of these candidate TA_132/3_ homologs were functional. We repeated the functional assay developed in *Mcap* and constructed six additional pMYCO1-based constructs harboring either the complete TA system or the toxin only for the homologs found in *M*. *feriruminatoris* G5847^T^ (Spiroplasma group), *M*. *bovis* PG45 (Hominis group) and *M*. *gallisepticum* PG31 (Pneumoniae group). All these constructs were transformed into *Mcap*ΔRE and transformations efficiencies were compared to those obtained with the original pMYCO1-TA_132/3_ and pMYCO1-T_133_ of *Mmc* GM12. Low transformation efficiencies (<10^2^ transformants/μg) were obtained for all constructs harboring the toxin alone (**Fig 6B**). As expected, much higher rates (~10^6^ transformants/μg) were obtained when both the toxin and antitoxins were present. This clearly shows that the homologs of the TA_132/3_ system identified in the three mycoplasma species are true TA systems and that they are functional in *Mcap*. It is therefore very likely that this also hold true for the other TA systems identified in *silico* in the other mycoplasma species.

## Discussion

Mycoplasmas are known to cause chronic diseases in both humans and animals [2]. Antimicrobial treatment options for mycoplasmas suffer from both increasing resistance [17] and a limited spectrum of antimicrobials available due to the absence of a cell wall. Live *Mycoplasma mycoides* subsp. *mycoides*, the causative agent of contagious bovine pleuropneumonia, have been isolated from lung sequestra more than 6 months after experimental infection and antibiotic treatment [14]. Similar observations were reported for human pathogenic species such as *M*. *genitalium* [39] and *M*. *pneumoniae* [40]. Altogether, these data pointing towards the ability of these mycoplasmas to persist under antimicrobial pressure. It is well accepted that TA systems contribute to the apparition of persister cells that favors the development of antimicrobial resistance. The presence of TA modules in mycoplasma genomes, as well as other classical toxins, has long been overlooked due to their small genome sizes. Recently, two candidate TA systems were identified *in silico* using the TASmania database including a type II TA system corresponding to a Pfam YafQ/RelB-like pair and a type IV system related to the AbiEii/AbiEi_4-like pair [24]. These putative TA systems were found in the genomes of *M*. *feriruminatoris* and *M*. *agalactiae*, respectively, but have not been investigated experimentally ever since. Our study first aimed to confirm the presence of functional TA systems in pathogenic mycoplasma and to investigate their distribution across the class of *Mollicutes*. Therefore, we started out with our model organism, the highly pathogenic *Mycoplasma mycoides* subsp. *capri* strain GM12. The *in silico* analysis of this strain using TASmania database revealed 18 chromosomally-encoded candidate TA modules. We selected two candidate TA pairs, namely the TA_160/1_ and TA_752/3_, which were predicted with high confidence as they were presenting homologues to known systems such as AbiEii/AbiEi and TA effectors such as Fic or RelB, respectively. The system AbiEii/AbiEi has been confirmed as functional in other bacteria, while the elements of the Fic/RelB pair found in *Mmc* have been shown to be functional in other TA pairs independently, but never as TA partners [25,26,41]. We decided to add the candidate TA_132/3_, which was proposed recently as a putative TA system [23] based on the analysis of a transposon insertion library of the synthetic cell JCVI-syn2.0. This novel TA module encodes a putative AAA-ATPase/subtilisin-like serine protease as antitoxin and toxin, respectively. First, we were able to detect transcripts of all TA partners using RT-PCR as well as confirming the genetic organization of the TA_132/3_ in an operon structure based on RNA-seq. Protein signatures for at least one partner of the three pairs have been obtained. In line with its prediction as a type II TA system, protein signatures were obtained for both TA_132/3_ partners. We simulated a heat stress by incubating *Mmc* GM12 cultures to a rapid but prolonged increase of temperature up to 41.5°C. Stress such as heat is known to induce a faster degradation of antitoxin, allowing a relative increase of the toxin cognate abundance, which is exactly what we have observed for the T_133_ toxin (**Fig 3**). Protein signatures for the putative AbiEii/AbiEi and Fic/RelB TA pairs were restricted to the antitoxins. The failure to detect the candidate toxins does not necessarily correlate with its absence and is by no means evidence of a nonfunctional TA pair. Small amounts of toxins are generally sufficient to fulfill its role and its concentration might be below the detection limit of the mass spectrometer analyzer.

The functionality of the three candidate TA systems was assessed using several heterologous systems. In our hands, the use of the two classical model organisms *E*. *coli* and *B*. *subtilis* for the study of mycoplasma TA systems resulted in rather inconclusive data. Using a toxicity neutralization assay in *E*. *coli*, we confirmed the AAA-ATPase/subtilisin-like protease as a functional TA module. We constructed a pET28-pBAD hybrid plasmid so that both the antitoxin and the toxin are under the control of a different inducible promoter (**Fig 4B**).

The T_133_ toxin belongs to the Peptidase_S8 family (Pfam PF00082), mainly consisting of endopeptidases. Subtilisin-like serine proteases are present in many organisms including bacteria, viruses but also eucaryotes [42]. Subtilases were mainly studied in plants for their implication in plant development or defense mechanisms against pathogens [43]. Recently, the subtilase IetS of the gram-negative plant pathogen *Agrobacterium tumefaciens* was reported as a toxin involved in a type II IetS/IetR TA system conferring plasmid stability [44]. The IetR counterpart also belong to an AAA-ATPase family as the A_132_ described for *Mmc*. Even if we have no evidence of its function in *Mmc*, it is very unlikely that the chromosomally-encoded TA_132/3_ of *Mmc* has a role in plasmid maintenance. Heterologous expression of T_133_ in *E*. *coli* resulted in cell clumping in liquid culture and elongated and filamented cells. The observed morphological changes can be attributed to either direct toxic effects or a stress response of *E*. *coli* towards the recombinant T_133_, which will be investigated in future studies. Cell filamentation has been reported to occur as an SOS response upon DNA damage in other bacteria [45,46]. Subsequently, we tested the TA systems using heterologous expression in *B*. *subtilis*, since this species is phylogenetically closer related to the mycoplasmas than *E*. *coli*. Again, the results obtained were rather inconclusive, confirming only toxic activity of the *Mmc* T_752_ Fic protein (**S5 Fig**). Fic proteins are known in bacteria to disrupt the DNA topology via adenylation leading to growth arrest [41]. Low expression levels of recombinant TA proteins or bacterial growth issues, as observed for the A_132_ (**S5 Fig**), did not allow us to draw conclusive results from these *Bacillus* experiments. It has to be mentioned that subtilases have also been described in *B*. *subtilis* [47] and might have interfered with our assay. Ectopic expression of TA pairs benefits from the absence of identical TA pairs to the ones to be tested. Conditional cooperativity [21] of TA elements cannot be excluded and might have contributed to the inconclusive data obtained from the *Bacillus* and *E*. *coli* experiments. Finally, we tested the three TA pairs in *M*. *capricolum* for transformation experiments, which are in our opinion most meaningful due to the close phylogenetic distance between *Mmc* and the recipient cell used for heterologous expression. Especially, in type IV systems, like the AbiEii/AbiEi pair investigated in our study, antitoxin and toxin are competing for the same bacterial target. If that target is absent, as it might have been the case in *B*. *subtilis* and *E*. *coli*, a functional proof of that type IV TA system is impossible. We were able to confirm functionality of all three systems investigated in *Mcap* and these experiments clearly showed the capacity of the antitoxins to neutralize the toxins functions (**Fig 5**). Moreover, we verified that plasmid instability was not biasing our results. We constructed plasmids harboring the candidate toxins under the control of a tetracycline-inducible promotor [37]. These results confirmed the overall plasmid stability when the toxin expression is inhibited and high toxicity triggering cell death after induction (**Fig 5B**). Especially the T_133_ subtilisin-like serine protease has been confirmed to be promising element for a kill switch in an engineered mycoplasma chassis, in case alternative inducible promotors are at hand. Recently, we engineered an *obg*-based temperature kill switch, that was activated during elevated temperatures and induced growth arrest [48]. In summary, the functional testing of mycoplasma candidate TA pairs was most informative when *Mcap* was used as host for ectopic expression. Despite recent advances using synthetic genomic tools, the functional studies of mycoplasma proteins still suffer from limited tools that did not allow us to further investigate the mode of action of any of the *Mmc* TA systems. The generation of isogenic mutants, deleted for the individual TA partners, might be of interest and will be explored in future studies. To get insight into the genomic localization of the *Mmc* TA pairs investigated, we sequence-analyzed 14 additional genomes of *M*. *mycoides* subsp. *capri*, reflecting the diversity of the subspecies [49]. We noticed that, in many strains, the TA pairs or individual elements of each system were distributed in several (2–3) genomic loci per pair. Although the *Mmc* genomes had a high degree of synteny, the distribution of the TA modules was rather diverse (**Fig 1**). This indicates that horizontal gene transfer (HGT) is likely to be involved in their distribution. To test a possible role of horizontal gene transfer in the dispersal of TA pairs, we constructed a phylogenetic tree of the 16 *M*. *mycoides* subsp. *capri* strains studied based on whole genome sequence data (**Fig 1**) and compared the tree with phylogenetic trees obtained from the flanking regions of the different insertion sites of the pairs (**Fig 2**). Since the three individual trees of the flanking regions (**Fig 2A–2C**) are not congruent, and the fingerprints of TA pairs are not in line with the phylogenetic tree based on whole genome data (**Fig 1**), we conclude that horizontal gene transfer shaped the distribution of the TA pairs. It is well known that integrative conjugative elements in mycoplasmas, including *Mmc*, are the main vehicle for horizontal gene transfer [50], contributing to the buildup of mosaic genomes under selective pressure [51]. A closer look at the genome loci that harbored the TA pairs (**S2 Fig**) revealed dynamic loci characterized by the presence and absence of TA systems and individual toxin- or antitoxin-encoding genes. In the case of the TA pair Fic/RelB, we noticed that several strains harbored the *yafQ* gene, downstream the *relB* gene. *yafQ* encodes a toxin, known to form a TA pair with *relB* encoding an antitoxin in other bacteria, which indicates an ongoing remodeling of TA elements in mycoplasmas. These regions were associated with features often found in genomic islands such as IS elements and bordering tRNAs [52]. Interestingly, the AbiEii/AbiEi pair has been reported to be part of the *vpma* locus, a genetic island encoding phase variable surface proteins that are key for colonization of the host and immune escape [52] present in *M*. *agalactiae* 5632 and *M*. *bovis* PG45. We also found evidence that for instance TA pairs such as the AAA-ATPase/subtilisin-like protease had flanking direct repeats (**S2A Fig**) indicating scars of previous recombination events. We detected the three TA systems in other mycoplasmas (**Fig 6A**), confirming that TA systems are widespread in mycoplasmas and that several *Mycoplasma* species harbor more than one TA system. Additionally, we experimentally confirmed functionality of homologs of the AAA-ATPase/subtilisin-like protease pair in pathogenic mycoplasmas such as *M*. *feriruminatoris*, and the more distantly related *M*. *bovis* and *M*. *gallisepticum*.

In conclusion, this study confirmed functionality of three candidate TA pairs, chromosomally-encoded in *Mmc* GM12. Our study revealed a level of plasticity of the mycoplasmas’ genomes with respect to TA systems. Since mycoplasmas are minimal organisms [2] and a few mycoplasma species have been amendable to synthetic genomics techniques [35,53], their TA systems should be investigated with respect to their effect on host-pathogen interactions using the native host [54,55]. Due to their small genome size, mycoplasmas are likely to contain only a limited number of TA systems. Therefore, they can constitute a reliable model organism to shed light on the role of TA systems in the biology of other mycoplasma or gram-positive pathogens.

## Materials and methods

### Bacterial strains and culture conditions

NEB 5-alpha (New England Biolabs) and Stellar (Clontech) chemically competent cells were used as host strains for all the cloning experiments and plasmid propagation described in this study. *E*. *coli* cells were cultured in Luria-Bertani (LB) medium at 37°C under agitation (200 rpm) or on LB agar plates supplemented with 50 μg/mL of ampicillin. *E*. *coli* strain LMG194 (F^−^ Δ*lacX74 galE thi rpsL* Δ*phoA* (PvuII) Δ*ara714 leu*::Tn*10*) and strain MG1655 (F- lambda- *ilvG*- *rfb*-50 *rph*-1) were used to test the function of the different mycoplasma candidate TA systems.

Fourteen isolates of *Mmc* (**Table 2**), were grown in SP5 liquid medium at 37°C under static conditions [35]. Restriction-free *Mycoplasma capricolum subsp*. *capricolum* (*Mcap*ΔRE) [56], originating from the *Mcap* strain California Kid^T^ (ATCC 27343), was used in this study for the selection and propagation of all the mycoplasma transformants obtained in this study. Wild-type *Mycoplasma feriruminatoris* strain G5847^T^ (*Mferi*) [57] and wild-type *Mycoplasma bovis* strain PG45 (ATCC 25523) [58] were cultured in SP5 medium whereas wild-type *Mycoplasma gallisepticum* strain PG31 (ATCC 19610) was grown using avian-specific Mycoplasma Experience medium (Mycoplasma Experience Ltd), respectively. All strains were incubated at 37°C under static conditions with 5% (v/v) CO_2_. For transformation experiments, *Mcap*ΔRE cells were grown in SOB medium at 30°C as previously described [35]. Tetracycline and chloramphenicol were added to the medium when needed at 5 and 15 μg/mL, respectively.

### *In silico* identification of candidate toxin-antitoxin (TA) systems

Identification of candidate TA systems in *Mmc* strain GM12 [4] was performed by employing the TASmania discovery pipeline [24], which uses data mining on the EnsemblBacteria database [59] to identify candidate toxin and antitoxin proteins. Selection criteria were the following: identified with an e-value lower than 1e-07, be part of a protein family (Pfam) already containing known TA systems and be found in a genomic loci containing a putative TA partner (preferentially as a neighboring gene). If candidates matched the first two criteria but were found without a counterpart, the latter were manually searched using ‘guilt by association’. Intergenic regions close to toxin and antitoxin genes on the *Mmc* GM12 genome were analyzed using Softberry’s BPROM (Softberry Inc., Mt. Kisco, NY) to identify putative promotor regions.

### Whole genome sequencing, assembly and annotation of *Mycoplasma mycoides* subsp. *capri* strains

*Mmc* cultures grown in SP5 medium were pelleted at 4,255 x *g* at 10°C for 15 min. Genomic DNA was extracted using the Wizard Genomic DNA purification kit (Promega) per vendor’s protocol. DNA concentration was determined using a Qubit 4.0 fluorometer (ThermoFischer Scientific) and purity was assessed by electrophoresis using agarose gels. If the presence of plasmids was detected, plasmid DNA was extracted from 10 ml overnight culture using the QIAprep Spin Miniprep Kit (QIAGEN) with minor adaptations including an additional washing step using buffer PB and doubling the recommended volumes of buffers P1, P2 and P3. Subsequent libraries preparation and sequencing were carried out at the Lausanne Genomic Technologies Facility. Briefly, genomic DNA was sheared in a Covaris g-TUBE (Covaris, Woburn, MA, USA) to obtain 10 kbp fragments and size fragmentation was confirmed on a Fragment Analyzer (Advanced Analytical Technologies, Ames, IA, USA). A barcoded SMRTbell library was prepared using 480 ng of genomic DNA using the PacBio SMRTbell Template Express Prep Kit 2.0 (Pacific Biosciences, Menlo Park, CA, USA) according to manufacturer’s recommendations. Libraries were pooled and sequenced with v3.0/v3.0 chemistry on a PacBio Sequel instrument (Pacific Biosciences, Menlo Park, CA, USA) at 10 hours movie time, pre-extension time of 2 hours, using one SMRT cell v3. Genomes were assembled from the PacBio reads using the software Flye, version 2.6 [60]. Circularized genomes were polished for three rounds using Arrow [single-molecule real-time (SMRT) Link version 8 package]. Genomes were rotated to the first nucleotide of the start codon of the *dnaA* gene. Sequences were then annotated using Prokka, version 1.13 [61]. The program Mauve (version 20150226) [62] was used to align and compare the newly sequence *Mmc* genomes using default parameters.

### Phylogenetic and motifs analyses for the characterization of TA systems in mycoplasmas

Both phylogenetic trees were built using Bionumerics v. 8.0 (Applied Maths, Biomèrieux) using default parameters. Whole genome sequences of the 14 sequenced *Mycoplasma mycoides* subsp. *capri* strains, plus GM12 (GenBank accession number CP001621.1) and 95010 (GenBank accession number FQ377874.1) strains, were used to generate the phylogenetic tree. *Mycoplasma mycoides* subsp. *mycoides* strain Gladysdale (Genbank accession number NC_021025.1) was included as an outgroup. Evolutionary distances were calculated using the Bionumerics comparative genomics tool (Applied Maths, Biomèrieux) and standard parameters. The phylogenetic tree of selected representatives of the *Mollicutes* (**Fig 6A**) was built using 16S rRNA sequences retrieved from Genbank (**S4 File**). All corresponding accession numbers are listed in the corresponding figure. A Jukes & Cantor correction was applied and the Neighbor-joining inference method was used.

The search for putative candidate genes on the newly annotated *Mmc* genomes was done using TBLASTN with *Mmc* GM12 amino acid sequences of candidate toxins and antitoxins as queries. All hits with both percentage identity and query coverage >80% were considered as putative orthologs. If a candidate was not found associated with a cognate protein using this approach, a Pfam search of the genes upstream and downstream of the candidate was performed to confirm the absence of a potential partner. A similar search was done to assess the presence of *Mmc* TA orthologs in other members of the *Mollicutes* using the dedicated database Molligen 4.0 [63]. Gene Graphics [64] was used to visualize the candidate surroundings on the newly sequenced *Mmc* genomes. Conservation analysis of the genes flanking the candidate TA systems and their copies was performed with the software FlaGs local version 1.2.6 [65] with its default parameters.

### Detection of transcripts for the candidate TA systems in mycoplasmas by RT-PCR

*Mycoplasma mycoides* subsp. *capri* (*Mmc*) strain GM12 was grown in 10 ml SP5 medium at 37°C until the culture reached pH 6.5. Subsequently, the culture was centrifuged at 4,255 x *g* at 10°C for 15 min. The supernatant was discarded and the bacterial cell pellet was used for RNA isolation. RNA was isolated using the Zymo Research Quick-RNA Fungal/Bacterial Miniprep kit. Briefly, 800 μl of RNA Lysis Buffer were added to the pellet and the protocol was followed starting at step 4 of the manual. After isolation, a DNase treatment was performed using the Clean & Concentrator -5 kit (ZymoResearch), omitting the first step of the protocol (addition of Binding Buffer) and using the IICR columns provided in the kit. Reverse transcription PCR was performed using SuperScript IV RT Mix (Invitrogen) and 2x Platinum SuperFi RT-PCR Master Mix (Invitrogen) using primers (**S3 Table**, primers 1–12). In brief, 12.5 μl of 2x Platinum SuperFi RT-PCR Master Mix, 0.25 μl SuperScript IV RT Mix, 1.25 μl per primer (10 μM), 1μl DNase treated RNA (~ 32 ng) and water up to a volume of 25 μl were mixed per sample. Amplicons were visualized on a 1% agarose gel.

### RNA-sequencing (RNA-seq) and operon predictions

For the transcriptomics analysis, three biological replicates were used. *Mmc* GM12 was grown in 10 ml SP5 medium at 37°C under static conditions until the culture reached a pH 6.5. Total RNA was isolated as described above. The integrity of the RNA was checked via electrophoresis on a 1% agarose gel. RNA preparations were sent to the Lausanne sequencing platform. RNA quality was assessed on a Fragment Analyzer (Agilent Technologies) and the RNAs had an RQN between 7.5 and 8.5. RNA-seq libraries were prepared from total RNA with the Illumina TruSeq Stranded mRNA reagents (Illumina), omitting the polyA selection step and using a unique dual indexing strategy. Ribosomal rRNA depletion was done with QIAseq FastSelect– 5S/16S/23S kit (Qiagen), with 400ng as total RNA input. Libraries were quantified by a fluorimetric method (QubIT, Life Technologies) and their quality was assessed on a Fragment Analyzer (Agilent Technologies). Cluster generation was performed with 1.92 nM of an equimolar pool from the resulting libraries using the Illumina HiSeq 3000/4000 SR Cluster Kit reagents and sequenced on the Illumina HiSeq 4000 using HiSeq 3000/4000 SBS Kit reagents for 2 x 150 cycles (paired end). Sequencing data were demultiplexed using the bcl2fastq2 Conversion Software (v. 2.20, Illumina).

Two methods were used to predict operons using the reference genome of *Mycoplasma mycoides* subsp. *capri* GM12 (CP012387.1) and the paired-end RNAseq data of 3 biological replicates described above. First, RockHopper [66] was applied using Genbank annotation files of the reference genome previously converted into ptt and rnt file formats via a homemade python script. The reads were aligned to the reference genome and counted for each gene to identify the transcripts coverage. Operons were identified by combining the probability of the smoothed distribution of the intergenic distance between two genes on same strand and orientation, and the similarity of the expression values between the two genes. The output was given as a tab delimited file listing the potential operons. Then, ANNOgesic [67] was applied in parallel. It started by remapping reads to the reference genome with its sister tool READemption [68] requiring the annotations in gff format, and then it revealed the operons in two steps. First it identified the transcripts and secondly the operons based on the TSS (transcript start site) and the transcripts coverage. The output was provided as a gff or a csv file listing the potential operons.

### Proteomic analysis

*Mmc* strain GM12 was grown in SP5 media until mid-logarithmic phase at 37°C under static conditions. A third of the culture was removed and subsequently processed as the “log phase” sample. The remaining culture was split and each subculture was incubated until early stationary phase at 37°C or 41.5°C and processed as “early stationary phase 37°C” and “early stationary phase 41.5°C”, respectively. Mycoplasma cells were harvested by centrifugation at 3,000 x g for 20 min at 4°C. Supernatants were decanted and the pellets washed 3 times with ice-cold PBS. Total protein concentration was measured using the Pierce BCA Protein Assay Kit (ThermoScientific) according to vendor’s instructions. The pellets were stored at -80°C until analyses could be performed. The cells were subsequently lysed via resuspension in 8M urea/100mM Tris-HCl. Proteins were then precipitated overnight at -20°C in 5 volumes of cold acetone. All liquid was carefully removed and the pellet dried in ambient air for 15 min before reconstitution of proteins to a concentration in 8M urea/50mM Tris-HCl. An aliquot corresponding to 10 μg protein was trypsinized overnight at room temperature in digestion buffer (1.6M urea, 20mM Tris-HCl, 2mM CaCl_2,_ pH8). Enzymatic digestion was stopped by adding 1% (v/v) tri-fluoroacetic (TFA). Three repetitive injections of an aliquot corresponding to 500 ng of digested proteins were processed by liquid chromatography (LC)-MS/MS (PROXEON coupled to a QExactive HF mass spectrometer, ThermoFisher Scientific). The mass spectrometry proteomic data have been analyzed against custom databases by Transproteomic pipeline (TPP) tools [69]. Four database search engines were used: Comet [70], Xtandem [71], MSGF [72] and MyriMatch [73]. Each search was followed by the application of the PeptideProphet tool [74]; the iProphet [75] tool was then used to combine the search results, which were filtered at the false discovery rate of 0.01; furthermore, the identification was only accepted if at least two of the search engines agreed on the identification. The decoy approach was used for such custom databases containing standard entries. Protein inference was performed with ProteinProphet. For those protein groups accepted by a false discovery rate filter of 0.01, a Normalized Spectral Abundance Factor (NSAF) [76] was calculated based on the peptide to spectrum match count; shared peptides were accounted for by the method published elsewhere [77].

### Plasmid constructions

All the plasmid constructions done in this study are summarized in the **S4 Table**.

*E*. *coli*-codon optimized sequence-verified synthetic genes, encoding either candidate mycoplasma toxins or antitoxins, were chemically synthesized (GenScript, **S6 File)**. Synthetic DNA cassettes were PCR-amplified using primers containing *EcoRI* and *SacI* restriction sites (**S3 Table**, primers No. 1–6) and the Q5 High-Fidelity DNA polymerase (NEB) per manufacturer’s instructions. PCR amplicons were purified, digested with both *EcoRI* and *SacI* (NEB) in 50-uL reactions and purified once again. All DNA fragments were ligated into the pBAD/His, previously digested with the same restriction enzymes, using T4 Ligase (Promega) and transformed in *E*. *coli* using standard protocols. Resulting plasmids were isolated using the QIAprep spin miniprep kit (Qiagen), sequence-verified and stored at -20°C for further use.

The plasmid pET28a-0132-pAra0133 was constructed using the NEBuilder HiFi DNA Assembly kit (NEB) following manufacturer’s instructions. Three DNA cassettes consisting of i) the 3,700 bp *araBAD*-MMCAP_0133 region amplified from the pBAD-0133 construct, ii) the 1,062 bp codon-optimized MMCAP2_0132 DNA cassette and iii) the remaining 5,255 bp of the pET28a backbone (Novagen) were PCR-amplified using the Q5 High-Fidelity DNA polymerase (NEB) and primers 23–28 (**S3 Table**). Overlapping amplicons were ligated and transformed in *E*. *coli* as previously described.

The *oriC*-based pMYCO1 [34] was used as a backbone for the testing of all TA candidates in mycoplasmas. Primers 79 to 101 were used to PCR-amplify the corresponding genes as well as the pMYCO1 backbone. The sequence of the spiralin promoter was amplified from the pMT85tetM-PSlacZ-pRS313 [35]. PCR amplification of the candidate mycoplasma TA systems were done using genomic DNA of *Mycoplasma mycoides* subsp. *capri* strain GM12 purified using the Wizard genomic DNA purification kit (Promega) per manufacturer’s instructions. Constructs were obtained in *E*. *coli* using the NEBuilder HiFi DNA Assembly kit (NEB). Constructs carrying the putative toxin or entire TA system of *M*. *feriruminatoris*, *M*. *bovis* and *M*. *gallisepticum* were done as previously described using primers 111 to 128 (**S3 Table**).

The pMYCO1-Chlo^R^-pXyl/tetO_2_ was also constructed to test the capacity of the three candidate toxins to trigger cell death in mycoplasmas. The DNA cassette carrying the tetracycline inducible system was chemically synthetized based on the original design of the pXTST developed in *Spiroplasma citri* [37]. The chloramphenicol resistance gene (*cat*) was amplified from the pCC1BAC-His3 [53]. Primers used to amplify all the different DNA parts, as well as the genes encoding the three mycoplasma toxins are listed (**S3 Table**, primers 129 to 144). Overlapping amplicons were ligated using the NEBuilder HiFi DNA Assembly kit (NEB) following manufacturer’s instructions and transformed in *E*. *coli*. Resulting plasmids were isolated, sequence-verified and subsequently transformed into *Mcap*ΔRE for functional studies.

### Functionality assays in *E*. *coli*

Sequence-verified plasmids were heat shock-transformed into chemically competent *E*. *coli* LMG194 for subsequent functionality studies. *E*. *coli* clones containing the different candidate toxins and antitoxins were grown overnight in LB medium containing glucose (0.2%) supplemented with 50 μg/mL of ampicillin at 37°C under agitation. The next day, overnight cultures were diluted 100-fold in fresh medium and split in two sub-cultures where glucose (0.2% (v/v)) or arabinose (0.2% (v/v)) was added, respectively. Cultures were incubated at 37°C for 5 hours and OD_600_ measurements were taken every hour. Experiments were conducted in triplicate for each construct. At 5 hours post-induction, cultures were serially diluted up to 10^−6^ and 10 μL of each dilution were spotted onto LB agarose plates containing either glucose (0.2%) or arabinose (0.2%). Plates were incubated overnight at 37°C and photographed using the BioRad Molecular Imager Gel Doc XR System (BioRad). A neutralization assay was carried out for the TA_132/3_ using a dual expression system in which the expression of the T133 is under the dependence of arabinose whereas the A132 is expressed in the presence of IPTG. Overnight cultures were diluted 100-fold in fresh LB-Amp50 medium containing either IPTG only (1mM), arabinose only (0.2%) or IPTG (1mM) and arabinose (0.2%). Cultures were monitored for 5 hours as previously done for the individual candidates. At 5 hours post-induction, cultures were spot-diluted on LB agar plates supplemented with appropriate inducers and photographed the next day.

### Detection of recombinant proteins using immunoblots

Heterologous expression of the His_6_-tagged candidate mycoplasma toxins and antitoxins in *E*. *coli* was verified by immunoblot using anti-His antibodies as previously described [78]. Arabinose-induced cultures were grown at 37°C under agitation for 5–6 hours and 1-mL samples were removed every hour for subsequent analysis. A non-induced culture, where arabinose was replaced with glucose, was used as control. Protein concentration was determined using the Pierce BCA Protein Assay Kit (ThermoScientific). Immunoblotting was basically carried out as described recently [78]. Briefly, 0.1 mg of proteins in the cell pellet were separated onto a 12% SDS-PAGE gel and transferred to nitrocellulose membranes using the Trans-Blot SD semi-dry transfer system (Bio-rad) at 25 V in Towbin transfer buffer (25 mM Tris, 192 mM Glycine and 20% (v/v) methanol) using standard procedures [79]. Membranes were blocked in PBS supplemented with 0.1% Tween-20 (Merck) and 2% BSA (Sigma) and subsequently probed with monoclonal mouse-derived anti-His antibody (LS-C57341, LsBio, diluted 1:1,000) followed by HRP-conjugated goat anti-mouse IgG antibody (AP308P, Sigma, diluted 1:70,000) as primary and secondary antibodies, respectively. Three consecutive 10-min washes in PBS supplemented with 0.1% Tween-20 were carried out between each step. Proteins were detected using chemiluminescence (SuperSignal West Pico PLUS, ThermoFischer Scientific) and documented using the Fusion FX system (Vilber).

### Functionality studies in *Bacillus subtilis*

*E*. *coli*-optimized synthetic versions of each mycoplasma candidate toxin and antitoxin were PCR-amplified using the Q5 High-Fidelity DNA polymerase (NEB) and primers 32–45 (**S3 Table**). Amplicons were individually ligated in the *E*. *coli* / *B*. *subtilis* pHT01 shuttle vector (MoBiTec) previously digested with *BamHI* and *XmaI*. In addition, both the toxin and antitoxin encoding genes of two candidate TA systems were inserted in the same pHT01 backbone to probe the neutralizing capacity of the candidate antitoxins. These constructions, namely pHT01-TA_132/3_ and pHT01-TA_752/3_ were constructed using the NEBuilder HiFi DNA Assembly kit (NEB) following manufacturer’s instructions and primers 58–73 (**S3 Table**). Both genes were inserted in frame with P*grac*01 promoter allowing the induction of both the toxin and antitoxin proteins by addition of IPTG. All constructs were transformed in *Bacillus subtilis* subsp. *subtilis* strain 168. Competent cells were prepared following manufacturer’s instructions (MoBiTec) and transformants were grown at 37°C in LB medium (+/- agar) supplemented with 5 μg/mL of chloramphenicol. Plasmids were isolated from *B*. *subtilis* using a modified version of the QIAprep spin miniprep kit (Qiagen). Modifications consisted in growing the *B*. *subtilis* cultures to an OD_600_ of 1 and incubate the resulting pellet in 250 μL of buffer P1 containing 1 mg/mL of lysozyme for 10 minutes at 37°C. The rest of the protocol was left unchanged. For the induction experiments, *B*. *subtilis* clones carrying the various plasmids were grown overnight at 37°C under shaking conditions in LB broth supplemented with 5 μg/ml chloramphenicol. The following morning, the overnight culture was diluted to an OD_600_ of 0.04 in 24 mL final volume of fresh LB-Chlo^R^ broth medium. The culture was then split into two 12-mL aliquots and IPTG (1 mM, final concentration) was added to one of the two cultures. Cultures were incubated at 37°C under agitation and OD_600_ measurements were taken every hour for 7 hours. Experiments were conducted in duplicate for each construct. At 5 hours post-induction, 10 μl of each culture were removed, serially diluted up to 10^−6^ and plated onto LB-Chlo^R^ agar plates. The original pHT01 plasmid was used a control. The plates were incubated overnight at 37°C and photographed using the BioRad Molecular Imager Gel Doc XR System (BioRad). Additionally, aliquots of 600 μl were collected from each strain (induced and non-induced) at 5 hours. These aliquots were used to visualize protein induction via Coomassie staining. The cells were centrifuged at 4,000 × g at 4°C for 10 minutes. The pellets were then resuspended in 200 μl Lysis Buffer, which consists of 20mM Tris, 50 mM NaCl, 1 mg/ml lysozyme and cOmplete Protease Inhibitor Cocktail (Roche Diagnostics GmbH) at pH 8.0. The resuspended cells were incubated at 37°C for 30 minutes and afterwards sonicated using the Branson 450 Sonifier (VWR). The Output Control was set to 3.5 and the samples were sonicated on ice 3 times for 5 seconds with 30 second breaks in between. 30 μl of each sample was mixed 1:1 with 2x Laemmli Sample Buffer (Bio-Rad) and subsequently boiled at 99°C for 10 minutes before being loaded onto a 12% SDS-PAGE gel. The gels were then stained with AcquaStain (Lubio Science) and photographed using the BioRad Molecular Imager Gel Doc XR System+.

### Functionality assays in mycoplasmas

All pMYCO1-based constructs were transformed into *Mcap*ΔRE as previously described [35] using 1 μg of plasmid DNA. *Mcap* was selected to probe functionality of the three candidate TA systems as none appeared to be present in its genome. Three biological replicates including three technical replicates per biological replicate were carried out. Transformants were selected on SP5 supplemented with 5 μg/mL (SP5-tet_5_) agar plates at 37°C, passaged for three consecutive rounds in 1ml SP5-tet_5_ broth medium and finally PCR-screened using primer pairs specific for the inserted TA elements, with select clones being further sequence verified (**S3 Table**, primers 50–58, 108–109, 145–162). Numbers of transformants were used to analyze the different transformation rates.

### Use of a tetracycline-inducible expression system to trigger cell death in mycoplasmas

Mycoplasma clones transformed with the pMYCO1-Chlo^R^-pXyl/tetO_2_ containing each toxin-encoding gene were grown in SP5-Chlo^R^ at 37°C under static conditions until late logarithmic phase. The culture was then split in two aliquots. The first aliquot was induced with 1 μg/mL tetracycline (Sigma), while the other was left uninduced. Both subcultures were then allowed to grow at 37°C and 20-μL samples were taken at time points 0, 3, 6, 9, 12, 24, 36, and 48 hours. After a ten-fold serial dilution in SP5 medium (up to 10^−6^), all samples were incubated at 37°C in 96-well flat bottom plates and growth was assessed using the CCUs method. At time points 0 and 24 hours, cultures were additionally plated on SP5 plates and CFUs were counted to calculate the bacterial concentration. This experiment consisted of 3 biological replicates and 2 technical replicates.

### Scanning electron microscopy (SEM) of *E*. *coli* and *B*. *subtilis* clones expressing the candidate toxins

The morphology of *E*. *coli* and *B*. *subtilis* cells upon toxin induction was investigated by scanning electron microscopy. At 5 hours post-induction, samples (1 mL) from arabinose-induced *E*. *coli* cultures were centrifuged at 1,500 x *g* at room temperature for 3 min. Pellets were washed 3 times with 1 mL of PBS and finally resuspended in 250 μL of PBS. Subsequently, 250 μL of 5% glutaraldehyde (Merck) in 0.2 M cacodylate buffer, pH 7.4, was slowly added to the samples with gentle agitation. The tubes were stored at 4°C until further processing. Fixed cells were cytospun onto platinum-sputtered and PLL-coated coverslips (40 μl at 125 x *g*, 5 min). The coverslips were then washed 3 times with 0.1M cacodylate buffer, pH 7.4, postfixed in 1% OsO4 (Polysciences) in 0.1M cacodylate buffer for 30 min, washed 3 times again and dehydrated in an ascending series of ethanol (70%, 80%, 94%, 100%, 100%, 100% ethanol at room temperature for 15 min each). Samples were coated with 15 nm of platinum in a high vacuum coating unit (CCU-010; Safematic) and examined with a scanning electron microscope DSM 982 Gemini (Zeiss) at an accelerating voltage of 5 kV at a working distance of 4 mm.

### Statistical analyses

Functionality assays in *E*. *coli* were carried out in three biological replicates (each biological replicate consisted of three technical replicates). To assess the significance of the heterologous expression on growth of the clones at every time point, we applied a three-way (time, treatment, construct) repeated-measures ANOVA and decomposed into post hoc tests based on pairwise comparisons with a Bonferroni adjustment of the p-value. The statistical analysis was done using R version 3.6.2.

Functionality assays in mycoplasmas were also carried out in three biological replicates, each including three technical replicates.

Student’s t-test in R was used for the comparison of the transformation rates obtained with mycoplasma clones harboring the complete TA systems versus those harboring the toxin only. Additionally, an ordinary one-way ANOVA test with Tukey’s multiple comparison test was applied to compare transformation rates obtained with mycoplasma clones harboring either the complete TA system or the antitoxin counterpart using GraphPad Prism version 9.2.0 (San Diego, California USA).

## Supporting information

S1 Fig Detection of transcripts of candidate TA systems. Complementary DNA (cDNA) was used as template to detect the presence of (A) individual (T or A) transcripts or (B) overlapping TA transcripts. Genomic DNA (gDNA) was used as template to confirm the absence of DNA in the RNA preparations with the same primers used for (C) individual TA partners or (D) overlapping TA fragments. PCR amplifications were conducted as previously described and amplicons were separated on 1% agarose gels. M: 1 kbp GeneRuler DNA ladder.
(PDF)

S2 Fig MAUVE alignment of 16 *Mmc* genomes. Multiple sequence alignment of different *M*. *mycoides* subsp. *capri* genomes using Progressive MAUVE and default parameters. Colored blocks are collinear and homologous regions. Inversions of colinear blocks are displayed below the center line of the genome. Strain names are marked at the left of each alignment block and positions on the genomes are marked on top.
(PDF)

S3 Fig Genomic neighborhood analysis of each genomic loci for the three candidate TA systems in *Mmc* strain GM12. The software Gene Graphics was used to visualize the neighboring genes downstream and upstream of each TA modules. Graphics are displayed for (A-C) the three genomic loci of the TA_132/3_ system, (D-E) the two genomic loci of the TA_160/1_ system and (F-G) the two genomic loci of the TA_752/3_ system.
(PDF)

S4 Fig Heterologous expression of different candidate toxins and antitoxins in *E*. *coli*. Immunoblot analysis of *E*. *coli* expressing heterologous candidate toxins and antitoxins cloned into the pBAD/His expression vector. Expression was induced by addition of arabinose and monitored during 7 hours. Total proteins was separated onto a 12% SDS PAGE before being transferred to a nitrocellulose membrane. Immunoblots were carried out using a commercially available anti-His antibody.
(PDF)

S5 Fig Characterization of the *Mmc* TA systems in *B*. *subtilis*. (A) Growth curves of *B*. *subtilis* harboring the different plasmid constructs of the TA132/3 (left), TA160/1 (middle) and TA752/3 (right) over a period of 7 hours after induction with arabinose (I) or non-induction (NI). Optical densities at 600nm were measured every hour and displayed using GraphPad Prism 9. Each data point represents the mean of three biological replicates, bars indicate standard deviation. (B) Ectopic expression of TA elements in *B*. *subtilis*, Total proteins of *B*. *subtilis* harboring the pHT01-T752 non-induced (lane 1), the pHT01-T752 induced (lane 2), the pHT01-A753 non-induced (lane 3), the pHT01-A753 induced (lane 4), the pHT01-A132 non-induced (lane 5), the pHT01-A132 induced (lane 6), the pHT01-T133 non-induced (lane 7), the pHT01-T133 induced (lane 8), were checked by SDS-PAGE and visualized using Coomassie staining. Arrows indicate the induction of the recombinant proteins at the expected sizes. (C) Toxicity neutralization spot assays in response to the repression (left) or induction (right) of the heterologous expression of the different toxins, antitoxins and entire TA systems. The empty vector pHT01 was used as negative control. (D) Scanning electron micrograph (magnification 10,000x) displaying morphological changes of *B*. *subtilis* observed 5 hours after the induction of T_752_ recombinant protein. The pHT01 empty vector was used a negative control. Indentations of cells are indicated by asterisk, empty/ shrunk cells by arrowhead and shrunk cells upon division by arrows.
(PDF)

S1 Table TASmania database output file for *M*. *mycoides* subsp. *capri* GM12. (DOCX)

S2 Table PacBio sequencing of the *M*. *mycoides* subsp. *capri* strains. (DOCX)

S3 Table Oligonucleotides used in this study. (DOCX)

S4 Table Plasmid constructions used in this study. (DOCX)

S1 File TBLASTN results for presence of candidate TA systems in different *M*. *mycoides* subsp. *capri* strains. (XLSX)

S2 File Output of the FlaGs analysis. (XLSX)

S3 File Proteomics data generated in this study. (XLSX)

S4 File 16S rRNA sequences used. (DOCX)

S5 File Output of the *in silico* analyses carried out to identify TA homologs in the class of *Mollicutes*. (XLSX)

S6 File Sequences of toxin- and antitoxin genes cloned in this study. (DOCX)
