# Supplementary figures and images for: Minimalistic mycoplasmas harbor different functional toxin-antitoxin systems
Source: PLoS Genet. 2021 Oct 21;17(10):e1009365. doi: 10.1371/journal.pgen.1009365 (PMC8562856; doi:10.1371/journal.pgen.1009365)

**A**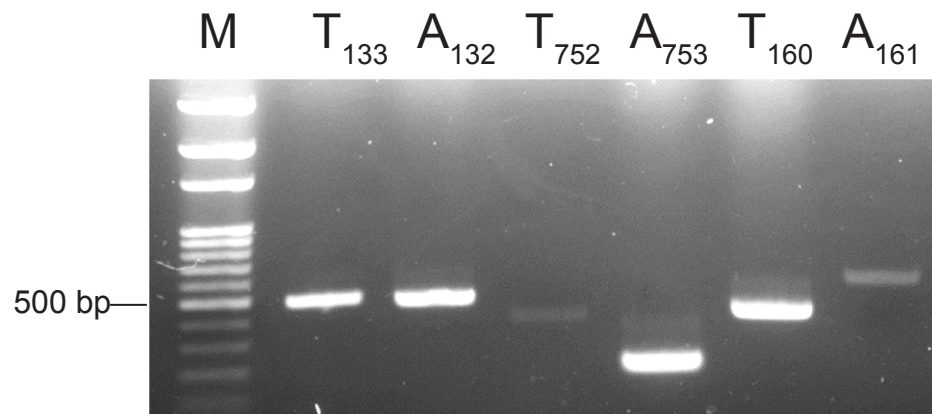**B**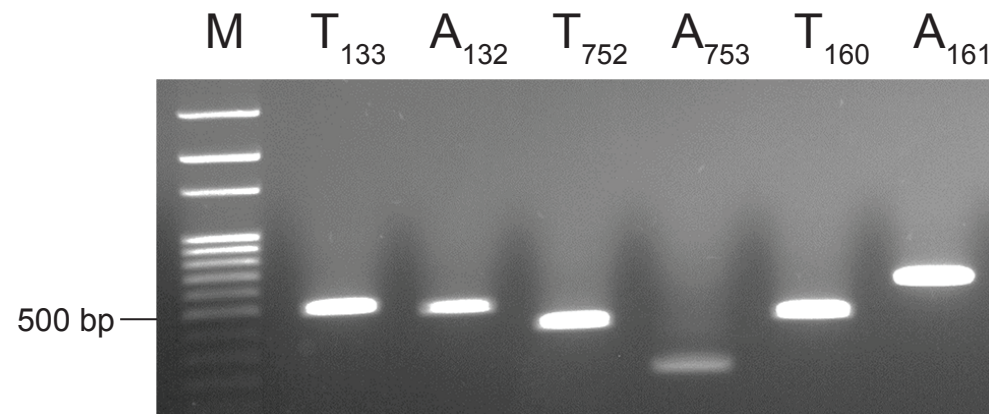**C**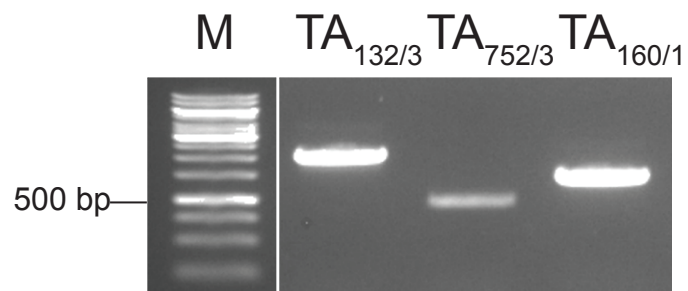**D**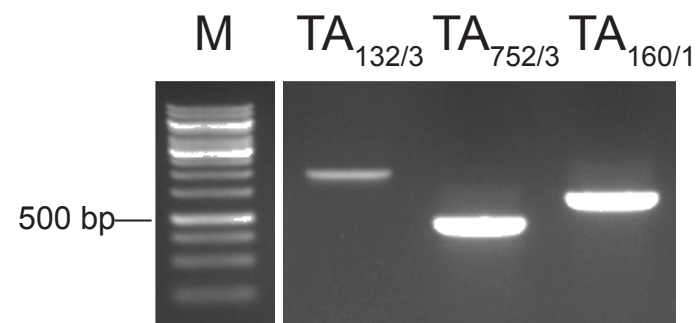

Supplement: S1 Fig — Complementary DNA (cDNA) was used as template to detect the presence of (A) individual (T or A) transcripts or (B) overlapping TA transcripts. Genomic DNA (gDNA) was used as template to confirm the absence of DNA in the RNA preparations with the same primers used for (C) individual TA partners or (D) overlapping TA fragments. PCR amplifications were conducted as previously described and amplicons were separated on 1% agarose gels. M: 1 kbp GeneRuler DNA ladder. (PDF) [file pgen.1009365.s001.pdf]

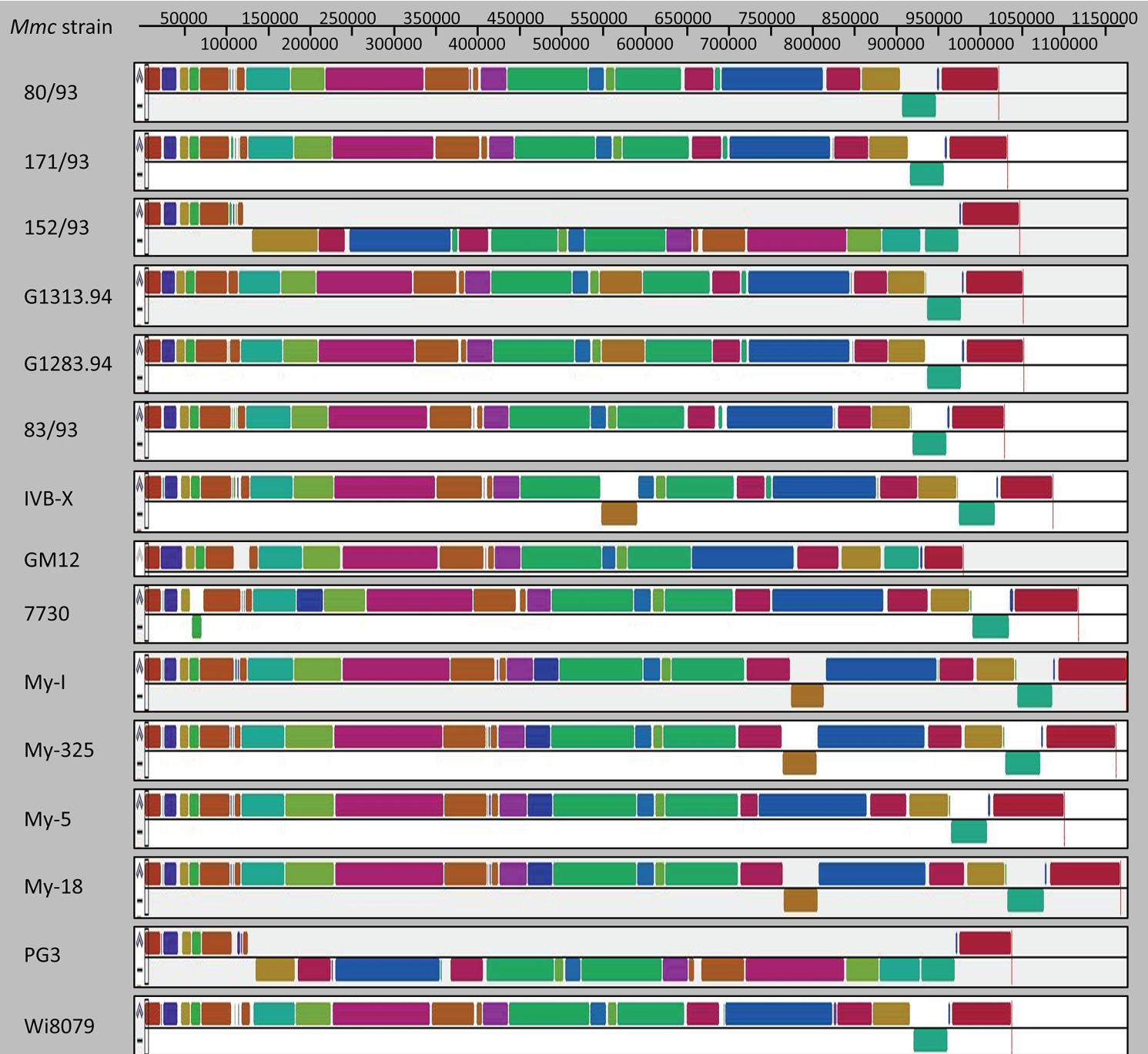

Supplement: S2 Fig — Multiple sequence alignment of different M. mycoides subsp. capri genomes using Progressive MAUVE and default parameters. Colored blocks are collinear and homologous regions. Inversions of colinear blocks are displayed below the center line of the genome. Strain names are marked at the left of each alignment block and positions on the genomes are marked on top. (PDF) [file pgen.1009365.s002.pdf]

A

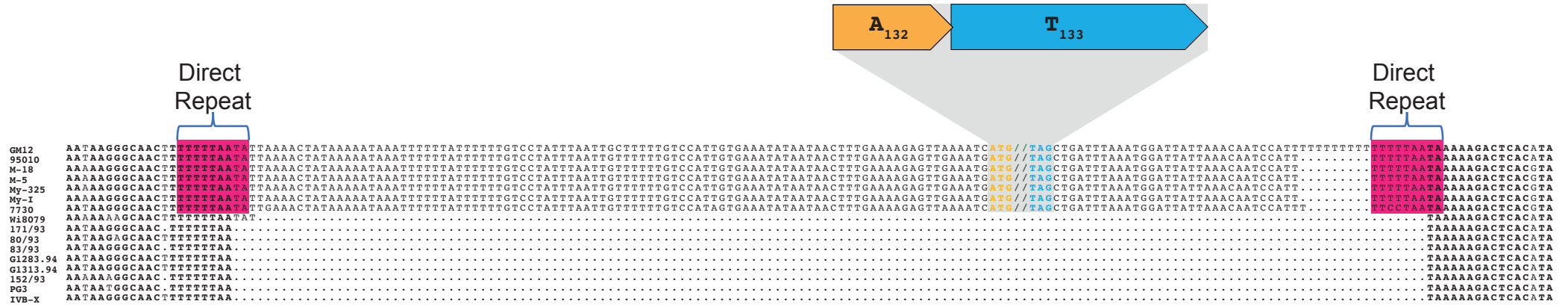

# A

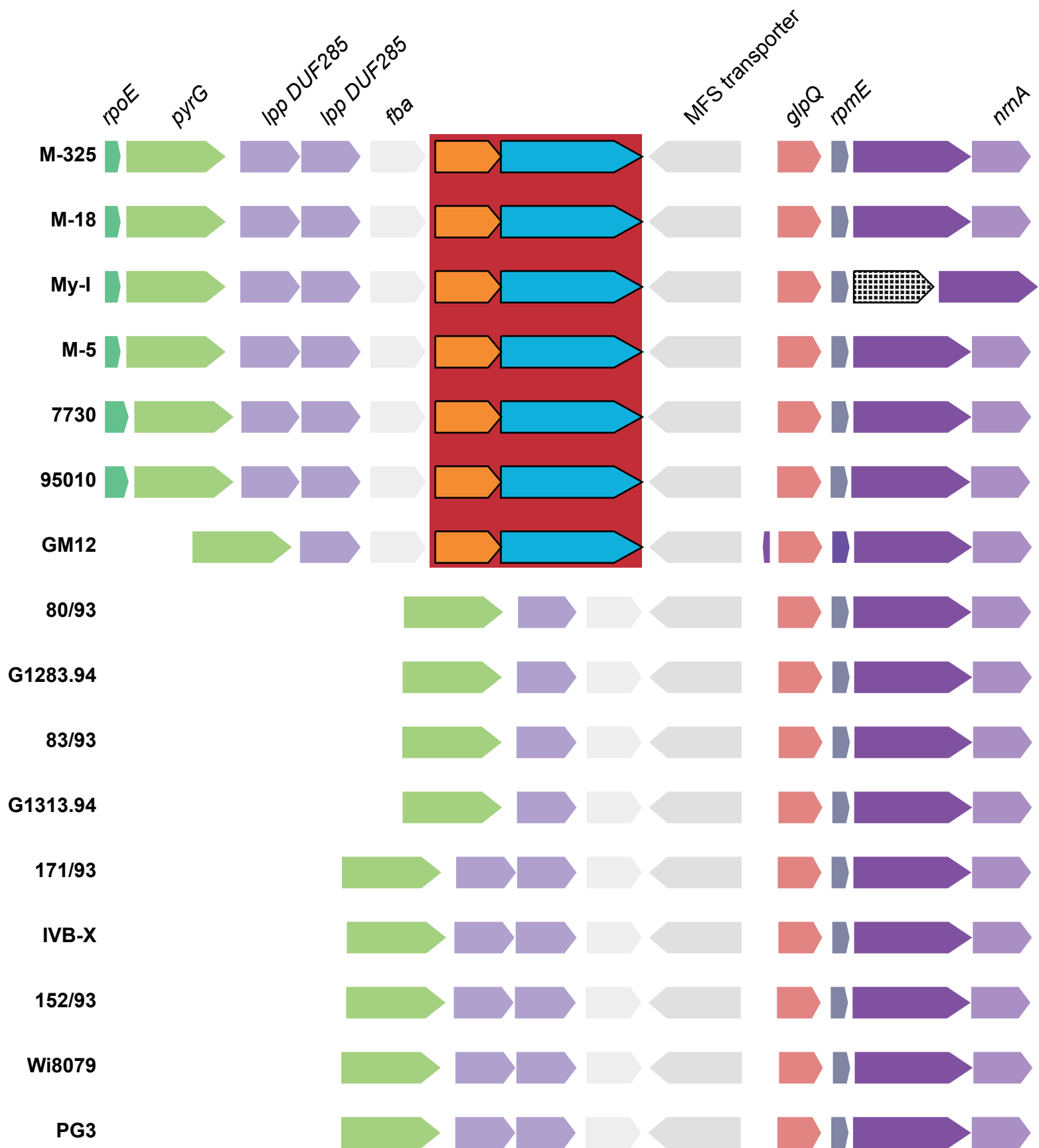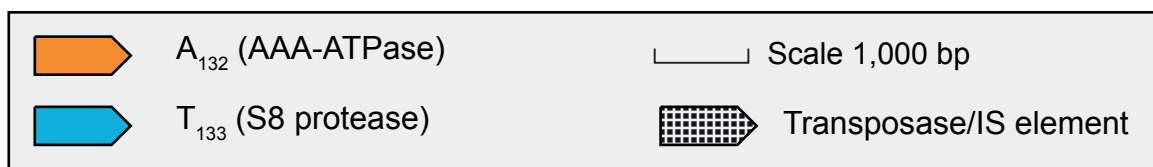

B

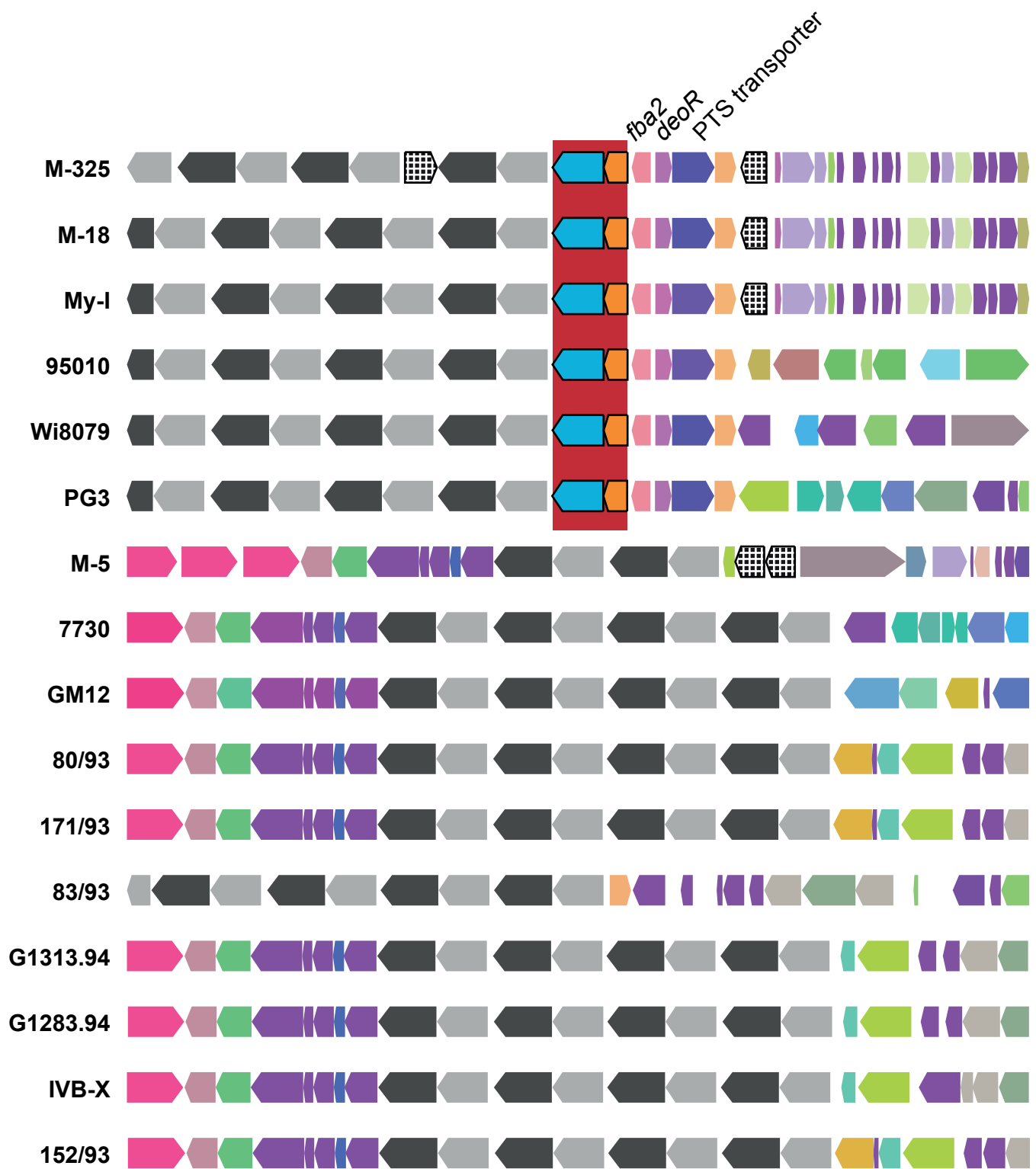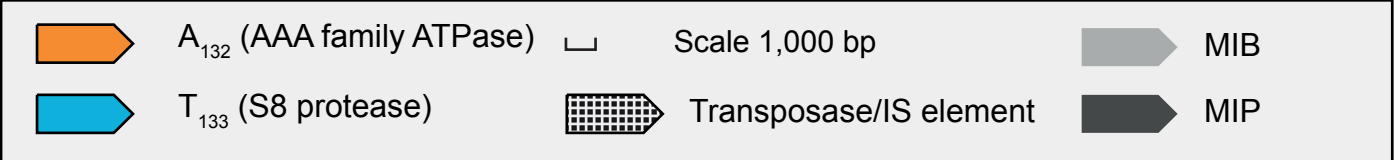

**C**

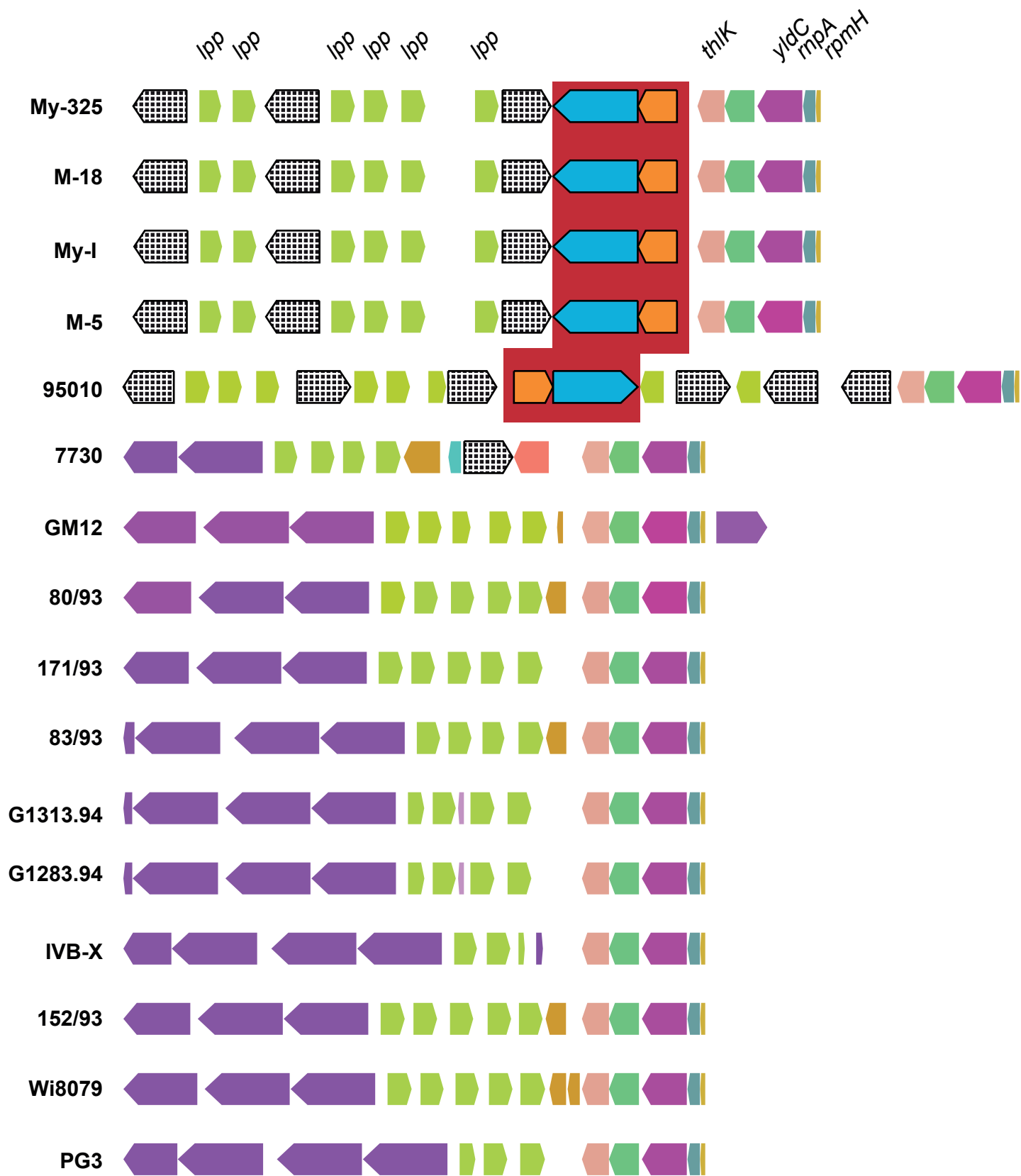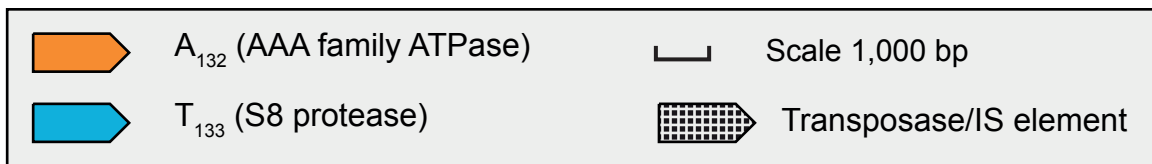

# D

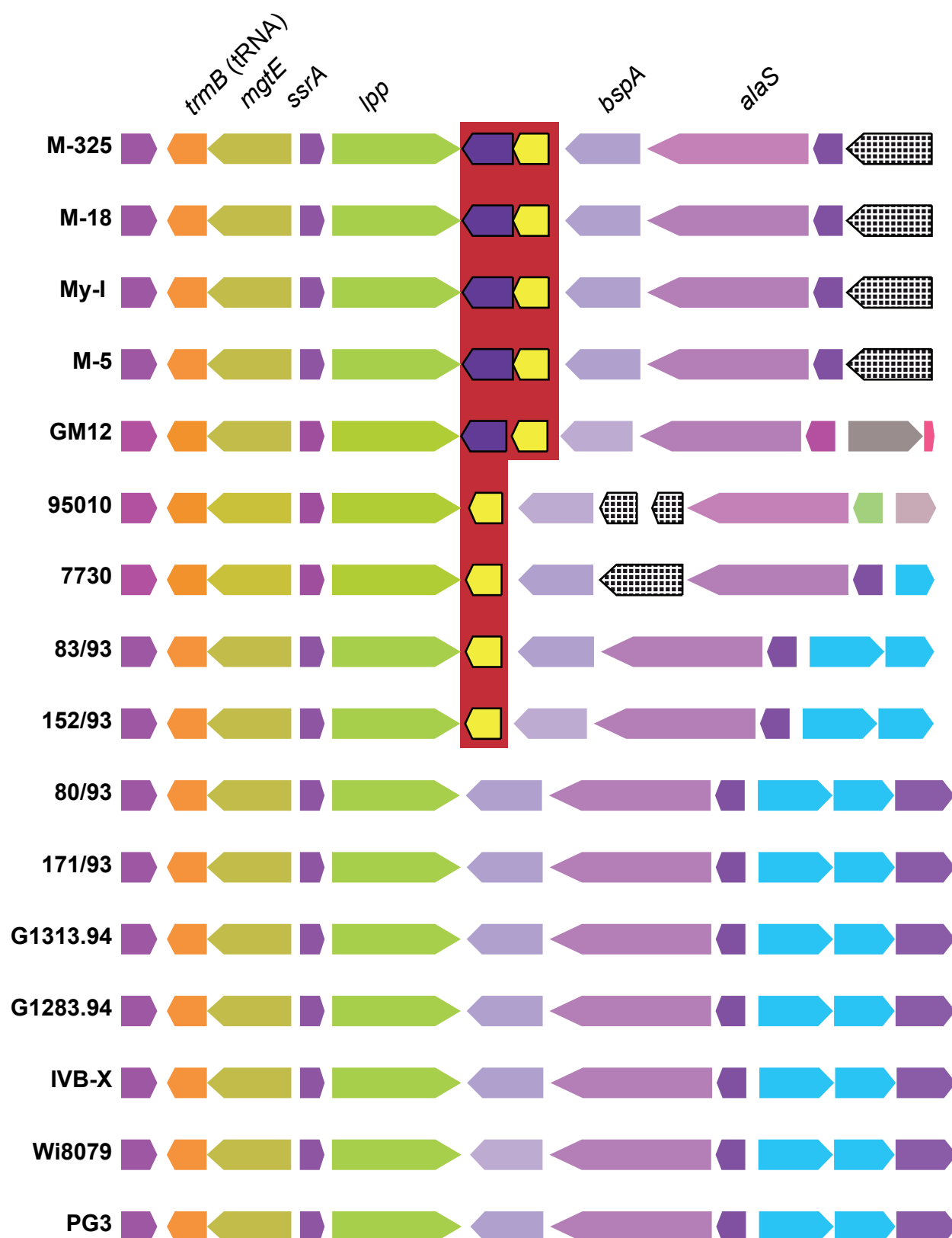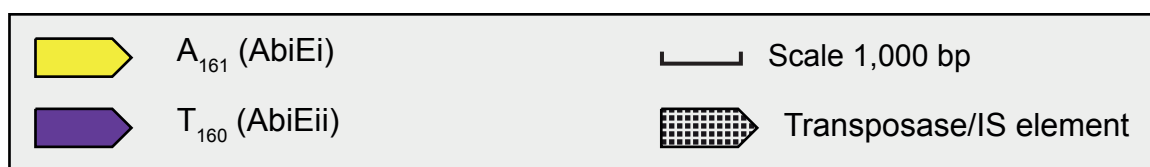

E

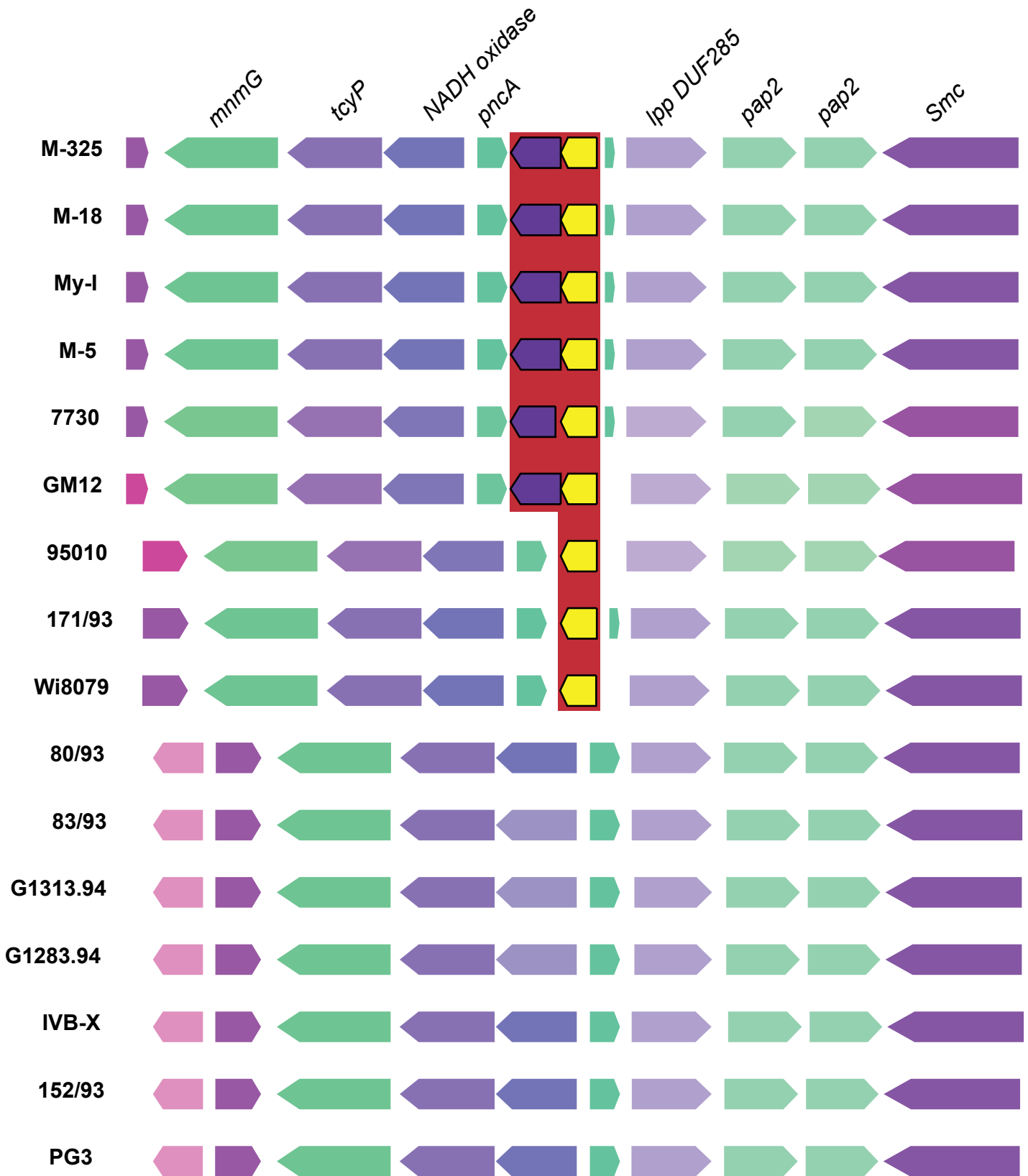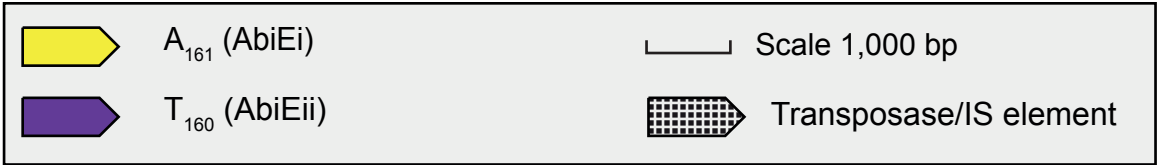

# F

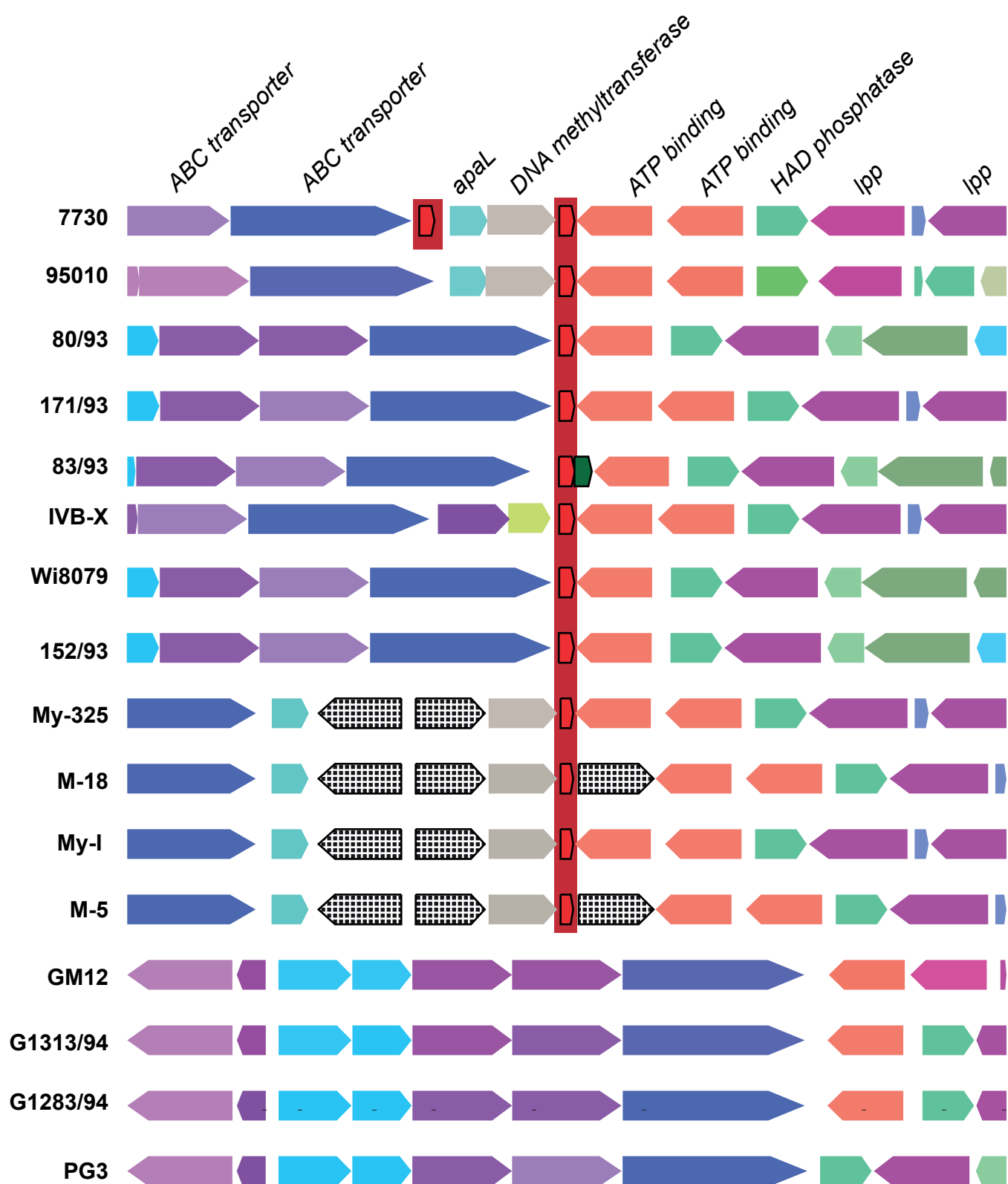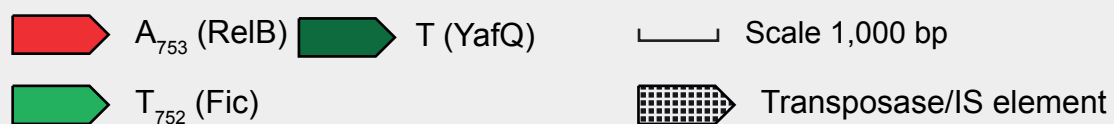

# G

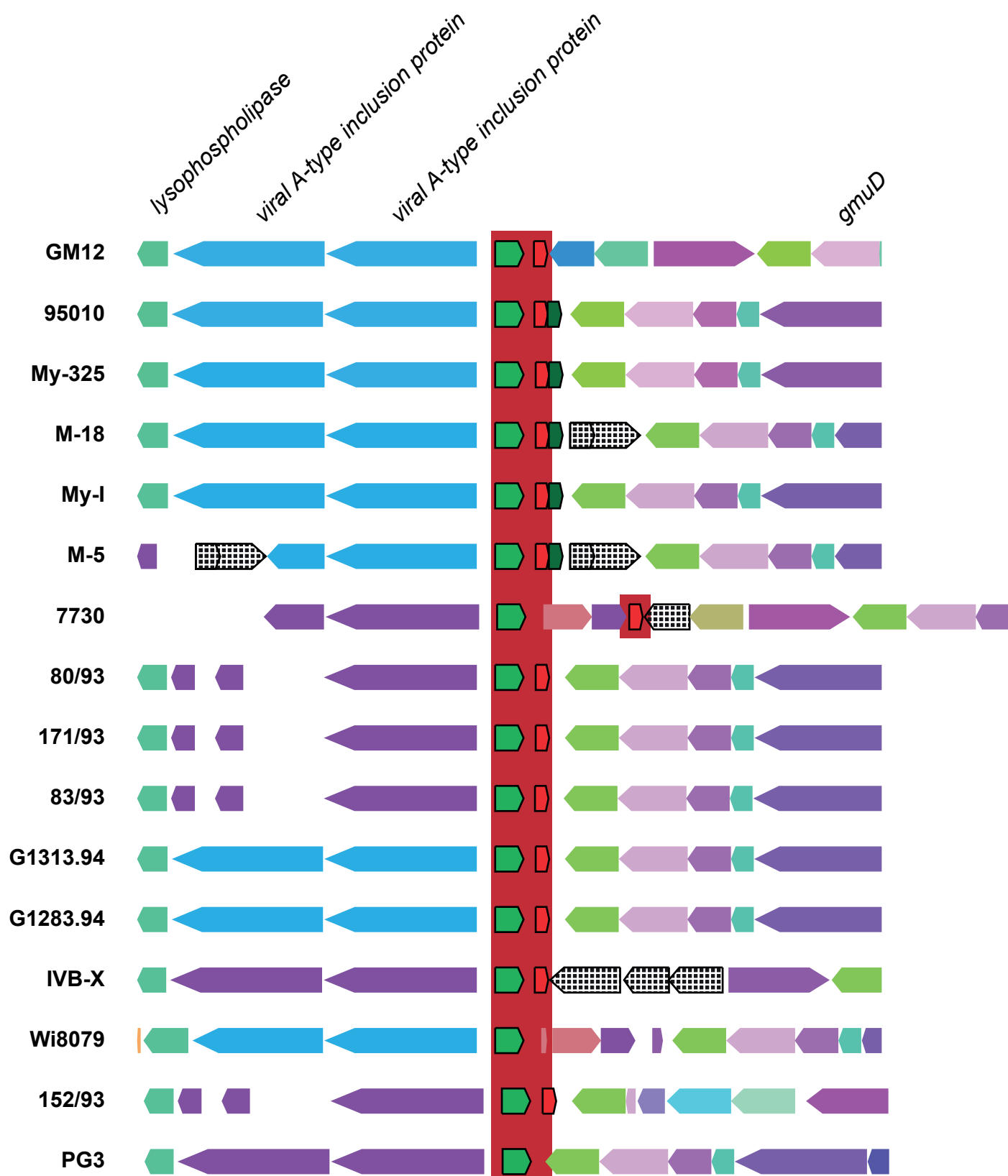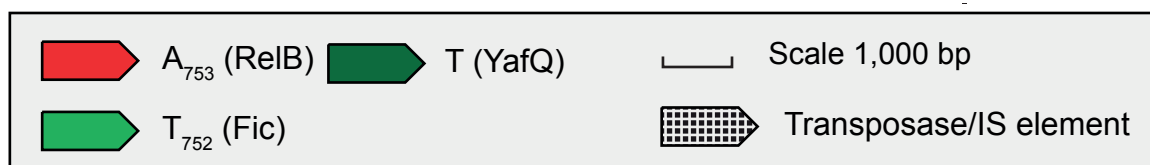

Supplement: S3 Fig — The software Gene Graphics was used to visualize the neighboring genes downstream and upstream of each TA modules. Graphics are displayed for (A-C) the three genomic loci of the TA132/3 system, (D-E) the two genomic loci of the TA160/1 system and (F-G) the two genomic loci of the TA752/3 system. (PDF) [file pgen.1009365.s003.pdf]

**T<sub>133</sub>**

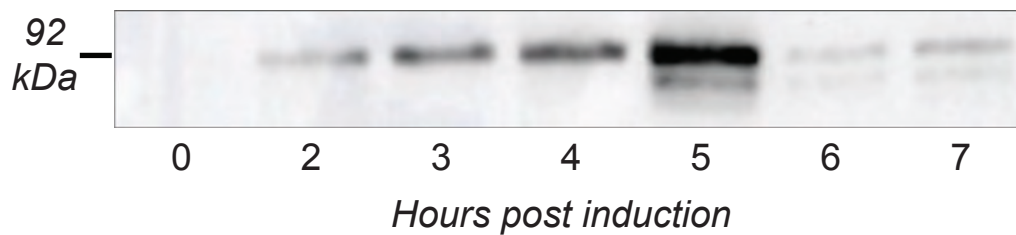

**A<sub>132</sub>**

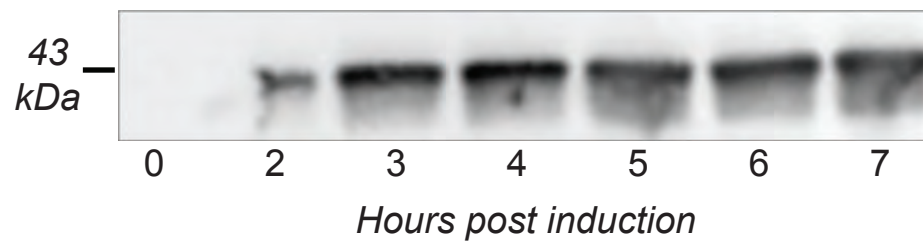

**T<sub>160</sub>**

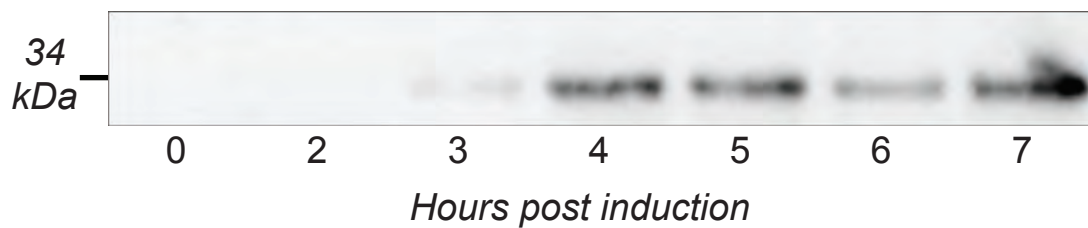

**A<sub>161</sub>**

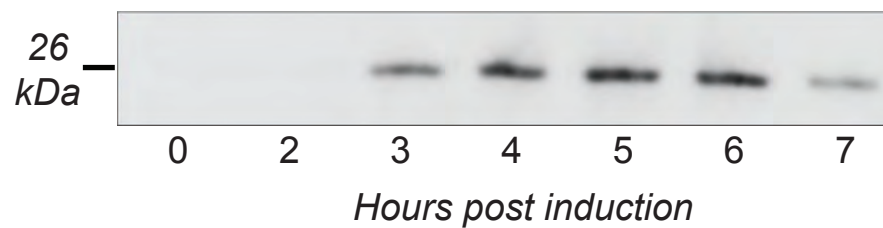

**T<sub>752</sub>**

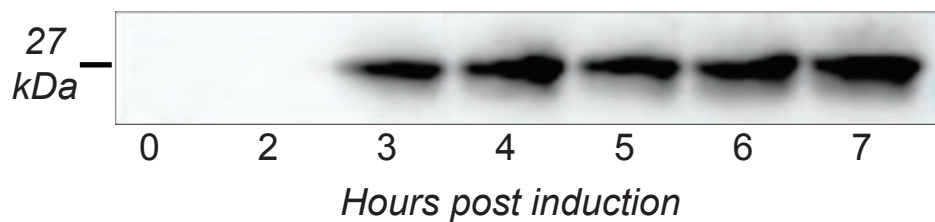

**A<sub>753</sub>**

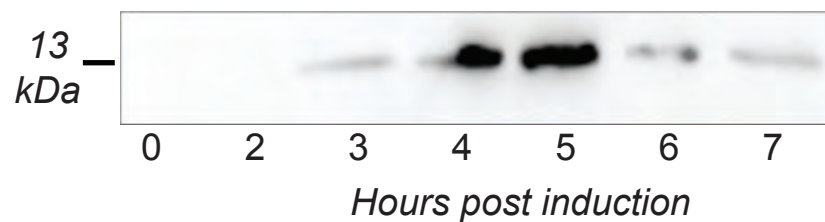

Supplement: S4 Fig — Immunoblot analysis of E. coli expressing heterologous candidate toxins and antitoxins cloned into the pBAD/His expression vector. Expression was induced by addition of arabinose and monitored during 7 hours. Total proteins was separated onto a 12% SDS PAGE before being transferred to a nitrocellulose membrane. Immunoblots were carried out using a commercially available anti-His antibody. (PDF) [file pgen.1009365.s004.pdf]

**A**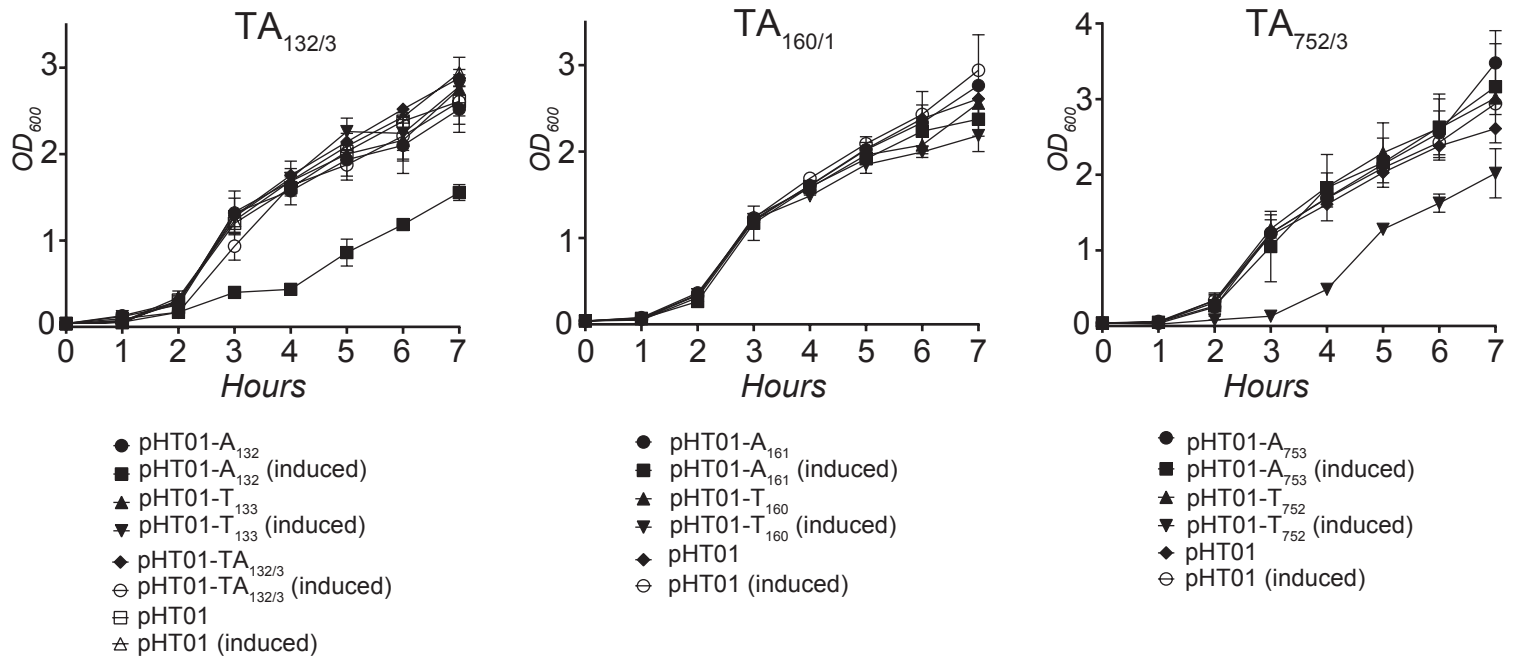**B**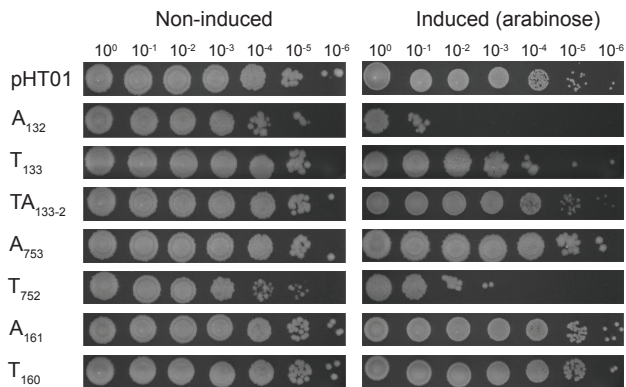**C**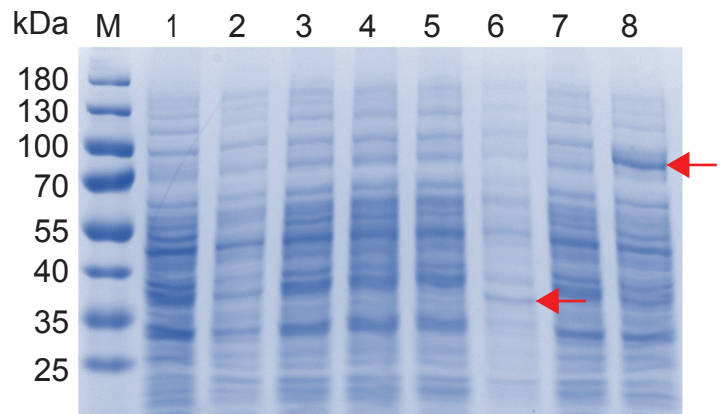**D**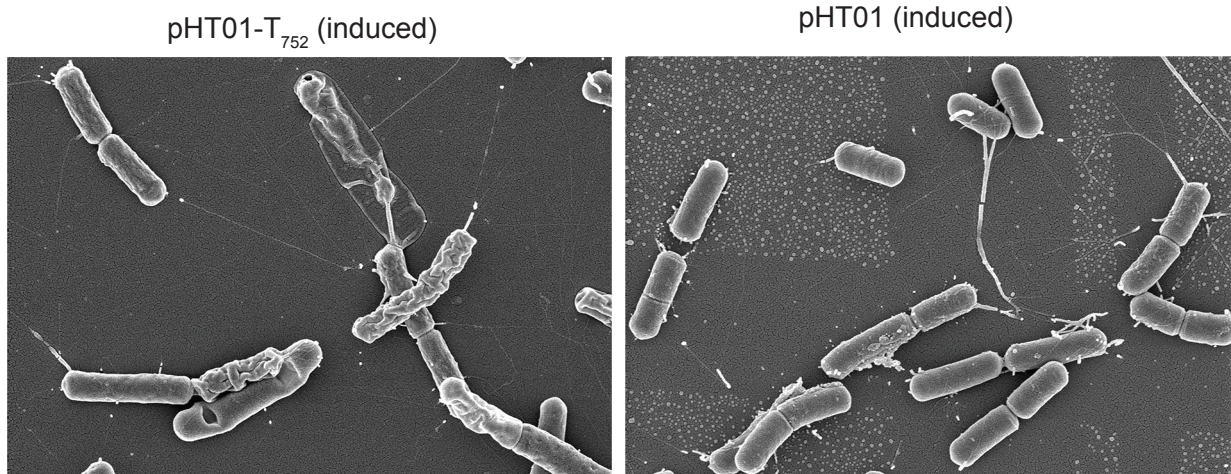

Supplement: S5 Fig — (A) Growth curves of B. subtilis harboring the different plasmid constructs of the TA132/3 (left), TA160/1 (middle) and TA752/3 (right) over a period of 7 hours after induction with arabinose (I) or non-induction (NI). Optical densities at 600nm were measured every hour and displayed using GraphPad Prism 9. Each data point represents the mean of three biological replicates, bars indicate standard deviation. (B) Ectopic expression of TA elements in B. subtilis, Total proteins of B. subtilis harboring the pHT01-T752 non-induced (lane 1), the pHT01-T752 induced (lane 2), the pHT01-A753 non-induced (lane 3), the pHT01-A753 induced (lane 4), the pHT01-A132 non-induced (lane 5), the pHT01-A132 induced (lane 6), the pHT01-T133 non-induced (lane 7), the pHT01-T133 induced (lane 8), were checked by SDS-PAGE and visualized using Coomassie staining. Arrows indicate the induction of the recombinant proteins at the expected sizes. (C) Toxicity neutralization spot assays in response to the repression (left) or induction (right) of the heterologous expression of the different toxins, antitoxins and entire TA systems. The empty vector pHT01 was used as negative control. (D) Scanning electron micrograph (magnification 10,000x) displaying morphological changes of B. subtilis observed 5 hours after the induction of T752 recombinant protein. The pHT01 empty vector was used a negative control. Indentations of cells are indicated by asterisk, empty/ shrunk cells by arrowhead and shrunk cells upon division by arrows. (PDF) [file pgen.1009365.s005.pdf]
