# Supplementary material for: Minimalistic mycoplasmas harbor different functional toxin-antitoxin systems
Source: PLoS Genet. 2021 Oct 21;17(10):e1009365. doi: 10.1371/journal.pgen.1009365 (PMC8562856; doi:10.1371/journal.pgen.1009365)
Supplement: S1 Table — (DOCX) [file pgen.1009365.s006.docx]

| **TA**  **system** | **Ensemble gene ID** | **Protein size in [AA]** | **Gene description from Ensembl** | **E-value** | **Predicted**  **Role in TA system** | **Operon structure** | **Pfam_**  **description** |
| --- | --- | --- | --- | --- | --- | --- | --- |
| 1 | MMCAP1_0096 | 194 | Xaa-Pro dipeptidase | 0.036 | A | AT | YmcE_antitoxin |
|  | MMCAP1_0097 | 303 | DNA polymerase I, 5'-3' exonuclease | - | T | AT | - |
| 2 | MMCAP1_0160 | 250 | abortive infection protein AbiGII | 1.2e-53 | T | AT | AbiEii |
|  | MMACP1_0161 | 198 | abortive infection protein AbiGI | 3.8e-49 | A | AT | AbiEi_4 |
| 3 | MMCAP1_0209 | 305 | N-acetylmuramic acid 6-phosphate etherase | 0.00011 | T | xT | PemK_toxin |
|  | MMCAP1_0210 | 565 | PTS system N-acetylmuramic acid-specific eiibc component | - | X | xT | - |
| 4 | MMCAP1_0302 | 216 | nitroreductase family protein | 0.0034 | A | xxA | BrnA_antitoxin |
|  | MMCAP1_0303 | 1482 | DNA polymerase III, alpha subunit, Gram-positive type | - | X | xxA | - |
| 5 | MMCAP1_0377 | 433 | Obg family GTPase CgtA | - | X | xxxTxxxx | - |
|  | MMCAP1_0378 | 245 | NAD+ synthetase | 0.0038 | T | xxxTxxxx | Cpta_toxin |
|  | MMCAP1_0379 | 92 | conserved hypothetical protein | - | X | xxxTxxxx | - |
| 6 | MMCAP1_0483 | 186 | holliday junction DNA helicase RuvA | 0.0005 | T | Txx | ParE_toxin |
|  | MMCAP1_0484 | 307 | holliday junction ATP-dependent DNA helicase RuvB | - | X | Txx | - |
| 7 | MMCAP1_0503 | 133 | conserved hypothetical protein | - | X | Tx | - |
|  | MMCAP1_0504 | 295 | conserved hypothetical protein | 0.0033 | T | Tx | HigB_toxin |
| 8 | MMCAP1_0513 | 110 | holo-[acyl-carrier-protein] synthase | 0.002 | A | XxxxxxA | HicB_lk_antitox |
|  | MMCAP1_0514 | 358 | conserved hypothetical protein | - | X | xxxxxxA | - |
| 9 | MMCAP1_0524 | 308 | S-adenosyl-methyltransferase MraW | - | X | Axx | - |
|  | MMCAP1_0525 | 133 | protein MraZ | 7e-26 | A | Axx | MraZ |
| 10 | MMCAP1_0610 | 274 | DNA-formamidopyrimidine glycosylase | - | X | xTx | - |
|  | MMCAP1_0611 | 911 | DNA polymerase I superfamily | - | T | xTx | - |
|  | MMCAP1_0612 | 987 | DNA polymerase III, alpha subunit subfamily, putative | - | X | xTx | - |
| 11 | MMCAP1_0616 | 279 | EDD domain protein, DegV family, putative | 0.031 | A | AA | CcdA |
|  | MMCAP1_0617 | 283 | DegV family protein | 0.00078 | A | AA | ParBc |
| 12 | MMCAP1_0625 | 310 | carbamate kinase 1 | 0.0039 | A | A | MazE_antitoxin |
| 13 | MMCAP1_0726 | 244 | glucosamine-6-phosphate deaminase | 0.0044 | T | TxxxxT | CbtA_toxin |
|  | MMCAP1_0727 | 248 | triose-phosphate isomerase | - | X | TxxxxT | - |
|  | MMCAP1_0730 | 199 | conserved hypothetical protein | - | X | TxxxxT | - |
|  | MMCAP1_0731 | 337 | conserved hypothetical protein | 3.8e-29 | T | TxxxxT | AbiEii |
| 14 | MMCAP1_0752 | 190 | cell filamentation protein (fic) | - | T | T | - |
|  | MMCAP1_0753 | 90 | DNA-damage-inducible protein J | 3.5e-9 | A | A | RelB |
| 15 | MMCAP1_0769 | 239 | GANTC--recognizing Type II restriction modification system (MmyCIV) adenine DNA methylase subunit | 5e-7 | A | xA | ParBc |
|  | MMCAP1_0770 | 281 | conserved hypothetical protein | - | X | xA | - |
| 16 | MMCAP1_0784 | 310 | carbamate kinase 1 | 0.0024 | A | xxA | MazE_antitoxin |
|  | MMCAP1_0785 | 510 | arginine/ornithine antiporter | - | X | xxA | - |
| 17 | MMCAP1_0882 | 214 | conserved hypothetical protein | - | X | Tx | - |
|  | MMCAP1_0883 | 326 | aspartate--ammonia ligase | 0.0084 | T | Tx | PIN |
| 18 | MMCAP1_0890 | 199 | conserved hypothetical protein | 2.6e-45 | A | A | AbiEi_4 |

A = antitoxin; T = toxin ; - = no E-value associated (guilt-by-association and/or only TA-like InterPro annotations); x = gene from same operon as where a T/A hit was found
