# Supplementary material for: Minimalistic mycoplasmas harbor different functional toxin-antitoxin systems
Source: PLoS Genet. 2021 Oct 21;17(10):e1009365. doi: 10.1371/journal.pgen.1009365 (PMC8562856; doi:10.1371/journal.pgen.1009365)
Supplement: S2 Table — (DOCX) [file pgen.1009365.s007.docx]

| **Strain name** | **No. of reads** | **Coverage (mean)** | **Reads N_50_ (bp)** | **GenBank accession no.** |
| --- | --- | --- | --- | --- |
| 152/93 | 134,693 | 353 X | 4,161 | CP068010.1 |
| 171/93 | 82,511 | 944 X | 10,488 | CP065586 |
| 7730 | 119,597 | 320 X | 4,615 | CP065584 |
| 80/93 | 111,196 | 331 X | 4,847 | CP065583 |
| 83/93 | 136,307 | 394 X | 4,696 | CP065582 |
| G1283.94 | 139,287 | 930 X | 10,085 | CP065580 |
| G1313.94 | 169,513 | 516 X | 5,428 | CP065579 |
| IVB-X | 103,564 | 315 X | 5,287 | CP065578 |
| M-18 | 136,870 | 381 X | 4,928 | CP065577 |
| M-5 | 67,246 | 239 X | 5,020 | CP065588 |
| My-325 | 117,700 | 356 X | 5,474 | CP065576 |
| My-I | 107,800 | 310 X | 5,595 | CP065575 |
| PG3 | 74,689 | 231 X | 5,353 | CP065581 |
| Wi8079 | 157,20 | 446 X | 4,571 | CP065574 |
