# Supplementary material for: Minimalistic mycoplasmas harbor different functional toxin-antitoxin systems
Source: PLoS Genet. 2021 Oct 21;17(10):e1009365. doi: 10.1371/journal.pgen.1009365 (PMC8562856; doi:10.1371/journal.pgen.1009365)
Supplement: S3 Table — (DOCX) [file pgen.1009365.s008.docx]

| No | Sequence 5’-3’ | Target | Use |
| --- | --- | --- | --- |
| 1 | cataatagatgataaacaagaag | cDNA *Mmc* GM12 | Forward primer for amplification of MMCAP2_0133 internal fragment on cDNA |
| 2 | cgttatcattagttcctgc | cDNA *Mmc* GM12 | Reverse primer for amplification of MMCAP2_0133 internal fragment on cDNA |
| 3 | aaaaggagttattaatgc | cDNA *Mmc* GM12 | Forward primer for amplification of MMCAP2_0132 internal fragment on cDNA |
| 4 | ttcagctatatctaacatatc | cDNA *Mmc* GM12 | Reverse primer for amplification of MMCAP2_0132 internal fragment on cDNA |
| 5 | cacttattagagttatgagat | cDNA *Mmc* GM12 | Forward primer for amplification of MMCAP2_0752 internal fragment on cDNA |
| 6 | cattgctaataagtactcatc | cDNA *Mmc* GM12 | Reverse primer for amplification of MMCAP2_0752 internal fragment on cDNA |
| 7 | tcaatattaaaatggatcctg | cDNA *Mmc* GM12 | Forward primer for amplification of MMCAP2_0753 internal fragment on cDNA |
| 8 | gctaaaagacgttcaccttc | cDNA *Mmc* GM12 | Reverse primer for amplification of MMCAP2_0753 internal fragment on cDNA |
| 9 | ttaattacagattccaccag | cDNA *Mmc* GM12 | Forward primer for amplification of MMCAP2_0160 internal fragment on cDNA |
| 10 | gcaattggtgatgttataac | cDNA *Mmc* GM12 | Reverse primer for amplification of MMCAP2_0160 internal fragment on cDNA |
| 11 | cttcataaactttcttacc | cDNA *Mmc* GM12 | Forward primer for amplification of MMCAP2_0161 internal fragment on cDNA |
| 12 | aacaactgataaaagaagcc | cDNA *Mmc* GM12 | Reverse primer for amplification of MMCAP2_0161 internal fragment on cDNA |
| 13 | TGATCGAGCTCAAGAAAGCGAACGTGCTG | MMCAP2_0132 | Forward primer amplifying gene A_132_, *Sac*I site is underlined |
| 14 | CGATCGAATTCCTCATTGCTATTCAGTTCTTTCACC | MMCAP2_0132 | Reverse primer amplifying gene A_132_, *Eco*RI site is underlined |
| 15 | TGATCGAGCTCAAGAAGGTTAATGTGAATATCAAAATGGAC | MMCAP2_0753 | Forward primer amplifying gene A_753_, *Sac*I site is underlined |
| 16 | CGATCGAATTCTTTGTCCAGGCTGTCCAGAATC | MMCAP2_0753 | Reverse primer amplifying gene A_753_ *Eco*RI site is underlined |
| 17 | ATGATCGAGCTCAACTATAAGGAGCAGC | MMCAP2_0161 | Forward primer amplifying gene A_161_, *Sac*I site is underlined |
| 18 | AAGTCTGAATTCTTAAACCAGCACTTGG | MMCAP2_0161 | Reverse primer amplifying gene A_161_, *Eco*RI site is underlined |
| 19 | ATGCCATAGCATTTTTATCC | pBAD/His | Forward primer for sequencing of pBAD constructs |
| 20 | GATTTAATCTGTATCAGG | pBAD/His | Reverse primer for sequencing of pBAD constructs |
| 21 | TAGCAATGAGTAATTATCAGACCGCTTCTGC | pBAD/T_133_ | Forward primer amplification of toxin T_133_ and AraC from pBAD, neutralization plasmid |
| 22 | CTCAGCTTCCTTTCGGGCTTTGTTATTATGACAACTTGACGGC | pBAD/T_133_ | Reverse primer amplification of toxin T_133_ and AraC from pBAD, neutralization plasmid |
| 23 | CGGCCTGGTGCCGCGCGGCAGCCATATGAAGAAAGCGAACGTG | pUC57_MMCAP2_0132 | Forward primer amplification of antitoxin A_132_, neutralization plasmid |
| 24 | GCGGTCTGATAATTACTCATTGCTATTCAGTTC | pUC57_MMCAP2_0132 | Reverse primer amplification of antitoxin A_132_, neutralization plasmid |
| 25 | TAACAAAGCCCGAAAGGAAGCTGAGTTGGC | pET28 | Forward primer pET28 amplification for neutralization plasmid |
| 26 | ATGGCTGCCGCGCGGCAC | pET28 | Reverse primer pET28 amplification for neutralization plasmid |
| 27 | AAAGAACGTGGACTCCAACG | pET28 | Forward primer junction pET28-toxin T_133_ |
| 28 | GTTAGGGATTAGCGTCTTAAGC | pBAD/T_133_ fragment | Reverse primer junction pET28-toxin T_133_ |
| 29 | CGATAACAAGATCGACATTCACG |  | Forward primer junction antitoxin A_132_ – toxin T_133_ |
| 30 | CCGACGATAAGCTGGACATC |  | Reverse primer junction antitoxin A_132_ – toxin T_133_ |
| 31 | GATTTCCTCGCTAATCACTTG |  | Forward primer junction antitoxin A_132_ – pET28 |
| 32 | ACTATGGATCCATGAACAACCTGATCG | pUC57 | Forward primer amplifying gene T_133_ for pHT01 construct, *Bam*HI site is underlined |
| 33 | ACTATCccgggTTATTCGAAGTCCAGG | pUC57 | Reverse primer amplifying gene T_133_ for pHT01 construct, *Xma*I site is underlined |
| 34 | ACTATGGATCCATGAAAGCGAACTTCC | pUC57 | Forward primer amplifying gene T_752_ for pHT01 construct, *Bam*HI site is underlined |
| 35 | ACTATCccgggTTATTTAATGTTGATCTTGAAGC | pUC57 | Reverse primer amplifying gene T_752_ for pHT01 construct, *Xma*I site is underlined |
| 36 | ACTATGGATCCATGATCAGCATTCTGGAGAA | pUC57 | Forward primer to amplify gene T_160_ from plasmid pUC57 to use for pHT01 construct, *Bam*HI site is underlined |
| 37 | ACTATCccgggTTAGCTGCTCTTCAGT | pUC57 | Reverse primer to amplifying gene T_160_ for pHT01 construct, *Xma*I site is underlined |
| 38 | ACTATGGATCCATGAACTATAAGGAGCAGC | pUC57 | Forward primer amplifying gene A_161_ for pHT01 construct, *Bam*HI site is underlined |
| 39 | ACTATCccgggTTAAACCAGCACTTGG | pUC57 | Reverse primer amplifying gene A_161_ for pHT01 construct, *Xma*I site is underlined |
| 40 | ATCTACGGATCCAAGAAAGCGAACGTGCTGAA | pUC57 | Forward primer amplifying gene A_132_ for pHT01 construct, *Bam*HI site is underlined |
| 41 | AGACTTTCTAGATTACTCATTGCTATTCAGTTC | pUC57 | Reverse primer amplifying gene A_132_ for pHT01 construct, *Xba*I site is underlined |
| 42 | aattaaaggaggaagGATCCATGAAGAAGGTTAATGTGAA | pUC57 | Forward primer amplifying gene A_753_ for pHT01 construct, overlap with pHT01 site is underlined |
| 43 | attaggcgggctgccccggGTTATTTGTCCAGGCTGTCCA | pUC57 | Reverse primer amplifying gene A_753_ for pHT01 construct, overlap with pHT01 site is underlined |
| 44 | TGGACAGCCTGGACAAATAACccggggcagcccgcctaat | pHT01 | Forward primer amplifying pHT01 construct, overlap with A_753_ site is underlined |
| 45 | TTCACATTAACCTTCTTCATGGATCcttcctcctttaatt | pHT01 | Reverse primer amplifying pHT01 construct, overlap with A_753_ site is underlined |
| 46 | gtacgtacgatctttcagc | pHT01 | Forward primer pHT01 constructs PCR and sequencing |
| 47 | CAGTTGCAGACAAAGATCTC | pHT01 | Reverse primer pHT01 constructs PCR and sequencing |
| 48 | GATGATCGGGTTGATATCG | T_133_ | Reverse verification primer amplifying pHT01- T_133_ junction |
| 49 | CTGAAACAGGAACTTGTTG | A_132_ | Reverse verification primer amplifying pHT01- A_132_ junction |
| 50 | CTTGAACATGTAGCTGTGG | T_752_ | Reverse verification primer amplifying pHT01- T_752_ junction |
| 51 | TTTGTCCAGGCTGTCC | A_753_ | Reverse verification primer amplifying pHT01- A_753_ junction |
| 52 | GCTGGTTAATCTCGTTCAG | T_160_ | Reverse verification primer amplifying pHT01- T_160_ junction |
| 53 | TGTACATGGTCTTCGC | A_161_ | Reverse verification primer amplifying pHT01- A_161_ junction |
| 54 | CGTTGGTCGGGTCC | T_133_ | Reverse primer for sequencing |
| 55 | AGCGTTGACGATCTG | T_133_ | Forward primer for sequencing |
| 56 | CTTGCATTCGTTGTCG | T_133_ | Reverse primer for sequencing |
| 57 | ATGTTTTTCAGGTCACC | A_132_ | Reverse primer for sequencing |
| 58 | AGTACCTGGACTTCGAATAActctagagtcgacgtccccg | pHT01 | Forward primer amplifying pHT01 backbone, overlap with T_133_ site is underlined resulting in neutralization plasmid |
| 59 | AGCACGTTCGCTTTCTTCATTgatccttcctcctttaatt | pHT01 | Reverse primer amplifying pHT01 backbone, overlap with A_132_ site is underlined resulting in neutralization plasmid |
| 60 | aattaaaggaggaaggatcAATGAAGAAAGCGAACGTGCT | A_132_ | Forward primer amplifying A_132_ sequence, overlap with pHT01 site is underlined resulting in neutralization plasmid |
| 61 | tggtaccaagctaattccggTTACTCATTGCTATTCAGTT | A_132_ | Reverse primer amplifying A_132_ sequence, overlap with pHT01 site is underlined resulting in neutralization plasmid |
| 62 | AACTGAATAGCAATGAGTAAccggaattagcttggtacca | pHT01 | Forward primer amplifying Pgrac01 promoter region for T_133_, overlap with A_132_ is underlined (neutralization plasmid) |
| 63 | GCACGATCAGGTTGTTCATTgatccttcctcctttaattg | pHT01 | Reverse primer amplifying Pgrac01 promoter region for T_133_, overlap with T_133_ is underlined (neutralization plasmid) |
| 64 | aattaaaggaggaaggatcAATGAACAACCTGATCGTGCT | T_133_ | Forward primer amplifying T_133_ sequence, overlap with pHT01 site is underlined resulting in neutralization plasmid |
| 65 | cggggacgtcgactctagagTTATTCGAAGTCCAGGTACT | T_133_ | Reverse primer amplifying T_133_ sequence, overlap with pHT01 site is underlined resulting in neutralization plasmid |
| 66 | TCAAGATCAACATTAAATAActctagagtcgacgtccccg | pHT01 | Forward primer amplifying pHT01 backbone, overlap with T_752_ site is underlined resulting in neutralization plasmid |
| 67 | TCACATTAACCTTCTTCATTgatccttcctcctttaattg | pHT01 | Reverse primer amplifying pHT01 backbone, overlap with A_753_ site is underlined resulting in neutralization plasmid |
| 68 | caattaaaggaggaaggatcAATGAAGAAGGTTAATGTGA | A_753_ | Forward primer amplifying A_753_ sequence, overlap with pHT01 site is underlined resulting in neutralization plasmid |
| 69 | tggtaccaagctaattccggTTATTTGTCCAGGCTGTCCA | A_753_ | Reverse primer amplifying A_753_ sequence, overlap with pHT01 site is underlined resulting in neutralization plasmid |
| 70 | TGGACAGCCTGGACAAATAAccggaattagcttggtacca | pHT01 | Forward primer amplifying Pgrac01 promoter region for T_752_, overlap with A_753_ is underlined (neutralization plasmid) |
| 71 | CCAGGAAGTTCGCTTTCATTgatccttcctcctttaattg | pHT01 | Reverse primer amplifying Pgrac01 promoter region for T_752_, overlap with T_752_ is underlined (neutralization plasmid) |
| 72 | caattaaaggaggaaggatcAATGAAAGCGAACTTCCTGG | T_752_ | Forward primer amplifying T_752_ sequence, overlap with pHT01 site is underlined resulting in neutralization plasmid |
| 73 | cggggacgtcgactctagagTTATTTAATGTTGATCTTGA | T_752_ | Reverse primer amplifying T_752_ sequence, overlap with pHT01 site is underlined resulting in neutralization plasmid |
| 74 | TGAAGCTGCTGTAGCGTTT | A_753_ | Reverse primer for PCR verifying neutralization plasmid pHT01/TAS_752/3_ |
| 75 | ATTAGCGAGGAAATCAAG | A_132_ | Forward primer sequencing neutralization plasmid pHT01/TAS_133/2_ |
| 76 | GAAAGTGGATAACATCAAGAG | A_132_ | Forward primer sequencing neutralization plasmid pHT01/TAS_133/2_ |
| 77 | CGTTGGTCGGGTCC | T_133_ | Reverse primer sequencing neutralization plasmid pHT01/TAS_133/2_ |
| 78 | ATGTTTGCGAAGTGGAAC | T_133_ | Forward primer sequencing neutralization plasmid pHT01/TAS_133/2_ |
| 79 | GCCCTATAGTGAGTCGTATTAC | pMYCO1 | Forward primer amplifying the pMYCO1 backbone of all constructs |
| 80 | GAATTCGAGCTCGGTACC | pMYCO1 | Reverse primer for Gibson assembly, for pMYCO1 backbone of all constructs |
| 81 | CGGGTACCGAGCTCGAATTCTTAATATAATTAATTTAAAAGTTGCTTTTTATAATAAG | MMCAP2_0132 | Forward primer for construction of pMYCO1-pNat1-A_132_/T_133_, MYCO1-pNat1-A_132_ and pMYCO1-pNat1-T_133_ |
| 82 | GATTATTCATGATTTTAACTCTTTTCAAAGTTATTATATTTC | MMCAP2_0133 | Reverse primer for construction of pMYCO1-pNat1-T_133_ |
| 83 | AATATAAGAAATGAATAATCTAATTGTATTAAAAGGAAAG | MMCAP2_0133 | Forward primer for construction of pMYCO1-pNat1-T_133_ |
| 84 | AATACGACTCACTATAGGGCCTATTCAAAATCTAAATATTCATTTGAC | MMCAP2_0133 | Reverse primer for construction of pMYCO1-pNat1-A_132_/T_133,_ pMYCO1-pNat1-T_133_ and pMYCO1-pSpi-T_133_ |
| 85 | AATACGACTCACTATAGGGCTTATTCATTTGAATTTAACTCCTTC | MMCAP2_0132 | Reverse primer for construction of pMYCO1-pNat1-A_132_ |
| 86 | CGGGTACCGAGCTCGAATTCGAATTAAAAGTTAGTGAACAAGAAAAC | Spiralin promoter | Forward primer for Gibson assembly, fragments with Spiralin promotor |
| 87 | AGATTATTCATTTCTTATATTTCCTTTCTCTATTAAGTAG | MMCAP2_0133 | Reverse primer for construction of pMYCO1-pSpi-T_133_ |
| 88 | AATATAAGAAATGAATAATCTAATTGTATTAAAAGGAAAG | MMCAP2_0133 | Forward primer for construction of pMYCO1-pSpi-T_133_ |
| 89 | CGGGTACCGAGCTCGAATTCATAACCATCCTTTTTCTATGTTTTAC | MMCAP2_0752 | Forward primer for construction of pMYCO1-pNat3-T_752_-pNat4-A_753_ and pMYCO1-pNat3-T_752_ |
| 90 | AATACGACTCACTATAGGGCTTATTTATCTAATGAATCTAATATTTCTTTAAAAC | MMCAP2_0753 | Reverse primer for construction of pMYCO1-pNat3-T_752_-pNat4-A_753_ and pMYCO1-pNat4-A_753_ |
| 91 | AATACGACTCACTATAGGGCTTATTTAATATTTATTTTAAAACCTTCATACTC | MMCAP2_0752 | Reverse primer for construction of pMYCO1-pNat3-T_752_ and pMYCO1-pSpi-T_752_ |
| 92 | CGGGTACCGAGCTCGAATTCACTAGGTATTTTTAAAGTTCTTAGAG | MMCAP2_0753 | Forward primer for construction of pMYCO1-pNat4**-**A_753_ |
| 93 | TAGCTTTCATTTCTTATATTTCCTTTCTCTATTAAGTAG | MMCAP2_0752 | Reverse primer for construction of pMYCO1-pSpi**-**T_752_ |
| 94 | AATATAAGAAATGAAAGCTAATTTTTTAGAAGAAG | MMCAP2_0752 | Forward primer for construction of pMYCO1-pSpi**-**T_752_ |
| 95 | CGGGTACCGAGCTCGAATTCAAAATATCTTAAATAACCATAAGTTAAAATTATTTG | MMCAP2_0161 | Forward primer for construction of pMYCO1-pNat2-A_161_/T_160_, pMYCO1-pNat2-A_161_ and pMYCP1-pNat2-T_160_ |
| 96 | TGGATATCATAATTATCACCTATGTTCTTATTTTTC | MMCAP2_0160 | Reverse primer for construction of pMYCO1-pNat2-T_160_ |
| 97 | GGTGATAATTATGATATCCATATTAGAAAAAATTGTTAAAAG | MMCAP2_0160 | Forward primer for construction of pMYCO1-pNat2-T_160_ |
| 98 | AATACGACTCACTATAGGGCCTAAGAGCTTTTTAACTTTTCTAAAAG | MMCAP2_0160 | Reverse primer for construction of pMYCO1-pNat2-A_161_/T_160_, pMYCO1-pNat2-T_160_ and pMYCP1-pSpi-T_160_ |
| 99 | AATACGACTCACTATAGGGCTTAAACTAATACTTGAAAAACTTCATAAAC | MMCAP2_0161 | Reverse primer for construction of pMYCO1-pNat2-A_161_ |
| 100 | TGGATATCATTTCTTATATTTCCTTTCTCTATTAAGTAG | MMCAP2_0160 | Reverse primer for construction of pMYCO1-pSpi-T_160_ |
| 101 | AATATAAGAAATGATATCCATATTAGAAAAAATTGTTAAAAG | MMCAP2_0160 | Forward primer for construction of pMYCO1-pSpi-T_160_ |
| 102 | TGGAAGTGGTAAAGGCTCAC | MMCAP2_0132 | Reverse primer for Gibson assembly fragment insertion verification |
| 103 | CAAATAACTTACTCTGTTAC | MMCAP2_0133 | Reverse primer for Gibson assembly fragment insertion verification |
| 104 | ATATCTTGAGTGGTTCTTTC | MMCAP2_0160 | Reverse primer for Gibson assembly fragment insertion verification |
| 105 | ATAATCAGGATCATAGTGCC | MMCAP2_0161 | Reverse primer for Gibson assembly fragment insertion verification |
| 106 | AGATCTGCCATTACCTTCTC | MMCAP2_0752 | Reverse primer for Gibson assembly fragment insertion verification |
| 107 | GTCGTTTTGTTCAACAGCAG | MMCAP2_0753 | Reverse primer for Gibson assembly fragment insertion verification |
| 108 | GACAATAAGTGATGACTTGC | MMCAP2_0133 | Reverse primer for verification of toxin T_133_ sequence presence in pMYCO1 after transformation into *Mcap*, primer used for sequencing of T_133_ amplification |
| 109 | GGAGGTAGTGGTATGAAAAG | pMYCO1 | Forward primer for verification of toxin and antitoxin sequence presence in pMYCO1, primer used for sequencing of T_133_ amplification |
| 110 | CAGTGTTATCACTCATGGTTATGG | pMYCO1 | Reverse primer for verification of toxin and antitoxin sequence presence in pMYCO1 after transformation into *Mcap* |
| 111 | ATTTGCCTTTTTCATGATTTTAACTCTTTTCAAAGTTATTATATTTCAC | pMYCO1-pNat1-A_132_/T_133_ | Reverse primer for the construction pMYCO1-pMmcNat-D500_TA_0458/9_ and pMYCO1-pMmcNat-D500_T_0458_ |
| 112 | GAAGTTGATTTTGAATAGGCCCTATAGTGAGTCGTATTAC | pMYCO1-pNat1-A_132_/T_133_ | Forward primer for the construction of pMYCO1-pMmcNat-D500_TA_0458/9_ and pMYCO1-pMmcNat-D500_T_0458_ |
| 113 | AAAAGAGTTAAAATCATGAAAAAGGCAAATGTTATAAAC | D500_0458 | Forward primer for the construction of pMYCO1-pMmcNat-D500_TA_0458/9_ |
| 114 | CGACTCACTATAGGGCCTATTCAAAATCAACTTCGTTTTGAATTG | D500_0459 | Reverse primer for the construction of pMYCO1-pMmcNat-D500_TA_0458/9_ |
| 115 | GATTTTATTTGGCATGATTTTAACTCTTTTCAAAGTTATTATATTTCAC | pMYCO1-pNat1-A_132_/T_133_ | Reverse primer for the construction of pMYCO1-pMmcNat-D500_T_0458_ |
| 116 | AAAAGAGTTAAAATCATGCCAAATAAAATCGTAGAATTAAAAAAAG | D500_0458 | Forward primer for the construction of pMYCO1-pMmcNat-D500_T_0458_ |
| 117 | ATTAGCTTTTTTCATGATTTTAACTCTTTTCAAAGTTATTATATTTCAC | pMYCO1-pNat1-A_132_/T_133_ | Reverse primer for the construction of pMYCO1-pMmcNat-BOVPG45_TA_0623/4_ |
| 118 | ATAGAATTTAATTCTTAAGCCCTATAGTGAGTCGTATTAC | pMYCO1-pNat1-A_132_/T_133_ | Forward primer for the construction of pMYCO1-pMmcNat-BOVPG45_TA_0623/4_ and pMYCO1-pMmcNat-BOVPG45_T_0623_ |
| 119 | AAAAGAGTTAAAATCATGAAAAAAGCTAATATATTAAATTTAATACAATACC | MBOVPG45_0624 | Forward primer for the construction of pMYCO1-pMmcNat-BOVPG45_TA_0623/4_ and pMYCO1-pMmcNat-BOVPG45_T_0623_ |
| 120 | GACTCACTATAGGGCTTAAGAATTAAATTCTATTTGTTCATTTGATTTAG | MBOVPG45_0623 | Reverse primer for the construction of pMYCO1-pMmcNat-BOVPG45_TA_0623/4_ |
| 121 | TAATAATGAATTCATGATTTTAACTCTTTTCAAAGTTATTATATTTCAC | pMYCO1-pNat1-A_132_/T_133_ | Reverse primer for the construction of pMYCO1-pMmcNat-BOVPG45_T_0623_ |
| 122 | AAAAGAGTTAAAATCATGAATTCATTATTAGTTTTAAAAGGGGAATTTG | MBOVPG45_0623 | Forward primer for the construction of pMYCO1-pMmcNat-BOVPG45_T_0623_ |
| 123 | CGTTTCTTTTTTCATGATTTTAACTCTTTTCAAAGTTATTATATTTCAC | pMYCO1-pNat1-A_132_/T_133_ | Reverse primer for the construction of pMYCO1-pMmcNat-GALPG31_Toxin/Antitoxin |
| 124 | GATATTAATTTTGAATAAGCCCTATAGTGAGTCGTATTAC | pMYCO1-pNat1-A_132_/T_133_ | Forward primer for the construction of pMYCO1-pMmcNat-GALPG31_Toxin/Antitoxin and pMYCO1-pMmcNat-GALPG31_Toxin |
| 125 | AAAAGAGTTAAAATCATGAAAAAAGAAACGGTTATAAATTTAATTAGG | *Mycoplasma gallisepticum* Antitoxin | Forward primer for the construction of pMYCO1-pMmcNat-GALPG31_Toxin/Antitoxin |
| 126 | ACGACTCACTATAGGGCTTATTCAAAATTAATATCTTCTTTAGCTTTTTC | *Mycoplasma gallisepticum* Toxin | Reverse primer for the construction of pMYCO1-pMmcNat-GALPG31_Toxin/Antitoxin and pMYCO1-pMmcNat-GALPG31_Toxin |
| 127 | TAATATTTCATTCATGATTTTAACTCTTTTCAAAGTTATTATATTTCAC | *Mycoplasma gallisepticum* Toxin | Reverse primer for the construction of pMYCO1-pMmcNat-GALPG31_Toxin |
| 128 | AAAAGAGTTAAAATCATGAATGAAATATTAAAATTAAGAGTTAATTTCTC | *Mycoplasma gallisepticum* Toxin | Forward primer for the construction of pMYCO1-pMmcNat-GALPG31_Toxin |
| 129 | TATAAGATCCTTTGAATGGAGAAAAAAATCACTGGATATACCACC | pCC1BAC-His3 | Reverse primer for the construction of pMYCO1-ChloR |
| 130 | ACAACTTAAATTACATTACGCCCCGCCCTGCCACTC | pCC1BAC-His3 | Reverse primer for the construction of pMYCO1-ChloR |
| 131 | CAGGGCGGGGCGTAATGTAATTTAAGTTGTTATATAAAGATCTGAACTGC | pMYCO1 | Forward primer for the construction of pMYCO1-ChloR |
| 132 | GATTTTTTTCTCCATTCAAAGGATCTTATATTTCCTTTCTCTATTAAG | pMYCO1 | Reverse primer for the construction of pMYCO1-ChloR |
| 133 | ATCCCCGGGTACCGAGCTCGAATTCTGAAAAAACTTTTATCGATCAAAAAC | pXyl-tetO2 synthetic construct | Forward primer for construction of pMYCO1-Chlo^R^-pXyl/tetO_2_ |
| 134 | AGTAGTTCACCACCTTTTC | pXyl-tetO2 synthetic construct | Reverse primer for construction of pMYCO1-Chlo^R^-pXyl/tetO_2_ |
| 135 | GCCCTATAGTGAGTCGTATTAC | pMYCO1-ChloR | Forward primer for construction of pMYCO1-Chlo^R^-pXyl/tetO_2_ |
| 136 | GAATTCGAGCTCGGTACC | pMYCO1-ChloR | Reverse primer for construction of pMYCO1-Chlo^R^-pXyl/tetO_2_ |
| 137 | AAATTAAAGTTGGTTCATTCAAAG | pXyl-tetO2 synthetic construct | Forward primer for construction of pMYCO1-Chlo^R^-pXyl/tetO_2_ |
| 138 | ATTGTAATACGACTCACTATAGGGCAATTAAAAGTTAGTGAACAAGAAAAC | pXyl-tetO2 synthetic construct | Reverse primer for construction of pMYCO1-Chlo^R^-pXyl/tetO_2_ |
| 139 | ATATAGGGAAAAGGTGGTGAACTACTATGAATAATCTAATTGTATTAAAAGGAAAGTTTG | MMCAP2_0133 | Forward primer for construction of pMYCO1-chloR-pXyltetO2-TetR-MMCAP2_0133 |
| 140 | ACTTTGAATGAACCAACTTTAATTTCTATTCAAAATCTAAATATTCATTTGAC | MMCAP2_0133 | Reverse primer for construction of pMYCO1-chloR-pXyltetO2-TetR-MMCAP2_0133 |
| 141 | TATAGGGAAAAGGTGGTGAACTACTATGATATCCATATTAGAAAAAATTGTTAAAAG | MMCAP2_0160 | Forward primer for construction of pMYCO1-chloR-pXyltetO2-TetR-MMCAP2_0160 |
| 142 | ACTTTGAATGAACCAACTTTAATTTCTAAGAGCTTTTTAACTTTTCTAAAAG | MMCAP2_0160 | Reverse primer for construction of pMYCO1-chloR-pXyltetO2-TetR-MMCAP2_0160 |
| 143 | TATATAGGGAAAAGGTGGTGAACTACTATGAAAGCTAATTTTTTAGAAGAAGAATTTGAG | MMCAP2_0752 | Forward primer for the construction of pMYCO1-chloR-pXyltetO2-TetR-MMCAP2_0752 |
| 144 | ACTTTGAATGAACCAACTTTAATTTTTATTTAATATTTATTTTAAAACCTTCATACTC | MMCAP2_0752 | Reverse primer for the construction of pMYCO1-chloR-pXyltetO2-TetR-MMCAP2_0752 |
| 145 | ATCATCATCACTAAATTTAGCACC | *Mycoplasma gallisepticum* Toxin | Reverse primer for Gibson assembly fragment insertion verification and sequencing |
| 146 | ACATAGAACTAGTGATACAATACG | D500_0458 | Reverse primer for Gibson assembly fragment insertion verification and sequencing |
| 147 | GTGACAAATCTACAACTATAGAGC | MBOVPG45_0623 | Reverse primer for Gibson assembly fragment insertion verification and sequencing |
| 148 | TTACCAGTTCCTGGAGGACC | MBOVPG45_0624 | Reverse primer for Gibson assembly fragment insertion verification and sequencing |
| 149 | GTATTTGTATCAGAAAGAATGCTGG | D500_0459 | Reverse primer for Gibson assembly fragment insertion verification and sequencing |
| 150 | ACTAAGACCGTCAGATAATAAAGC | *Mycoplasma gallisepticum* Antitoxin | Reverse primer for Gibson assembly fragment insertion verification and sequencing |
| 151 | TGAAGCACCAGTTTCTGAACC | pMYCO1 | Reverse primer for Gibson assembly fragment insertion verification and sequencing |
| 152 | AAGCAGCTAAAGTAATTGAGG | pMYCO1 | Reverse primer for Gibson assembly fragment insertion verification and sequencing |
| 153 | TGTAAAACGACGGCCAGTGAATTG | pMYCO1 | Reverse primer for Gibson assembly fragment insertion verification and sequencing |
| 154 | ATCAAAGAGTTGTTGGTTGC | D500_0458 | Forward primer for sequence verification |
| 155 | AGAGTTAGACATTTTGGTGTAGC | D500_0458 | Forward primer for sequence verification |
| 156 | TGCTAAGCATCCATTTGTTGC | D500_0458 | Forward primer for sequence verification |
| 157 | ACTAGACCAAATTTCAAATCACC | MBOVPG45_0623 | Forward primer for sequence verification |
| 158 | ATTGCGGTAGATTTAGAGTACG | MBOVPG45_0623 | Forward primer for sequence verification |
| 159 | TGCTTTTCCATTTACCACAAAGG | MBOVPG45_0623 | Forward primer for sequence verification |
| 160 | ACACATAATCACCCATTGTGTCG | *Mycoplasma gallisepticum* Toxin | Forward primer for sequence verification |
| 161 | AGATTACTTTCATGGAACAGC | *Mycoplasma gallisepticum* Toxin | Forward primer for sequence verification |
| 162 | AGCATTGATTATTGATTCAGC | *Mycoplasma gallisepticum* Toxin | Forward primer for sequence verification |
