# Supplementary material for: Minimalistic mycoplasmas harbor different functional toxin-antitoxin systems
Source: PLoS Genet. 2021 Oct 21;17(10):e1009365. doi: 10.1371/journal.pgen.1009365 (PMC8562856; doi:10.1371/journal.pgen.1009365)
Supplement: S4 Table — (DOCX) [file pgen.1009365.s009.docx]

| **Vector** | **Reference** | **Study** |
| --- | --- | --- |
| pBAD/His | ThermoFisher Scientific | Functionality of TAS in *Escherichia coli* LMG194 and MG1655 |
| pBAD-MazF | This study |  |
| pBAD-A_132_ | This study |  |
| pBAD-T_133_ | This study |  |
| pBAD-A_753_ | This study |  |
| pBAD-T_752_ | This study |  |
| pBAD-A_161_ | This study |  |
| pBAD-T_160_ | This study |  |
|  |  |  |
| pET28a | Novagen | Functionality of TAS in *Escherichia coli* LMG194 and MG1655 |
| pET28a-0132-pAra0133 | This study |  |
|  |  |  |
| pMYCO1 | [1] | Functionality of TAS in *Mycoplasma capricolum capricolum* ΔRE |
| pMYCO1-pNat1-T_133_ | This study |  |
| MYCO1-pNat1-A_132_ | This study |  |
| pMYCO1-pNat1-A_132_/T_133_ | This study |  |
| pMYCO1-pSpi-T_133_ | This study |  |
| pMYCO1-pNat3-T_752_ | This study |  |
| pMYCO1-pNat4-A_753_ | This study |  |
| pMYCO1-pNat3-T_752_-pNat4-A_753_ | This study |  |
| pMYCO1-pSpi-T_752_ | This study |  |
| pMYCP1-pNat2-T_160_ | This study |  |
| pMYCO1-pNat2-A_161_ | This study |  |
| pMYCO1-pNat2-A_161_/T_160_ | This study |  |
| pMYCP1-pSpi-T_160_ | This study |  |
| pMYCO1-pMmcNat-D500_TA_0458/9_ | This study |  |
| pMYCO1-pMmcNat-D500_T_0458_ | This study |  |
| pMYCO1-pMmcNat-BOVPG45_TA_0623/4_ | This study |  |
| pMYCO1-pMmcNat-BOVPG45_T_0623_ | This study |  |
| pMYCO1-pMmcNat-GALPG31_Toxin/Antitoxin | This study |  |
| pMYCO1-pMmcNat-GALPG31_Toxin | This study |  |
| pMYCO1-Chlo^R^ | This study | Inducible expression system in *Mycoplasma capricolum capricolum* ΔRE |
| pMYCO1-Chlo^R^-pXyl/tetO_2_ | This study |  |
| pMYCO1- Chlo^R^-pXyltetO2-TetR-MMCAP2_0133 | This study |  |
| pMYCO1- Chlo^R^-pXyltetO2-TetR-MMCAP2_0160 | This study |  |
| pMYCO1- Chlo^R^-pXyltetO2-TetR-MMCAP2_0752 | This study |  |
|  |  |  |
| pMT85tetM-PSlacZ-pRS313 | [2] |  |
|  |  |  |
| pXyl-tetO2 synthetic construct | GenScript |  |
|  |  |  |
| pCC1BAC-His3 | [3] |  |
|  |  |  |
| pHT01 | MoBiTec GmbH | Functionality of TAS in *Bacillus subtilis* Marburg |
| pHT01-A_0132_ | This study |  |
| pHT01-T_0133_ | This study |  |
| pHT01-A_0132_/T_0133_ | This study |  |
| pHT01-T_0752_ | This study |  |
| pHT01-A_0753_ | This study |  |
| pHT01-T_0160_ | This study |  |
| pHT01-A_0161_ | This study |  |

**References**

1. Lartigue C, Blanchard A, Renaudin J, Thiaucourt F, Sirand-Pugnet P. Host specificity of mollicutes oriC plasmids: functional analysis of replication origin. Nucleic Acids Res. 2003;31: 6610–8.

2. Labroussaa F, Lebaudy A, Baby V, Gourgues G, Matteau D, Vashee S, et al. Impact of donor-recipient phylogenetic distance on bacterial genome transplantation. Nucleic Acids Res. 2016;44: 8501–11. doi:10.1093/nar/gkw688

3. Gibson DG, Glass JI, Lartigue C, Noskov VN, Chuang R-Y, Algire MA, et al. Creation of a bacterial cell controlled by a chemically synthesized genome. Science. 2010;329: 52–56. doi:10.1126/SCIENCE.1190719
