# Supplementary material for: Minimalistic mycoplasmas harbor different functional toxin-antitoxin systems
Source: PLoS Genet. 2021 Oct 21;17(10):e1009365. doi: 10.1371/journal.pgen.1009365 (PMC8562856; doi:10.1371/journal.pgen.1009365)
Supplement: S4 File — (DOCX) [file pgen.1009365.s013.docx]

>*M. leachii* PG50 (NR_044773.1)(MSB_A0392)

AAAATGAGAGTTTGATCCTGGCTCAGGATAAACGCTGGCGGCATGCCTAATACATGCAAGTTGAACGGGGGTGCTTGCACCTCAGTGGCGAACGGGTGAGTAACACGTATCTAACCTACCTTATAGCGGGGGATAACTTTTGGAAACGAAAGATAATACCGCATGTAGATCTTATTATCGCATGAGAAAAGATCAAAAGAACCGTTTGGTTCACTATGAGATGGGGATGCGGCGTATTAGCTAGTAGGTGAGATAATAGCCCACCTAGGCGATGATACGTAGCCGAACTGAGAGGTTGATCGGCCACATTGGGACTGAGATACGGCCCAGACTCCTACGGGAGGCAGCAGTAGGGAATTTTTCACAATGGACGAAAGTCTGATGAAGCAATGCCGCGTGAGTGATGACGGCCTTCGGGTTGTAAAGCTCTGTTGTAAGGGAAGAAAAAATAAAGTAGGAAATGACTTTATCTTGACAGTACCTTACCAGAAAGCCACGGCTAACTATGTGCCAGCAGCCGCGGTAATACATAGGTGGCAAGCGTTATCCGGATTTATTGGGCGTATAGGGTGCGTAGGCGGTTTTGCAAGTTTGAGGTTAAAGTCCGGAGCTCAACTCCGGTTCGCCTTGAAAACTGTTTTACTAGAATGCAAGAGAGGTAAGCGGAATTCCATGTGTAGCGGTGAAATGCGTAGATATATGGAAGAACACCTGTGGCGAAAGCGGCTTACTGGCTTGTTATTGACGCTGAGGCACGAAAGCGTGGGGAGCAAATAGGATTAGATACCCTAGTAGTCCACGCCGTAAACGATGAGTACTAAGTGTTGGGGTAACTCAGCGCTGCAGCTAACGCATTAAGTACTCCGCCTGAGTAGTATGCTCGCAAGAGTGAAACTCAAAGGAATTGACGGGGACCCGCACAAGTGGTGGAGCATGTGGTTTAATTCGAAGCAACACGAAGAACCTTACCAGGGCTTGACATCCAGTGCAAAGCTATAGAGATATAGTAGAGGTTAACATTGAGACAGGTGGTGCATGGTTGTCGTCAGTTCGTGCCGTGAGGTGTTGGGTTAAGTCCCGCAACGAACGCAACCCTTGTCGTTAGTTACTAACATTAAGTTGAGAACTCTAACGAGACTGCTAGTGTAAGCTAGAGGAAGGTGGGGATGACGTCAAATCATCATGCCCCTTATGTCCTGGGCTACACACGTGCTACAATGGCTGGTACAAAGAGTTGCAATCCTGTGAAGGGGAGCTAATCTCAAAAAACCAGTCTCAGTTCGGATTGAAGTCTGCAACTCGACTTCATGAAGCCGGAATCACTAGTAATCGCGAATCAGCTATGTCGCGGTGAATACGTTCTCGGGTCTTGTACACACCGCCCGTCACACCATGAGAGTTGGTAATACCAGAAGTAGGTAGCTTAACCATTTGGAGAGCGCTTCCCAAGGTAGGACTAGCGATTGGGGTGAAGTCGTAACAAGGTATCCGTACGGGAACGTGCGGATGGATCACCTCCTTTCT

>*M. mycoides* subsp. *mycoides* PG1 (NR_074703.1)

TAAAATGAGAGTTTGATCCTGGCTCAGGATAAACGCTGGCGGCATGCCTAATACATGCAAGTCGAACGGAGGTGCTTGCACCTCAGTGGCGAACGGGTGAGTAACACGTATCTAACCTACCTCATAGCGGGGGATAACTTTTGGAAACGAAAGATAATACCGCATGTAGATCTTATTATCGCATGAGAAAAGATCAAAAGAACCGTTTGGTTCACTATGAGATGGGGATGCGGCGTATTAGCTAGTAGGTGAGATAATAGCCCACCTAGGCGATGATACGTAGCCGAACTGAGAGGTTGATCGGCCACATTGGGACTGAGATACGGCCCAGACTCCTACGGGAGGCAGCAGTAGGGAATTTTTCACAATGGACGAAAGTCTGATGAAGCAATGCCGCGTGAGTGATGACGGCCTTCGGGTTGTAAATCTCTGTTGTAAGGGAAGAAAAAATAAAGTAGGAAATGACTTTATCTTGACAGTACCTTACCAGAAAGCCACGGCTAACTATGTGCCAGCAGCCGCGGTAATACATAGGTGGCAAGCGTTATCCGGATTTATTGGGCGTATAGGGTGCGTAGGCGGTTTTGCAAGTTTGAGGTTAAAGTCCGGAGCTCAACTCCGGTTCGCCTTGAAAACTGTATTACTAGAATGCAAGAGAGGTAAGCGGAATTCCATGTGTAGCGGTGAAATGCGTAGATATATGGAAGAACACCTGTGGCGAAAGCGGCTTACTGGCTTGTTATTGACGCTGAGGCACGAAAGCGTGGGGAGCAAATAGGATTAGATACCCTAGTAGTCCACGCCGTAAACGATGAGTACTAAGTGTTGGGGAAACTCAGCGCTGCAGCTAACGCATTAAGTACTCCGCCTGAGTAGTATGCTCGCAAGAGTGAAACTCAAAGGAATTGACGGGGACCCGCACAAGTGGTGGAGCATGTGGTTTAATTCGAAGCAACACGAAGAACCTTACCAGGGCTTGACATCCAGTGCAAAGCTATAGAGATATAGTAGAGGTTAACATTGAGACAGGTGGTGCATGGTTGTCGTCAGTTCGTGCCGTAAGGTGTTGGGTTAAGTCCCGCAACGAACGCAACCCTTGTCGCTAGTTACTAACATTAAGTTGAGAACTCTAACGAGACTGCTAGTGTAAGCTAGAGGAAGGTGGGGATGACGTCAAATCATCATGCCCCTTATGTCCTGGGCTACACACGTGCTACAATGGCTGGTACAAAGAGTTGCAATCCTGTGAAGGGGAGCTAATCTCAAAAAAACCAGTCTCAGTTCGGATTGAAGTCTGCAACTCGACTTCATGAAGCCGGAATCACTAGTAATCGCGAATCAGCTATGTCGCGGTGAATACGTTCTCGGGTCTTGTACACACCGCCCGTCACACCATGAGAGTTGGTAATACCAGAAGTAGGTAGCTTAACCATTTGGAGAGCGCTTCCCAAGGTAGGACTAGCGATTGGGGTGAAGTCGTAACAAGGTATCCGTACGGGAACGTGCGGATGGATCACCTCCTTTCTA

>*M. mycoides* subsp. *capri* 95010 (MLC_0626)

TAAAATGAGAGTTTGATCCTGGCTCAGGATAAACGCTGGCGGCATGCCTAATACATGCAAGTCGAACGGAGGTGCTTGCACCTCAGTGGCGAACGGGTGAGTAACACGTATCTAACCTACCTCATAGCGGGGGATAACTTTTGGAAACGAAAGATAATACCGCATGTAGATCTTATTATCGCATGAGAAAAGATCAAAAGAACCGTTTGGTTCACTATGAGATGGGGATGCGGCGTATTAGCTAGTAGGTGAGATAATAGCCCACCTAGGCGATGATACGTAGCCGAACTGAGAGGTTGATCGGCCACATTGGGACTGAGATACGGCCCAGACTCCTACGGGAGGCAGCAGTAGGGAATTTTTCACAATGGACGAAAGTCTGATGAAGCAATGCCGCGTGAGTGATGACGGCCTTCGGGTTGTAAAGCTCTGTTGTAAGGGAAGAAAAAATAAAGTAGGAAATGACTTTATCTTGACAGTACCTTACCAGAAAGCCACGGCTAACTATGTGCCAGCAGCCGCGGTAATACATAGGTGGCAAGCGTTATCCGGATTTATTGGGCGTATAGGGTGCGTAGGCGGTTTTGCAAGTTTGAGGTTAAAGTCCGGAGCTCAACTCCGGTTCGCCTTGAAAACTGTATTACTAGAATGCAAGAGAGGTAAGCGGAATTCCATGTGTAGCGGTGAAATGCGTAGATATATGGAAGAACACCTGTGGCGAAAGCGGCTTACTGGCTTGTTATTGACGCTGAGGCACGAAAGCGTGGGGAGCAAATAGGATTAGATACCCTAGTAGTCCACGCCGTAAACGATGAGTACTAAGTGTTGGGGTAACTCAGCGCTGCAGCTAACGCATTAAGTACTCCGCCTGAGTAGTATGCTCGCAAGAGTGAAACTCAAAGGAATTGACGGGGACCCGCACAAGTGGTGGAGCATGTGGTTTAATTCGAAGCAACACGAAGAACCTTACCAGGGCTTGACATCCAGTGCAAAGCTATAGAGATATAGTAGAGGTTAACATTGAGACAGGTGGTGCATGGTTGTCGTCAGTTCGTGCCGTGAGGTGTTGGGTTAAGTCCCGCAACGAACGCAACCCTTGTCGTTAGTTACTAACATTAAGTTGAGAACTCTAACGAGACTGCTAGTGTAAGCTAGAGGAAGGTGGGGATGACGTCAAATCATCATGCCCCTTATGTCCTGGGCTACACACGTGCTACAATGGCTGGTACAAAGAGTTGCAATCCTGTGAAGGGGAGCTAATCTCAAAAAACCAGTCTCAGTTCGGATTGAAGTCTGCAACTCGACTTCATGAAGCCGGAATCACTAGTAATCGCGAATCAGCTATGTCGCGGTGAATACGTTCTCGGGTCTTGTACACACCGCCCGTCACACCATGAGAGTTGGTAATACCAGAAGTAGGTAGCTTAACCGTTTGGAGAGCGCTTCCCAAGGTAGGACTAGCGATTGGGGTGAAGTCGTAACAAGGTATCCGTACGGGAACGTGCGGATGGATCACCTCCTTTCTA

> *M. mycoides* subsp. *capri* GM12 (MMCAP2_0069)

AAAATGAGAGTTTGATCCTGGCTCAGGATAAACGCTGGCGGCATGCCTAATACATGCAAGTCGAACGGAGGTGCTTGCACCTCAGTGGCGAACGGGTGAGTAACACGTATCTAACCTACCTCATAGCGGGGGATAACTTTTGGAAACGAAAGATAATACCGCATGTAGATCTTATTATCGCATGAGAAAAGATCAAAAGAACCGTTTGGTTCACTATGAGATGGGGATGCGGCGTATTAGCTAGTAGGTGAGATAATAGCCCACCTAGGCGATGATACGTAGCCGAACTGAGAGGTTGATCGGCCACATTGGGACTGAGATACGGCCCAGACTCCTACGGGAGGCAGCAGTAGGGAATTTTTCACAATGGACGAAAGTCTGATGAAGCAATGCCGCGTGAGTGATGACGGCCTTCGGGTTGTAAAGCTCTGTTGTAAGGGAAGAAAAAATAAAGTAGGAAATGACTTTATCTTGACAGTACCTTACCAGAAAGCCACGGCTAACTATGTGCCAGCAGCCGCGGTAATACATAGGTGGCAAGCGTTATCCGGATTTATTGGGCGTATAGGGTGCGTAGGCGGTTTTGCAAGTTTGAGGTTAAAGTCCGGAGCTCAACTCCGGTTCGCCTTGAAAACTGTATTACTAGAATGCAAGAGAGGTAAGCGGAATTCCGTGTGTAGCGGTGAAATGCGTAGATATATGGAAGAACACCTGTGGCGAAAGCGGCTTACTGGCTTGTTATTGACGCTGAGGCACGAAAGCGTGGGGAGCAAATAGGATTAGATACCCTAGTAGTCCACGCCGTAAACGATGAGTACTAAGTGTTGGGGTAACTCAGCGCTGCAGCTAACGCATTAAGTACTCCGCCTGAGTAGTATGCTCGCAAGAGTGAAACTCAAAGGAATTGACGGGGACCCGCACAAGTGGTGGAGCATGTGGTTTAATTCGAAGCAACACGAAGAACCTTACCAGGGCTTGACATCCAGTGCAAAGCTATAGAGATATAGTAGAGGTTAACATTGAGACAGGTGGTGCATGGTTGTCGTCAGTTCGTGCCGTGAGGTGTTGGGTTAAGTCCCGCAACGAACGCAACCCTTGTCGTTAGTTACTAACATTAAGTTGAGAACTCTAACGAGACTGCTAGTGTAAGCTAGAGGAAGGTGGGGATGACGTCAAATCATCATGCCCCTTATGTCCTGGGCTACACACGTGCTACAATGGCTGGTACAAAGAGTTGCAATCCTGTGAAGGGGAGCTAATCTCAAAAAACCAGTCTCAGTTCGGATTGAAGTCTGCAACTCGACTTCATGAAGCCGGAATCACTAGTAATCGCGAATCAGCTATGTCGCGGTGAATACGTTCTCGGGTCTTGTACACACCGCCCGTCACACCATGAGAGTTGGTAATACCAGAAGTAGGTAGCTTAACCGTTTGGAGAGCGCTTCCTAAGGTAGGACTAGCGATTGGGGTGAAGTCGTAACAAGGTATCCGTACGGGAACGTGCGGATGGATCACCTCCTTTCT

>*M. capricolum* subsp. *capricolum* California Kid (NR_036952.1)

AAAATGAGAGTTTGATCCTGGCTCAGGATAAACGCTGGCGGCATGCCTAATACATGCAAGTCGAACGGGGGTGCTTGCACCTCAGTGGCGAACGGGTGAGTAACACGTATCTAACCTACCTTATAGCGGGGGATAACTTTTGGAAACGAAAGATAATACCGCATGTAGATCTTATTATCGCATGAGAAAAGATCAAAAGAACCGTTTGGTTCACTATGAGATGGGGATGCGGCGTATTAGCTAGTAGGTGAGATAATAGCCCACCTAGGCGATGATACGTAGCCGAACTGAGAGGTTGATCGGCCACATTGGGACTGAGATACGGCCCAGACTCCTACGGGAGGCAGCAGTAGGGAATTTTTCACAATGGACGAAAGTCTGATGAAGCAATGCCGCGTGAGTGATGACGGCCTTCGGGTTGTAAAGCTCTGTTGTAAGGGAAGAAAAAATAGAGTAGGAAATGACTTTATCTTGACAGTACCTTACCAGAAAGCCACGGCTAACTATGTGCCAGCAGCCGCGGTAATACATAGGTGGCAAGCGTTATCCGGATTTATTGGGCGTATAGGGTGCGTAGGCGGTTTTGCAAGTTTGAGGTTAAAGTCCGGAGCTCAACTCCGGTTCGCCTTGAAGACTGTTTTACTAGAATGCAAGAGAGGTAAGCGGAATTCCATGTGTAGCGGTGAAATGCGTAGATATATGGAAGAACACCTGTGGCGAAAGCGGCTTACTGGCTTGTTATTGACGCTGAGGCACGAAAGCGTGGGGAGCAAATAGGATTAGATACCCTAGTAGTCCACGCCGTAAACGATGAGTACTAAGTGTTGGGGTAACTCAGCGCTGCAGCTAACGCATTAAGTACTCCGCCTGAGTAGTATGCTCGCAAGAGTGAAACTCAAAGGAATTGACGGGGACCCGCACAAGTGGTGGAGCATGTGGTTTAATTCGAAGCAACACGAAGAACCTTACCAGGGCTTGACATCCAGTGCAAAGCTATAGAGATATAGTAGAGGTTAACATTGAGACAGGTGGTGCATGGTTGTCGTCAGTTCGTGCCGTGAGGTGTTGGGTTAAGTCCCGCAACGAACGCAACCCTTGTCGTTAGTTACTAACATTAAGTTGAGAACTCTAACGAGACTGCTAGTGTAAGCTAGAGGAAGGTGGGGATGACGTCAAATCATCATGCCCCTTATGTCCTGGGCTACACACGTGCTACAATGGCTGGTACAAAGAGTTGCAATCCTGTGAAGGGGAGCTAATCTCAAAAAACCAGTCTCAGTTCGGATTGAAGTCTGCAACTCGACTTCATGAAGCCGGAATCACTAGTAATCGCGAATCAGCTATGTCGCGGTGAATACGTTCTCGGGTCTTGTACACACCGCCCGTCACACCATGAGAGTTGGTAATACCAGAAGTAGGTAGCTTAACCATTTGGAGAGCGCTTCCCAAGGTAGGACTAGCGATTGGGGTGAAGTCGTAACAAGGTATCCGTACGGGAACGTGCGGATGGATCACCTCCTTTCT

>*M. feriruminatoris* G5847 (D500_0527)

GAGTTTGATCCTGGCTCAGGATAAACGCTGGCGGCATGCCTAATACATGCAAGTCGAACGGAAGTGCTTGCACTTCAGTGGCGAACGGGTGAGTAACACGTATCTAACCTACCTTATAGCGGGGGATAACTTTTGGAAACGAAAGATAATACCGCATGTAGATCTTATTATCGCATGAGAAAAGATCAAAAGAACCGTTTGGTTCACTATGAGATGGGGATGCGGCGTATTAGCTAGTAGGTGAGATAATAGCCCACCTAGGCGATGATACGTAGCCGAACTGAGAGGTTGATCGGCCACATTGGGACTGAGATACGGCCCAGACTCCTACGGGAGGCAGCAGTAGGGAATTTTTCACAATGGACGAAAGTCTGATGAAGCAATGCCGCGTGAGTGATGACGGCCTTCGGGTTGTAAAGCTCTGTTGTAAGGGAAGAAAAAATAAAGTAGGAAATGACTTTATCTTGACAGTACCTTACCAGAAAGCCACGGCTAACTATGTGCCAGCAGCCGCGGTAATACATAGGTGGCAAGCGTTATCCGGATTTATTGGGCGTATAGGGTGCGTAGGCGGTTTAGCAAGTTTGAGGTTAAAGTCCGGAGCTCAACTCCGGTTCGCCTTGAAAACTGTTTTACTAGAATGCAAGAGAGGTAAGCGGAATTCCATGTGTAGCGGTGAAATGCGTAGATATATGGAAGAACACCTGTGGCGAAAGCGGCTTACTGGCTTGTTATTGACGCTGAGGCACGAAAGCGTGGGGAGCAAATAGGATTAGATACCCTAGTAGTCCACGCCGTAAACGATGAGTACTAAGTGTTGGGGAAACTCAGCGCTGCAGCTAACGCATTAAGTACTCCGCCTGAGTAGTATGCTCGCAAGAGTGAAACTCAAAGGAATTGACGGGGACCCGCACAAGTGGTGGAGCATGTGGTTTAATTCGAAGCAACACGAAGAACCTTACCAGGGCTTGACATCCAGTGCAAAGCTATAGAAATATAGTAGAGGTTAACATTGAGACAGGTGGTGCATGGTTGTCGTCAGTTCGTGCCGTGAGGTGTTGGGTTAAGTCCCGCAACGAACGCAACCCTTGTCGTTAGTTACTAACATTAAGTTGAGAACTCTAACGAGACTGCTAGTGTAAGCTAGAGGAAGGTGGGGATGACGTCAAATCATCATGCCCCTTATGTCCTGGGCTACACACGTGCTACAATGGCTGGTACAAAGAGTTGCAATCCTGTGAAGGGGAGCTAATCTCAAAAAACCAGTCTCAGTTCGGATTGAAGTCTGCAACTCGACTTCATGAAGCCGGAATCACTAGTAATCGCGAATCAGCTATGTCGCGGTGAATACGTTCTCGGGTCTTGTACACACCGCCCGTCACACCATGAGAGTTGGTAATACCAGAAGTAGGTAGCTTAACCGCAAGGAGAGCGCTTCCTAAGGTAGGACTAGCGATTGGGGTGAAGTCGTAACAAGGTATCCGTACGGGAACGTGCGGATGGATCACC

>*M. putrefaciens* KS1 (NR_025971.1)

CTGGCGGCATGCCTAATACATGCAAGTCGAACGGGGGTGCTTGCACCCCAGTGGCGAACGGGTGAGTAACACGTATCTAACCTACCTTATAGCGGGGGATAACTTTTGGAAACGAAAGATAATACCGCATGTGAATCTTATTATCGCATGAGAAAAGATTGAAAGAACCGTTTGGTTCACTATGAGATGGGGATGCGGCGTATTAGCTAGTAGGTGAGGTAATGGCTCACCTAGGCGATGATACGTAGCCGAACTGAGAGGTTGATCGGCCACATTGGGACTGAGATACGGCCCAGACTCCTACGGGAGGCAGCAGTAGGGAATTTTTCACAATGGACGAAAGTCTGATGAAGCAATGCCGCGTGAGTGATGACGGCCTTCGGGTTGTAAAGCTCTGTTGTAAGGGAAGAAAAAATAAAGTAGGAAATGCCTTTATATTGACGGTACCTTACCAGAAAGCCACGGCTAACTATGTGCCAGCAGCCGCGGTAATACATAGGTGGCAAGCGTTATCCGGATTTATTGGGCGTATAGGGTGCGTAGGCGGTTGTGCAAGTTTGAGGTTAAAGTCCGGAGCTCAACTCCGGTTCGCCTTGAAAACTGCATTACTAGAATACAAGAGAGGTAAGCGGAATTCCATGTGTAGCGGTGAAATGCGTAGATATATGGAAGAACACCTGTGGCGAAAGCGGCTTACTGGTTTGTTATTGACGCTGAGGCACGAAAGCGTGGGGAGCAAATAGGATTAGATACCCTAGTAGTCCACGCCGTAAACGATGAGTACTAGGTGTTGGGTGAACTCAGCGCCGCAGCTAACGCATTAAGTACTCCGCCTGAGTAGTATGCTCGCAAGAGTGAAACTCAAAGGAATTGACGGGGACCCGCACAAGTGGTGGAGCATGTGGTTTAATTCGAAGCAACACGAAGAACCTTACCAGGGCTTGACATCCAGTGCAAAGCTATAGAGATATAGTAGAGGTTAACATTGAGACAGGTGGTGCATGGTTGTCGTCAGTTCGTGCCGTGAGGTGTTGGGTTAAGTCCCGCAACGAACGCAACCCTTGTCGTTAGTTACTAACATTAAGTTGAGGACTCTAACGAGACTGCTAGTGTAAGCTAGAGGAAGGTGGGGATGACGTCAAATCATCATGCCCCTTATGTCCTGGGCTACACACGTGCTACAATGGCTGGTACAAAGAGTCGCAATCTCGCGAGGGGGAGCTAATCTCAAAAAACCAGTCTCAGTTCGGATTGAAGTCTGCAACTCGACTTCATGAAGCCGGAATCACTAGTAATCGCGAATCAGCTATGTCGCGGTGAATACGTTCTCGGGTCTTGTACACACCGCCCGTCACACCACGAGAGTTGGTAATACCAGAAGTGGGTAGCTTAACCGCAAGGAGAGCGCCTCCCAAGGTAGGACTAGCGATTGGGGTGAAGTCGTAACAAGGTATCCGTACGGGAAC

>*M. florum* L1(NR_025128.1)

CTGGCTCAGGAYGAACGCTGGCGGCATGCCTAATACATGCAAGTCGAACGGAGGTGCTTGCACCTCAGTGGCGAACGGGTGAGTAACACGTATCTAATCTACCTTCTAGCGGGGGATAACTTTTGGAAACGAAAGGTAATACCGCATGTGGATGTTATTATCGCATGAGAAAACATTCAAAGATCCGTTTGGATCACTAGAAGATGAGGATGCGGCGTATTAGCTAGTAGGCGGGGTAAAGGCCCACCTAGGCGATGATACGTAGCCGAACTGAGAGGTTGATCGGCCACATTGGGACTGAGATACGGCCCAGACTCCTACGGGAGGCAGCAGTAGGGAATTTTTCACAATGGACGAAAGTCTGATGAAGCAATGCCGCGTGAGTGATGACGGCCTTCGGGTTGTAAAGCTCTGTTGTAAGGGAAGAAAACATAGGAGAGGAAATGCTCTTATCTTGACGGTACCTTACCAGAAAGCCACGGCTAACTATGTGCCAGCAGCCGCGGTAATACATAGGTGGCAAGCGTTATCCGGATTTATTGGGCGTATAGGGTGCGTAGGCGGTTTCGCAAGTTTGAGGTTAAAGCCCGGAGCTCAACTCCGGTTCGCCTTGAAAACTGCGGGACTAGAATATCAGAGAGGTAAGCGGAATTCCATGTGTAGCGGTAAAATGCGTAGATATATGGAAGAACACCAGTGGCGAAAGCGGCTTACTGGCTGATTATTGACGCTGAGGCACGAAAGCGTGGGGAGCAAATAGGATTAGATACCCTAGTAGTCCACGCCGTAAACGTTGAGTACTAAGTATTGGGGATTACCTCAGTGCTGCAGCTAACGCATTAAGTACTCCGCCTGAGTAGTATGCTCGCAAGAGTGAAACTCAAAGGAATTGACGGGGACCCGCACAAGTGGTGGAGCATGTGGTTTAATTCGAAGCAACACGAAGAACCTTACCAGGGCTTGACATACAGTGCAAAGCTACAGAGATGTAGTGGAGGTTAACATTGATACAGGTGGTGCATGGTTGTCGTCAGTTCGTGCCGTGAGGTGTTGGGTTAAGTCCCGCAACGAACGCAACCCTTGTCGTTAGTTACTAACATTAAGTTGAGGACTCTAACGAGACTGCTAGTGTAAGCTAGAGGAAGGTGGGGATGACGTCAAATCATCATGCCCCTTATGTCCTGGGCTACACACGTGCTACAATGGCCGATACAAAGAGTCGCAATCTCGCGAGGGGGAGCTAATCTCAAAAAGTCGGTCTCAGTTCGGATTGAAGTCTGCAACTCGACTTCATGAAGCCGGAATCACTAGTAATCGCGAATCAGCTATGTCGCGGTGAATACGTTCTCGGGTCTTGTACACACCGCCCGTCAAACCACGAGAGTTGGTAATACCAGAAGTACGTTTCCTAACCGTAAGGAAGGCGCGTCCCAAGGTAGGACTAGCGATTGGGGTTAAGTCGTAACAAGGTATCCGTACGGGAACGTGCGGATGGATCAC

>*Spiroplasma citri* GII3

TTTTAATGAGAGTTTGATCCTGGCTCAGGATGAACGCTGGCGGCATGCCTAATACATGCAAGTCGAACGGGGTGCTTGCACCCAGTGGCGAACGGGTGAGTAACACGTATCTAATCTACCCATTAGCGGGGGATAACAGTTGGAAACGACTGATAATACCGCATACGACATTTTCTGGCATCAGAGAATGTTAAAAGGTCCGTTTGGATCACTAATGGATGAGGATGCGCCGTATTAGTTAGTTGGTGGGGTAATGGCCTACCAAGACAATGATACGTAGCCGAACTGAGAGGTTGATCGGCCACATCGGGACTGAGACACGGCCCGAACTCCTACGGGAGGCAGCAGTAGGGAATTTTTCACAATGGGCGAAAGCCTGATGGAGCAATGCCGCGTGACTGAAGACGGTCTTCGGATTGTAAAAGTCTGTTGTAAGGGAAGAACAGTAAGTATAGGAAATGATACTTATTTGACGGTACCTTACCAGAAAGCCACGGCTAACTATGTGCCAGCAGCCGCGGTAATACATAGGTGGCAAGCGTTATCCGGATTTATTGGGCGTAAAGCGTGCGCAGACGGTTTAACAAGTTTGGGGTCAAATCCTGGAGCTCAACTCCAGTTCGCCTTGAAAACTGTTAAGCTAGAGTGTAGGAAAGGTCGATGGAATTCCATGTGTAGCGGTGAAATGCGTAGATATATGGAGGAACACCAGTGGCGAAGGCGGTCGACTGGCCTATCACTGACGTTTAGGCACGAAAGCGTAGGGAGCAAATAGGATTAGATACCCTAGTAGTCTACGCCGTAAACGATGAGTACTAAGTGTCGGACTAAGTTCGGTGCTGCAGCTAACGCATTAAGTACTCCGCCTGAGTAGTATGCTCGCAAGAGTGAAACTCAAAGGAATTGACGGGGACCCGCACAAGCGGTGGAGCATGTGGTTTAATTCGAAGCAACGCGAAGAACCTTACCAAGGCTTGACATCCAGTGCAAAGCTGTAGAAATACAGTGGAGGTTAACATTGAGACAGGTGGTGCATGGTTGTCGTCAGCTCGTGCCGTGAGGTGTTTGGTTAAGTCCAGTAACGAGCGCAACCCTTGCCGTTAGTTACTCCATTAAGTTGAGATACTCTAACAGGACTGCTAGTGTAAGCTAGAGGAAGGTGGGGATGACGTCAAATCAGCATGCCCCTTATATCTTGGGCTACACACGTGCTACAATGGTCGGTACAAACAGTTGCGATCTCGTAAGAGGGAGCTAATCTGAAAAAGCCGATCTCAGTTCGGATTGAGGGCTGCAACTCGCCCTCATGAAGCCGGAATCGCTAGTAATCGCGAATCAGCAATGTCGCGGTGAATACGTTCTCGGGTCTTGTACACACCGCCCGTCACACCATGAGAGTTGATAATACCAGAAGTCGGTATTCTAACCGCAAGGAGGAAGCCGCCCAAGGTAGGATTGATGATTAGGGTGAAGTCGTAACAAGGTATCCGTACGAGAACGTGCGGATGGATCACCTCCTTTCT

>*M. hominis* PG21 (NR_041881.1)

TTTTATAAGAGTTTGATCCTGGCTCAGGATGAACGCTGGCTGTGTGCCTAATACATGCATGTCGAGCGAGGTTAGCAATAACCTAGCGGCGAATGGGTGAGTAACACGTGCTTAATCTACCTTTTAGATTGGAATACCCATTGGAAACAATGGCTAATGCCGGATACGCATGGAACCGCATGGTTCCGTTGTGAAAGGCGCTGTAAGGCGCCACTAAAAGATGAGGGTGCGGAACATTAGTTAGTTGGTGAGGTAATGGCCCACCAAGACTATGATGTTTAGCCGGGTCGAGAGACTGAACGGCCACATTGGGACTGAGATACGGCCCAAACTCCTACGGGAGGCAGCAGTAGGGAATATTCCACAATGAGCGAAAGCTTGATGGAGCGACACAGCGTGCACGATGAAGGTCTTCGGATTGTAAAGTGCTGTTATAAGGGAAGAACATTTGCAATAGGAAATGATTGCAGACTGACGGTACCTTGTCAGAAAGCGATGGCTAACTATGTGCCAGCAGCCGCGGTAATACATAGGTCGCAAGCGTTATCCGGAATTATTGGGCGTAAAGCGTTCGTAGGCTGTTTGTTAAGTCTGGAGTTAAATCCCGGGGCTCAACCCCGGCTCGCTTTGGATACTAGCAAACTAGAGTTAGATAGAGGTAAGCGGAATTCCATGTGAAGCGGTGAAATGCGTAGATATATGGAAGAACACCAAAGGCGAAGGCAGCTTACTGGGTCTATACTGACGCTGAGGGACGAAAGCGTGGGGAGCAAACAGGATTAGATACCCTGGTAGTCCACGCCGTAAACGATGATCATTAGTCGGTGGAGAATCACTGACGCAGCTAACGCATTAAATGATCCGCCTGAGTAGTATGCTCGCAAGAGTGAAACTTAAAGGAATTGACGGGGACCCGCACAAGCGGTGGAGCATGTGGTTTAATTTGAAGATACACGGAAAACCTTACCCACTCTTGACATCCTTCGCAAAGCTATAGAGATATAGTGGAGGTTATCGGAGTGACAGATGGTGCATGGTTGTCGTCAGCTCGTGTCGTGAGATGTTTGGTCAAGTCCTGCAACGAGCGCAACCCCTATCTTTAGTTACTAACATTAAGTTGAGGACTCTAGAGATACTGCCTGGGTAACTGGGAGGAAGGTGGGGATGACGTCAAATCATCATGCCTCTTACGAGTGGGGCCACACACGTGCTACAATGGTCGGTACAAAGAGAAGCAATATGGCGACATGGAGCAAATCTCAAAAAGCCGATCTCAGTTCGGATTGGAGTCTGCAATTCGACTCCATGAAGTCGGAATCGCTAGTAATCGCAGATCAGCTATGCTGCGGTGAATACGTTCTCGGGTCTTGTACACACCGCCCGTCACACCATGGGAGCTGGTAATACCCAAAGTCGGTTTGCTAACCTCGGAGGCGACCGCCTAAGGTAGGACTGGTGACTGGGGTGAAGTCGTAACAAGGTATCCCTACGAGAACGTGGGGATGGATCACCTCCTTT

>*M. hyorhinis* GDL (MYM_0017)

AGAGTTTGATCCTAGCTCAGGATGAACGCTCGCTGTGTGCCTAATACATGCATGTTGAACGGGATGTAGCAATACATTCAGTAGCGAATGGGTGAGTAACACGTACCTAACCTACCTTTAAGACTGGGATAACTATTGGAAACAATAGCTAATACCGGATATAGTTATTTATCGCATGATGAGTAATAGAAAGGAGCTTCACAGCTTCACTTAAAAATGGGGGTGCGGAACATTAGTTAGTTGGTAGGGTAATGGCCTACCAAGACGATGATGTTTAGCTGGGCCGAGAGGCTGTACGGCCACACTGGGACTGAGATACGGCCCAGACTCCTACGGGAGGCAGCAGTAAGGAATTTTCCACAATGAGCGAAAGCTTGATGGAGCGACACAGCGTGCAGGATGAAGTTCTTCGGAATGTAAACTGCTGTTATAAGGGAAGAAAAAATAGAATAGGAAATGATTTTATCTTGACGGTACCTTATTAGAAAGCGACGGCAAACTATGTGCCAGCAGCCGCGGTAATACATAGGTCGCAAGCGTTATCCGGAATTATTGGGCGTAAAGCGTCCGTAGGTTTTTTGCTAAGTCTGGAGTTAAATGCTGAAGCTCAACTTCAGTCCGCTTTGGATACTGGCAAAATAGAATTATAAAGAGGTTAGCGGAATTCCTAGTGAAGCGGTGGAATGCGTAGATATTAGGAAGAACACCAATAGGCGAAGGCAGCTAACTGGTTATATATTGACACTAAGGGACGAAAGCGTGGGGAGCAAACAGGATTAGATACCCTGGTAGTCCACGCCGTAAACGATGATCATTAGTTGGTGGAATAATTTCACTAACGCAGCTAACGCGTTAAATGATCCGCCTGAGTAGTATGCTCGCAAGAGTGAAACTTAAAGGAATTGACGGGAACCCGCACAAGCGGTGGAGCATGTGGTTTAATTTGAAGATACGCGTAGAACCTTACCCACTCTTGACATCTTCTGCAAAGCTATAGAGATATAGTGGAGGTTAACAGAATGACAGATGGTGCATGGTTGTCGTCAGCTCGTGTCGTGAGATGTTAGGTTAAGTCCTGCAACGAGCGCAACCCTTTTCTTTAGTTACTAATATTAAGTTAAGGACTCTAGAGATACTGCCTGGGTAACCAGGAGGAAGGTGGGGACGACGTCAAATCATCATGCCTCTTACGAGTGGGGCAACACACGTGCTACAATGGTCGGTACAAAGAGAAGCAATATGGTGACATGGAGCAAATCTCAAAAAACCGATCTCAGTTCGGATTGAAGTCTGCAACTCGACTTCATGAAGTCGGAATCGCTAGTAATCGTAGATCAGCTACGCTACGGTGAATACGTTCTCGGGTTTTGTACACACCGCCCGTCACACCATGGGAGTTGGTAATGCCCAAAGTCGGTGAGTTAACTTCGGAGACCATTGCCTAAGGCAGGACTGATGACTGGGGTGAAGTCGTAACAAGGTATCCCTACGAGAACGTGGGGATGGAACACCT

>*M. conjunctivae* HRC/581T (NR_044781.1)

CTTGCTGTGTGCCTAATACATGCATGTTGAACGGGATGTTTTAGTTTACTAAAACATTTAGTAGCGAATGGGTGAGTAACACGTACCTAACCTACCTTTAGGATTGGGATAACTACTGGAAACAGTAGCTAATACCGAATATAATATTTTTTCGCATGAAGAAATATAGAAAGGAGCCTTTAAAGCTTCACCTAGAAATGGGGGTGCGGAACATTAGTTAGTTGGTGAGGTAATGGCTCACCAAGACAATGATGTTTAGCGGGGCCAAGAGGTTGTACCGCCACACTGGGACTGAGATACGGCCCAGACTCCTACGGGAGGCAGCAGTAAGGAATTTTCCACAATGAGCGAAAGCTTGATGGAGCGACACAGCGTGCAGGATGAAATCCCTCGGGATGTAAACTGCTGTTATAAGGAAAGAAAAAATCAAATAGGAAATGATTTGATCTTGACGGTACCTTATTAGAAAGCGACGGCAAACTATGTGCCAGCAGCCGCGGTAATACATAGGTCGCAAGCGTTATCCGGAATTATTGGGCGTAAAGCGTCCGTAGGTTTTTTGTTAAGTTTAAGGTTAAATACTGAAGCTCAACTTCAGCCCGCTTTAGATACTGGCAAAATAGAATTATGAAGAGGTTAGCGGAATTCCTAGTGTAGCGGTGGAATGCGTAGATATTAGGAAGAACACCAATAGGCGAAGGCAGCTAACTGGTCATACATTGACACTAAGGGACGAAAGCGTGGGGAGCAAACAGGATTAGATACCCTGGTAGTCCACGCTGTAAACGATGATCATTAGTTGGTGGTATTAAATCACTAACACAGCTAACGCGTTAAATGATCCGCCTGAGTAGTATGCTCGCAAGAGTGAAACTTAAAGGAATTGACGGGAACCCGCACAAGCGGTGGAGCATGTGGTTTAATTTGAAGATACGCGTAGAACCTTACCCACTCTTGACATCCTTGCAAAGCTATAGAGATATAGTGGAGGCTAACAAGATGACAGATGGTGCATGGTTGTCGTCAGCTCGTGTCGTGAGATGTTAGGTTAAGTCCTGCAACGAGCGCAACCCCTTTCTTTAGTTACTAACGTGAAAGACGAGGACTCTAAAGATACTGCCTGTGTAAGCCGGAGGAAGGTGGGGACGACGTCAAATCATCATGCCTCTTACGAGTGGGGCAACACACGTGCTACAATGGCTACTACAAAGAGACGCAAGACGGTGACGTTAAGCAAATCTCAAAAAAGTAGTCCCAGTTCGGATTGAAGTCTGCAACTCGACTTCATGAAGTCGGAATCGCTAGTAATCGCAGATCAGCTACGCTGCGGTGAATACGTTCTCGGGTTTTGTACACACCGCCCGTCACACCATGGGAGTTGGTAATGCCCAAAGTCGGTGAGTTAACTTCGGAGACCATTGCCTAAGGCAGGACTGATGACTGGGGTGAAGTCGTAACAAGGTATCCCTACGAGAAC

>*M. hyopneumoniae* J (NR_117470.1)

ACGCTAGCTGTGTGCTTATACATGCATGTTGAACGGAATATTTTAGTTCGCTAAAATATTTAGTAGCAAATGGGTGAGTAACACGTACCTAACCTACCTTTTGGACTGGGATAACCATTGGAAACAGTGGCTAATACCGGATATGATAAAAATTTGCATGAATTTTTATTCAAAGGAGCTTTCAAGCTTCACCAAGAAATGGGGGTGCGCAACATTAGTTAGTTGGTAGGGTAAAAGCCTACCAAGACGATGATGTTTAGCGGGGCCAAGAGGTTGTACCGCCACACTGGGATTGAGATACGGCCCAGACTCCTACGGGAGGCAGCAGTAAGGAATATTCCACAATAAGCGAAAGCTTGATGGAGCGACACAGCGTGCAGGATGAAGTCTTTCGGGATGTAAACTGCTGTTGTAAGGGAAGAAAAAACTAGATAGGAAATGCTCTAGTCTTGACGGTACCTTATTAGAAAGCGACGGCAAACTATGTGCCAGCAGCCGCGGTAATACATAGGTCGCAAGCGTTATCCGGAATTATTGGGCGTAAAGCGTCCGTAGGTTTTTTGTTAAGTTTAAAGTTAAATGCTAAAGCTCAACTTTAGTCCGCTTTAGATACTGGCAAAATAGAATTATGAAGAGGTTAGCGGAATTCCTAGTGGAGTGGTGGAATACGTAGATATTAGGAAGAACACCAATAGGCGAAGGCAGCTAACTGGTCATATATTGACACTAAGGGACGAAAGCGTGGGGAGCAAACAGGATTAGATACCCTGGTAGTCCACGCCGTAAACGATGATCATTAGTTGGTGGCAAAAGTCACTAACACAGCTAACGCGTTAAATGATCCGCCTGAGTAGTATGCTCGCAAGAGTGAAACTTAAAGGAATTGACGGGAACCCGCACAAGCGGTGGAGCATGTGGTTTAATTTGATGATACGCGTAGAACCTTACCCACTCTTGACATTCTCGCAAAACTATAGAGATATAGCCGAGGCTAACGAGATCACAGATGGTGCATGGTTGTCGTCAGCTCGTGTCGTGAGATGTTAGGTTAAGTCCTGCAACGAGCGCAACCCTTTTCTTTAGTTGCTAACATTTAGTTGAGAACCCTAAAGATACTGCCGGCGCAAGCCGGAGGAAGGTGGGGACGACGTCAAATCATCATGCCTCTTACGAGTGGGGCAACACACGTGCTACAATGGCTACTACAAAGAGCAGCAAAACAGTGATGTCAAGCTAATCTCAAAAAAGTAGTCTCAGTTCGGATTGAAGTCTGCAACTCGACTTCATGAAGTCGGAATCGCTAGTAATCGCAGGTCAGCTATACTGCGGTGAATACGTTCTCGGGTTTTGTACACACCGCCCGTCACACCATGGGAGTTGGTAATGCCCAAAGTCGGTGAGTAACTTCGGAGACCATGCCTAAAGGCAGACCGAGACT

>*M. synoviae* 53

AGATGAACGCTGGCTGTGTGCCTAATACATGCATGTCGAGCGGAATTTAGCAATAAATTTAGCGGCGAATGGGTGAGTAACACGTACTTAACGTACCTTTTAGACTGGAATAACGGTGAGAAATTATCGCTAATGCCGGATATATAAAAAAATCGCATGATTTTTTTAAGAAAGAAGCGTTTGCTTCACTAAGAGATCGGGGTGCGGAACATTAGCTAGTTGGTAGGGTAATGGCCTACCAAGGCAATTATGTTTAGCGGGGTTGAGAGACTGAACCGCCACACTGGGACTGAGATACGGCCCAGACTCCTACGGGAGGCAGCAGTAGGGAATTTTCCACAATGGGCGAAAGCCTGATGGAGCAACACAGCGTGTAGGATGAAGGCCTTCGGGTTGTAAACTACTGTTATATGGGAAGAAAAACTAGTATAGGAAATGATATTAGCTTGACGGTACCATGTCAGAAAGCAACGGCTAACTATGTGCCAGCAGCCGCGGTAATACATAGGTTGCAAGCGTTATCCGGAATTATTGGGCGTAAAGCGTCTGTAGGTTGTTTGTTAAGTCTGGTGTTAAAACTTGGAGCTCAACTCCAAATTGCATTGGATACTGGCAGACTAGAATTGTTTAGAGGTTAGCGGAATTCCTTGTGAAGCGGTGGAATGCGTAGATATAAGGAAGAACACCAACATGGCGAAGGCAGCTAACTGGGAACATATTGACACTGAGAGACGAAAGCGTGGGGAGCAAACAGGATTAGATACCCTGGTAGTCCACGCTGTAAACGATGATGACTAGTTGATGGAAACCATCGACGCAGCTAACGCATTAAGTCATCCGCCTGAGTAGTATGCTCGCAAGAGTGAAACTTAAAGGAATTGACGGGGATCCGCACAAGCGGTGGAGCATGTGGTTTAATTTGAAGATACGCGTAGAACCTTACCCACTCTTGACATCTTCTGCAAAGCTATAGAGATATAGTAGAGGTTAACAGAATGACAGATGGTGCATGGTTGTCGTCAGCTCGTGTCGTGAGATGTTCGGTTAAGTCCTGCAACGAGCGCAACCCTTGTCCTTAGTTACTTTATCTAAGGAGACTGCCCGAGTAATTGGGAGGAAGGTGGGGACGACGTCAAATCATCATGCCTCTTACGAGTGGGGCAACACACGTGCTACAATGGACGATACAAAGAGAAGCAAAATAGTGATATCAAGCAAATCTCAAAAAATCGTTCTCAGTTCGGATTGTAGTCTGCAACTCGACTACATGAAGTCGGAATCGCTAGTAATCGTAGATCAGCTACGCTACGGTGAATACGTTCTCGGGTCTTGTACACACCGCCCGTCACACCATGGGAGCTGGTAATGCCCGAAGTCGGTTTGTTAACTTCGGAGACGACTGCCTAAGGCAGGACCGGTGACTGGGGTGAAGTCGTAACAAGGTATCCCTACGAGAACGTGGGGATGGATTACCTCCTTTCTTACGGAGTACATTAATTTTACAAAAGGCATTTTTTATTAACTGAAAGCT

>*M. crocodylii* MP145 (NR_074301.1)

TTGATCCTGGCTCAGGATGAACGCTGGCTGTGTGCCTAATACATGCATGTCGAGCGGAGCCTTTCGGGGCTTAGCGGCGAATGGGTGAGTAACACGTGCCTAACGTGCCTCTTAGATTGGAATAACGATTAGAAATGATCGCTAATGCCGGATACTTATTAGTTTCACATGAAACTAATATAAAAGGAGCGTTTGCTCCACTAAGAGATCGGGGTGCGTAACATTAGTTTGTTGGTGGGGTAATGGCTCACCAAGACGATGATGTTTAGCGGGGTTGAGAGACTGATCCGCCACACTGGGACTGAGATACGGCCCAGACTCCTACGGGAGGCAGCAGTAGGGAATTTTCCACAATGGACGAAAGTCTGATGGAGCGACACAGCGTGCAGGAAGACGGCCTTCGGGTTGTAAACTGCTGTTATAAGGGAAGAAAAAGTAGTGCAGGAAATGGCATTACCTTGACGGTACCTTATCAGAAAGCAACGGCTAACTATGTGCCAGCAGCCGCGGTAATACATAGGTTGCAAGCGTTATCCGGAATTATTGGGCGTAAAGCGTCTGTAGGTTGTTTGTTAAGTCTGACGTTAAAACTTGGAGCTCAACTCCAAATTGCGTTGGATACTGGCAAGCTAGAGTTATGTAGAGGTAAGCGGAATTCCTTGTGAAGCGGTGAAATGCGTAGATATAAGGAAGAACATCAATTGGCGAAGGCAGCTTACTGGGCATATACTGACACTGAGAGACGAAAGCGTGGGGAGCAAACAGGATTAGATACCCTGGTAGTCCACGCCGTAAACGATGATGATTAGCTGATGGGAACCATCGGCGCAGCTAACGCATTAAATCATCCGCCTGAGTAGTATGCTCGCAAGAGTGAAACTTAAAGGAATTGACGGGGACCCGCACAAGCGGTGGAGCATGTGGTTTAATTTGAAGATACGCGAAGAACCTTACCCTCTCTTGACATCTTCCGCAAAGCTATAGAGATATAGTGGAGGTTAACGGAAAGACAGATGGTGCATGGTTGTCGTCAGCTCGTGTCGTGAGATGTTTGGTTAAGTCCTGCAACGAGCGCAACCCTTATCCTTAGTTAAATATTCTAGGGAGACTGCCCGAGTAATCGGGAGGAAGGTGGGGATGACGTCAAATCATCATGCCTCTTACGAGAGGGGCAACACACGTGCTACAATGGGAAGTACAAAGAGACGCAATACGGCGACGTTAAGCAAATCTCATAAAACTTCTCTCAGTTCGGATTGGAGTCTGCAACTCGACTCCATGAAGTCGGAATCGCTAGTAATCGTAGATCAGCTACGCTACGGTGAATACGTTCTCGGGTCTTGTACACACCGCCCGTCACACCATGGGAGCTGGTAATGCCCGAAGTCGGTTTTGTCAACTACGGAGACAACTGCCTAAGGCAGGACTGGTGACTGGGGTGAAGTCGTAACAAGGTATCCCTACGAGAACGTGGGGATGGATTACCTC

>*M. anatis* 1340 (NR_025176.1)

CTGGCTGTGTGCCTAATACATGCATGTCGAGCGGAGTTCTTCGGAACTTAGCGGCGAATGGGTGAGTAACACGTACTTAACATACCCTTTAGATTGGAATAACGATTAGAAATGATCGCTAATGCCGGATACTTATATTTATCGCATGAAAAATATATAAAAGGAGCGTTTGCTTCGCTAGAGGATTGGGGTGCGTAACATTAGCTAGTTGGTAGGGTAATGGCCTACCAAGGCTATGATGTTTAGCGGGGTTGAGAGACTGATCCGCCACACTGGGACTGAGATACGGCCCAGACTCCTACGGGAGGCAGCAGTAGGGAATTTTCCACAATGGACGAAAGTCTGATGGAGCGACACAGCGTGCAGGATGACGGCCTTCGGGTTGTAAACTGCTGTTATAAGGGAAGAAAAAATAAAGTAGGAAATGACTTTATCTTGACGGTACCTTRTCAGAAAGCAACGGCTAACTATGTGCCAGCAGCCGCGGTAATACATAGGTTGCAAGCGTTATCCGGAATTATTGGGCGTAAAGCGTCTGTAGGTTGTTTGTTAAGTCTGACGTGAAAACTTGGGGCTCAACCCCAAATTGCGTTGGATACTGGCGAACTAGAGTTATGAAGAGGTTAGCGGAATTCCTTGTGAAGCGGTGAAATGCGTAGATATAAGGAAGAACACCAATTTGGCGAAGGCAGCTAACTGGGCATACACTGACACTGAGAGACGAAAGCGTGGGGAGCAAACAGGATTAGATACCCTGGTAGTCCACGCTGTAAACGATGATGATTAGCTGATGGGGAACTCATCGGCACAGCTAACGCATTAAATCATCCGCCTGAGTAGTATGCTCGCAAGAGTGAAACTTAAAGGAATTGACGGGGATCCGCACAAGCGGTGGAGCATGTGGTTTAATTTGATTCTACGCGTAGAACCTTACCCACTCTTGACATCTTCCGCAAAGCTATAGAGATATAGTGGAGGTCAACGGAATGACAGATGGTGCATGGTTGTCGTCAGCTCGTGTCGTGAGATGTTCGGTTAAGTCCTGCAACGAGCGCAACCCTTGTCCTTAGTTAAATGTTCTAAGGAGACTGCCCGAGTAATTGGGAGGAAGGTGGGGACGACGTCAAATCATCATGCCTCTTACGAGTGGGGCAACACACGTGCTACAATGGACGGTACAAAGAGACGCAATACGGCGACGTGGAGCAAATCTCAAAAAACCGTTCTCAGTTCGGATTGTAGTCTGCAACTCGACTACATGAAGTCGGAATCGCTAGTAATCGTAGATCAGCTACGCTACGGTGAATACGTTCTCGGGTCTTGTACACACCGCCCGTCACACCATGGGAGCTGGTAATGCCCGAAGTCGGTTTTGTTAACTACGGAGACAACTGCCTAAGGCAGGACTGGTGACTGGGGTGAAGTCGTAACAAGGTATCCCTACGAGAAC

>*M. fermentens* PG18 (NR_044666.2)

TTTTTCGAGAGTTTGATCCTGGCTCAGGATGAACGCTGGCTGTGTGCCTAATACATGCATGTCGAGCGAAGGTAGCAATACCTTAGCGGCGAATGGGTGAGTAACACGTGCTCAACGTACCCTTCAGTTTGGCATAGCGACTGGAAACAGTCGATAATTTCAAATACTCGTAGTTTTCGCATGAAGATTACGGAAAAGAAGCNTTTCTTCGCTGGAGGAGCGGGGTGCGTAACATTAGCTAGTTGGTGAGGTAACGGCCCACNAAGGCGATAATGTTTNGCGGGGTTGAGAGACTGAACCGCCACACTGGGACTGAGATACGGCCNNGACTCCTACGGGAGGCNGCNGTAGGGAATNTTCCACNATGGGCGAAAGCCTGATGGAGCGACACAGCGTGAAGGATGAAGGTCCTATGGATTGTAAACTTCTGTGGTAAGGGAAGAAAAGACAGAATAGGAAATGATTTTGTTTTGACGGTACCTNATTNGAAAGCAACGGCTAACTATGTGCCAGCAGCCGCGGTAATACATAGGTTGCNAGCGTTATCCGGAATTNTTGGGCGTAAAGCGTCTGTAGGTTGTTTGTTAAGTCTGGCGTTAAATTTTGGGGCTCAACCCCAAAACGCGTTGGATACTGGCAGGCTAGAGTTGTGTNGAGGTTAGCGGAATTCCTTGTGAAGCGGTGAAATGCGTAGATATAAGGAAGAACACCAAGATGGCGAAGGCAGCTAACTGGACATATACTGACACTGAGAGACGAAAGCGTGGGGAGCAAACAGGATTAGATACCCTGGTAGTCCACGCCCTAAACGATGATCATTAGCTGATGGGGAACTCATCGGCGCAGCTAACGCATTAAATGATCCGCCTGAGTAGTACGTTCGCAAGAATAAAACTTAAAGGAATTGACGGGGATCCGCACAAGCGGTGGAGCATGTGGTTTAATTTGAAGATACGCGTAGAACCTTACCCACTCTTGACATCTTCTGCAAAGCTATGGAGACATAGTGGAGGTTAACAGAATGACAGATGGTGCATGGTTGTCGTCAGCTCGTGTCGTGAGATGTTTGGTTAAGTCCTGCAACGAGCGCAACCCTTATCCTTAGTTACTACCATTTAGTTGAGGACTCTAAGGAGACTGCCCGAGTAATCGGGAGGAAGGTGGGGATGACGTCAAATCATCATGCCTCTTACGAGTGGGGCAACACACGTGCTACAATGGCCGGTACAAAGAGAAGCGAAGTGGTGACATGGAGCAAACCTCAAAAAACCGGTCTCAGTTCGGATTGTAGTCTGCAACTCGACTACATGAAGTCGGAATCGCTAGTAATCGTAGATCNCGTACGCTACGGTGAATACGTTCTCGGGTCTTGTACACACCGCCCGTCAAACCATGGGAGCTGGTAATGCCCGAAGTCGGTTTATNAACAAATCGCCTAAGGCAGGACTGGTGACTGGGG

>*M. fermentens* M64 (Molligen)

ATTTTTTCGAGAGTTTGATCCTGGCTCAGGATGAACGCTGGCTGTGTGCCTAATACATGCATGTCGAGCGAAGGTAGCAATACCTTAGCGGCGAATGGGTGAGTAACACGTGCTCAACGTACCCTTCAGTTTGGCATAGCGACTGGAAACAGTCGATAATTTCAAATACTCGTAGTTTTCGCATGAAGATTACGGAAAAGAAGCGTTTGCTTCGCTGGAGGAGCGGGGTGCGTAACATTAGCTAGTTGGTGAGGTAACGGCCCACCAAGGCGATGATGTTTAGCGGGGTTGAGAGACTGAACCGCCACACTGGGACTGAGATACGGCCCAGACTCCTACGGGAGGCAGCAGTAGGGAATATTCCACAATGGGCGAAAGCCTGATGGAGCGACACAGCGTGAAGGATGAAGGTCCTATGGATTGTAAACTTCTGTGGTAAGGGAAGAAAAGACAGAATAGGAAATGATTTTGTTTTGACGGTACCTTATTAGAAAGCAACGGCTAACTATGTGCCAGCAGCCGCGGTAATACATAGGTTGCAAGCGTTATCCGGAATTATTGGGCGTAAAGCGTCTGTAGGTTGTTTGTTAAGTCTGGCGTTAAATTTTGGGGCTCAACCCCAAAACGCGTTGGATACTGGCAGGCTAGAGTTGTGTAGAGGTTAGCGGAATTCCTTGTGAAGCGGTGAAATGCGTAGATATAAGGAAGAACACCAAGATGGCGAAGGCAGCTAACTGGACATATACTGACACTGAGAGACGAAAGCGTGGGGAGCAAACAGGATTAGATACCCTGGTAGTCCACGCCCTAAACGATGATCATTAGCTGATGGGGAACTCATCGGCGCAGCTAACGCATTAAATGATCCGCCTGAGTAGTACGTTCGCAAGAATAAAACTTAAAGGAATTGACGGGGATCCGCACAAGCGGTGGAGCATGTGGTTTAATTTGAAGATACGCGTAGAACCTTACCCACTCTTGACATCTTCTGCAAAGCTATGGAGACATAGTGGAGGTTAACAGAATGACAGATGGTGCATGGTTGTCGTCAGCTCGTGTCGTGAGATGTTTGGTTAAGTCCTGCAACGAGCGCAACCCTTATCCTTAGTTACTACCATTTAGTTGAGGACTCTAAGGAGACTGCCCGAGTAATCGGGAGGAAGGTGGGGATGACGTCAAATCATCATGCCTCTTACGAGTGGGGCAACACACGTGCTACAATGGCCGGTACAAAGAGAAGCGAAGTGGTGACATGGAGCAAACCTCAAAAAACCGGTCTCAGTTCGGATTGTAGTCTGCAACTCGACTACATGAAGTCGGAATCGCTAGTAATCGTAGATCAGCTACGCTACGGTGAATACGTTCTCGGGTCTTGTACACACCGCCCGTCAAACCATGGGAGCTGGTAATGCCCGAAGTCGGTTTATAAACAAACTGCCTAAGGCAGGACTGGTGACTGGGGTTAAGTCGTAACAAGGTATCCCTACGAGAACGTGGGGATGGATCACCTCCTTTCT

>*M. bovis* PG45 (NR_102850.2)

TTTTTCGAGAGTTTGATCCTGGCTCAGGATGAACGCTGGCTGTGTGCCTAATACATGCATGTCGAGCGATGATAGCAATATCATAGCGGCGAATGGGTGAGTAACACGTACTCAACGTACCTTTTAGATTGGGATAGCGGATGGAAACATCCGATAATACCGAATACTTATTATTTTTGCATGAAAGTAATATAAAAGGAAGCGTTTGCTTCGCTAAAAGATCGGAGTGCGCAACATTAGCTAGTTGGTGAGGTAACGGCCCACCAAGGCGATGATGTTTAGCGGGGTTGAGAGATTGATCCGCCACACTGGGACTGAGATACGGCCCAGACTCCTACGGGAGGCAGCAGTAGGGAATATTCCACAATGGACGAAAGTCTGATGGAGCGACACAGCGTGCAGGATGAAGGCCCTATGGGTTGTAAACTGCTGTGGTTAGGGAAGAAAAAGTAGCATAGGAAATGATGCTACCTTGACGGTACCTGATTAGAAAGCAACGGCTAACTATGTGCCAGCAGCCGCGGTAATACATAGGTTGCAAGCGTTATCCGAAATTATTGGGCGTAAAGCGTCTGTAGGTTGTTTGTTAAGTCTGGCGTTAAATTTTGGGGCTCAACCCCAAAACGCGTTGGATACTGGCAGACTAGAGTTATGTAGAGGTTAGCGGAATTCCTTGTGAAGCGGTGAAATGCGTAGATATAAGGAAGAACATCAATATGGCGAAGGCAGCTAACTGGGCATACACTGACACTGAGAGACGAAAGCGTGGGGAGCAAACAGGATTAGATACCCTGGTAGTCCACGCCCTAAACGATGATCATTAGTTGATGGGGAACTCATCGACGCAGCTAACGCATTAAATGATCCGCCTGAGTAGTACGTTCGCAAGAATAAAACTTAAAGGAATTGACGGGGATCCGCACAAGCGGTGGAGCATGTGGTTTAATTTGAAGATACGCGTAGAACCTTACCCACTCTTGACATCTTCTGCAAAGCTATAGAGACATAGTGGAGGTTAACAGAATGACAGATGGTGCATGGTTGTCGTCAGCTCGTGTCGTGAGATGTTCGGTTAAGTCCTGCAACGAGCGCAACCCTTATCCTTAGTTACTACCATTTAGTTGAGCACTCTAAGGAGACTGCCCGAGTAATCGGGAGGAAGGTGGGGACGACGTCAAATCATCATGCCTCTTACGAGTGGGGCTACACACGTGCTACAATGGACGGTACAAAGAGAAGCGAAGTGGTGACATGGAGCAAACCTCAAAAAACCGTTCTCAGTTCGGATTGAAGTCTGCAACTCGACTTCATGAAGTCGGAATCGCTAGTAATCGTAGATCAGCTACGCTACGGTGAATACGTTCTCGGGTCTTGTACACACCGCCCGTCAAACCATGGGAGCTGGTAATGCCCGAAGTCGGTTTATAAAGAAACTGCCTAAGGCAGGACTGGTGACTGGGGTTAAGTCGTAACAAGGTATCCCTACGAGAACGTGGGGATGGATTACCTCCTTT

>*M. agalactiae* 5632 (Molligen)

CTGGCTGTGTGCCTAATACATGCATGTCGAGCGATGATAGCAATATCATAGCGGCGAATGGGTGAGTAACACGTACTCAACGTACCTTTTAGATTGGGATAGCGGATGGAAACATCCGATAATACMGAATACTTATTATTTTTGCATGAAAGTAATATAAAAGGAAGCGYTTGCTTCGCTAGAAGATCGGAGTGCGCAACATTAGCTAGTTGGTGAGGTAACGGCCCACCAAGGCGATGATGTTTAGCGGGGTTGAGAGATTGATCCGCCACACTGGGACTGAGATACGGCCCAGACTCCTACGGGAGGCAGCAGTAGGGAATATTCCACAATGGACGAAAGTCTGATGGAGCGACACAGCGTGCAGGATGAAKGCCCTATGGGTTGTAAACTGCTGTGGTTAGGGAAGAAAAAGTAGCGTAGGAAATGACGCTACCTTGACGGTACCTGATTAGAAAGCAACGGCTAACTATGTGCCAGCAGCCGCGGTAATACATAGGTTGCAAGCGTTATCCGAAATTATTGGGCGTAAAGCGTCTGTAGGTTGTTTGTTAAGTCTGGCGTTAAATTTTGGGGCTCAACCCCAAAACGCGTTGGATACTGGCAGACTAGAGTTATGTAGAGGTTAGCGGAATTCCTTGTGAAGCGGTGAAATGCGTAGATAWAAGGAAGAACATCAATATGGCGAAGGCAGCTAACTGGGCATACACTGACACTGAGAGACGAAAGCGTGGGGAGCAAACAGGATTAGATACCCTGGTAGTCCACGCCCTAAACGATGATCATTAGTTGATGGGGAACTCATCGACGCAGCTAACGCATTAAATGATCCGCCTGAGTAGTACGTTCGCAAGAATAAAACTTAAAGGAATTGACGGGGATCCGCACAAGCGGTGGAGCATGTGGTTTAATTTGAAGATACGCGTAGAACCTTACCCACTCTTGACATCTTCTGCAAAGCTATGGAGACATAGTGGAGGTTAACAGAATGACAGATGGTGCATGGTTGTCGTCAGCTCGTGTCGTGAGAYGTTCGGTTAAGTCCTRCAACGAGCGCAACCCTTATCCTTAGTTACTACCATTTAGTTGAGCACTCTAAGGAGACTGCCCGAGTAATYGGGAGGAAGGTGGGGACGACGTCAAATCATCATGCCTCTTACGAGTGGGGCTACACACGTGCTACAATGGACGGTACAAAGAGAAGCGAAGTGGTGACATGGAGCAAACCTCAAAAAACCGTTCTCAGTTCGGATTGAAGTCTGCAACTCGACTTCATGAAGTCGGAATCGCTAGTAATCGTAGATCAGCTACGCTACGGTGAATACGTTCTCGGGTCTTGTACACACCGCCCGTCAAACCATGGGAGCTGGTAATGCCCGAAGTCGGTTTATTAAGAAACTGCCTAAGGCAGGACTGGTGACTGGGGTTAAGTCGTAACAAGGTATCCCTACGAGAAC

>*Ureaplasma urealyticum* ATCC33699 (Molligen)

ATTAACGCTGGCGGCATGCCTAATACATGCAAATCGAACGAAGCCTTTTAGGCTTAGTGGTGAACGGGTGAGTAACACGTATCCAACCTACCCTTAAGTTGGGGATAACTAGTCGAAAGATTAGCTAATACCGAATAATAACATCAATATCGCATGAGAAGATGTAGAAAGTCGCGTTTGCGACGCTTTTGGATGGGGGTGCGACGTATCAGATAGTTGGTGAGGTAATGGCTCACCAAGTCAATGACGCGTAGCTGTACTGAGAGGTAGAACAGCCACAATGGGACTGAGACACGGCCCATACTCCTACGGGAGGCAGCAGTAGGGAATTTTTCACAATGGGCGCAAGCCTTATGAAGCAATGCCGCGTGAACGATGAAGGTCTTATAGATTGTAAAGTTCTTTTATATGGGAAGAAACGCTAAGATAGGAAATGATTTTAGTTTGACTGTACCATTTGAATAAGTATCGGCTAACTATGTGCCAGCAGCCGCGGTAATACATAGGATGCAAGCGTTATCCGGATTTACTGGGCGTAAAACGAGCGCAGGCGGGTTTGTAAGTTTGGTATTAAATCTAGATGCTTAACGTCTAGCTGTATCAAAAACTGTAAACCTAGAGTGTAGTAGGGAGTTGGGGAACTCCATGTGGAGCGGTAAAATGCGTAGATATATGGAAGAACACCGGTGGCGAAGGCGCCAACTTGGACTATCACTGACGCTTAGGCTCGAAAGTGTGGGGAGCAAATAGGATTAGATACCCTAGTAGTCCACACCGTAAACGATCATCATTAAATGTCGGCTCGAACGAGTCGGTGTTGTAGCTAACGCATTAAATGATGTGCCTGGGTAGTACATTCGCAAGAATGAAACTCAAACGGAATTGACGGGGACCCGCACAAGTGGTGGAGCATGTTGCTTAATTTGACAATACACGTAGAACCTTACCTAGGTTTGACATCTATTGCGACGCTATAGAAATATAGTTGAGGTTAACAATATGACAGGTGGTGCATGGTTGTCGTCAGCTCGTGTCGTGAGATGTTGGGTTAAGTCCCGCAACGAGCGCAACCCCTTTCGTTAGTTGCTTTTCTAGCGATACTGCTACCGCAAGGTAGAGGAAGGTGGGGATGACGTCAAATCATCATGCCCCTTATATCTAGGGCTGCAAACGTGCTACAATGGCTAATACAAACTGCTGCAAAATCGTAAGATGAAGCGAAACAGAAAAAGTTAGTCTCAGTTCGGATAGAGGGCTGCAATTCGCCCTCTTGAAGTTGGAATCACTAGTAATCGCGAATCAGACATGTCGCGGTGAATACGTTCTCGGGTCTTGTACACACCGCCCGTCAAACTATGGGAGCTGGTAATATCTAAAACCGCAAAGCTAACCTTTTGGAGGCATGCGTCTAGGGTAGGATCGGTGACTGGAGTTA

>*M. penetrans* HF-2 (Molligen)

TCAATTTTAAGAGTTTGATCCTGGCTCAGGATTAACGCTAGCGGGATGCCTAATACATGCAAGTCGGACGAAGCACTTGTGCTTAGTGGCGAACGGGTGAGTAACACGTATCTAACATACCTTTTAGTGGGGGATAACTGGTTGAAAAACTAGCTAATACCGCATAGGACATTACTATCGCATGAGAAAATGTTTAAAGTTGCGTTTGCAACGCTTTAAGATTGAGGTGTGGCATATCAGATAGTTGGTAGGGTAACGGCCTACCAAGTCTATGACGTGTAGCTGTGCTGAGAGGCAAAACAGCCACAATGGAACTGAGACACGGTCCATACTTCTACGGGAGGCAGCAGTAGGGAATTTTTCACAATGGGCGCAAGCCTGATGGAGCAATCCCGCGTGAACGACGAAGGTTTTTTAAATTGTAAAGTTCTTTTATTGGGGACGAATTGTAAGAAGAGGAAATGCTTTTTATTTGACTGTACCCTTTGAATAAGTATCGGCTAACTATGTGCCAGCAGCCGCGGTAATACATAGGATGCAAGCGTTATCCGGATTTACTGGGCGTAAAGCGAGCGCAGGCGGATTTACAAGTCTGGTGTTAAATATAGCTGCCCAACAGTTATATGCATTGGAAACTGTAAGTCTAGAGTGCAGTAGAGAGTTTTGGAACTCCATGTGGAGCGGTGGAATGCGTAGATATATGGAAGAACACCAGAGGCGAAGGCGAAAACTTAGGCTGTTACTGACGCTTAGGCTCGAAAGTGTGGGGAGCAAATAGGATTAGATACCCTAGTAGTCCACACCGTAAACGATGGTAATTAAATCTTGGTACGGGATGTATCAGGATTGCAGTTAACACATTAAATTACCCGCCTGGGTAGTACATTCGCAAGAATGAAACTCAAACGGAATTGACGGGGACCCGCACAAGTGGTGGAGCATGTTGCTTAATTCGACGATACACGTAAAACCTTACCTGGGTTTGACATCCTCTGCAAAGCTATAGAGATATAGTGGAGGTTAACAGAGTGACAGGTGGTGCATGGTTGTCGTCAGCTCGTGTCGTGAGATGTTGGGTTAAGTCCCGCAACGAGCGCAACCCTTATCGTTAGTTACTTCATCTAGCGAGACTGCCAGCGTAAGCTGGAGGAAGGTGGGGATGACGTCAAATCATCATGCCCTTTATATCCAGGGCCGCAAACGTGCTACAATGGTCGGTACAAACTGTTGCCAATCAGCAATGAGGAGCTAATCAGAGAAAGCCGATCTCAGTTCGGATCGAGGGCTGCAATTCGTCCTCGTGAAGTTGGAATCACTAGTAATCGCAAATCAGCCATGTTGCGGTGAATACGTTCTCGGGTCTTGTACACACCGCCCGTCAAACTATGAGAGCTGGTAATACCTAAAACCGTTAAGCTAACCTCGTGAGGCTTGCGTCTAGGGTAGGACTGGTAATTGGAGTTAAGTCGTAACAAGGTACCCCTACGGGAATGTGGGGGTGGATCACCTCCTTTCT

>*M. pneumoniae* FH (NR_041751.1)

TTAACGCTGGCGGCATGCCTAATACATGCAAGTCGATCGAAAGTAGTAATACTTTAGAGGCGAACGGGTGAGTAACACGTATCCAATCTACCTTATAATGGGGGATAACTAGTTGAAAGACTAGCTAATACCGCATAAGAACTTTGGTTCGCATGAATCAAAGTTGAAAGGACCTGCAAGGGTTCGTTATTTGATGAGGGTGCGCCATATCAGCTAGTTGGTGGGGTAACGGCCTACCAAGGCAATGACGTGTAGCTATGCTGAGAAGTAGAATAGCCACAATGGGACTGAGACACGGCCCATACTCCTACGGGAGGCAGCAGTAGGGAATTTTTCACAATGAGCGAAAGCTTGATGGAGCAATGCCGCGTGAACGATGAAGGTCTTTAAGATTGTAAAGTTCTTTTATTTGGGAAGAATGACTTTAGCAGGTAATGGCTAGAGTTTGACTGTACCATTTTGAATAAGTGACGACTAACTATGTGCCAGCAGTCGCGGTAATACATAGGTCGCAAGCGTTATCCGGATTTATTGGGCGTAAAGCAAGCGCAGGCGGATTGAAAAGTCTGGTGTTAAAGGCAGCTGCTTAACAGTTGTATGCATTGGAAACTATTAATCTAGAGTGTGGTAGGGAGTTTTGGAATTTCATGTGGAGCGGTGAAATGCGTAGATATATGAAGGAACACCAGTGGCGAAGGCGAAAACTTAGGCCATTACTGACGCTTAGGCTTGAAAGTGTGGGGAGCAAATAGGATTAGATACCCTAGTAGTCCACACCGTAAACGATAGATACTAGCTGTCGGGGCGATCCCCTCGGTAGTGAAGTTAACACATTAAGTATCTCGCCTGGGTAGTACATTCGCAAGAATGAAACTCAAACGGAATTGACGGGGACCCGCACAAGTGGTGGAGCATGTTGCTTAATTCGACGGTACACGAAAAACCTTACCTAGACTTGACATCCTTGGCAAAGTTATGGAAACATAATGGAGGTTAACCGAGTGACAGGTGGTGCATGGTTGTCGTCAGCTCGTGTCGTGAGATGTTGGGTTAAGTCCCGCAACGAGCGCAACCCTTATCGTTAGTTACATTGTCTAGCGAGACTGCTAATGCAAATTGGAGGAAGGAAGGGATGACGTCAAATCATCATGCCCCTTATGTCTAGGGCTGCAAACGTGCTACAATGGCCAATACAAACAGTCGCCAGCTTGTAAAAGTGAGCAAATCTGTAAAGTTGGTCTCAGTTCGGATTGAGGGCTGCAATTCGTCCTCATGAAGTCGGAATCACTAGTAATCGCGAATCAGCTATGTCGCGGTGAATACGTTCTCGGGTCTTGTACACACCGCCCGTCAAACTATGAAAGCTGGTAATATTTAAAAACGTGTTGCTAACCATTAGGAAGCGCATGTCAAGGATAGCACCGGTGATTGGAGTTAAGTCGTAACAAGGTACCCCTACGAGAACGTGGGGGTGGATCACCTCCTTT

>*M. genitalium* G37 (NR_074611.1)

CAATTTTTTCTGAGAGTTTGATCCTGGCTCAGGATTAACGCTGGCGGCATGCCTAATACATGCAAGTCGATCGGAAGTAGCAATACTTTAGAGGCGAACGGGTGAGTAACACGTATCCAATCTACCTTATAATGGGGGATAACTAGTTGAAAAACTAGCTAATACCGCATAAGAACTTTAGTTCGCATGAATTAAAGTTGAAAGGACCTGCAAGGGTTCGTTATTTGATGAGGGTGCGCCATATCAGCTAGTTGGTAGGGTAATGGCCTACCAAGGCAATGACGTGTAGCTATGCTGAGAAGTAGAATAGCCACAATGGGACTGAGACACGGCCCATACTCCTACGGGAGGCAGCAGTAGGGAATTTTTCACAATGAGCGAAAGCTTGATGGAGCAATGCCGCGTGAACGATGAAGGTCTTTTTGATTGTAAAGTTCTTTTATTTGGGAAGAATGACTCTAGCAGGCAATGGCTGGAGTTTGACTGTACCACTTTGAATAAGTGACGACTAACTATGTGCCAGCAGTCGCGGTAATACATAGGTCGCAAGCGTTATCCGGATTTATTGGGCGTAAAGCAAGCGCAGGCGGATTGAAAAGTCTGGTGTTAAAGGCAGCTGCTTAACAGTTGTATGCATTGGAAACTATCAGTCTAGAGTGTGGTAGGGAGTTTTGGAATTTCATGTGGAGCGGTGAAATGCGTAGATATATGAAGGAACACCAGTGGCGAAGGCGAAAACTTAGGCCATTACTGACGCTTAGGCTTGAAAGTGTGGGGAGCAAATAGGATTAGATACCCTAGTAGTCCACACCGTAAACGATAGATACTAGCTGTCGGAGCGATCCCTTCGGTAGTGAAGTTAACACATTAAGTATCTCGCCTGGGTAGTACATTCGCAAGAATGAAACTCAAACGGAATTGACGGGGACCCGCACAAGTGGTGGAGCATGTTGCTTAATTCGACGGTACACGAAAAACCTTACCTAGACTTGACATCCTTGGCAAAGTTATGGAAACATAATGGAGGTTAACCGAGTGACAGGTGGTGCATGGTTGTCGTCAGCTCGTGTCGTGAGATGTTGGGTTAAGTCCCGCAACGAGCGCAACCCTTATCGTTAGTTACATTGTTTAACGAGACTGCTAATGTAAATTGGAGGAAGGAAGGGATGACGTCAAATCATCATGCCCCTTATGTCTAGGGCTGCAAACGTGCTACAATGGCCAATACAAACAGTAGCCAACTTGTAAAAGTGAGCAAATCTGAAAAGTTGGTCTCAGTTCGGATTGAGGGCTGCAATTCGTCCTCATGAAGCTGGAATCACTAGTAATCGCGAATCAGCTATGTCGCGGTGAATACGTTCTCGGGTCTTGTACACACCGCCCGTCAAACTATGAAAGCTGGTAATATTTAAAAACGTGTTGCTAACCTTTATTGGAAGCGCATGTCAAGGATAGCACCGGTGATTGGAGTTAAGTCGTAACAAGGTACCCCTACGAGAACGTGGGGGTGGATCACCTC

>*M. gallisepticum* PG31 (NR_104952.1)

TCATGGCTCAGGATTAACGCTGGCGGCATGCCTAATACATGCAAGTCGATCGGATGTAGCAATACATTAGAGGCGAACGGGTGAGTAACACGTATCCAATCTGCCTTATAGTGGGGGATAACTAGTCGAAAGATTAGCTAATACCGCATAACAAGTTAACTATCGCATGAGAATAACTTTAAAGAAGCAACTGCTTCGCTATAAGATGAGGGTGCGGCATATCAGCTAGTTGGTGAGGGTAATGGCCCACCAAGGCGATGACGTGTAGTTATGCTGAGAGGTAGAATAACCACAATGGGACTGAGACACGGCCCATACTCCTACGGGAGGCAGCAGTAGGGAATTTTTCACAATGGACGAAAGTCTGATGGAGCAATGCCGCGTGAACGATGAAGGTCTTTTTAGATTGTAAAGTTCTTTTATTTGGGAAGAACAGTTAATAGAGTGGAAAGCTATTAATTTGACTGTACCATTTGAATAAGTAACGACTAACTATGTGCCAGCAGTCGCGGTAATACATAGGTTGCAAGCGTTATCCGGATTTATTGGGCGTAAAACAAGCGCAGGCGGATTAGAAAGTCTGGTGTTAAAAGCAATTGCTTAACGATTGTATGCATTGGAAACTTCTAGTCTAGAGTTTGGTAGAGAGTCCTGGAACTCCATGTGGAGCGGTGAAATGCGTAGATATATGGAAGAACACCAGAGGCGAAGGCGAGGACTTGGGCCAATACTGACGCTTAGGCTTGAAAGTGTGGGGAGCAAATAGGATTAGATACCCTAGTAGTCCACACTGTAAACGATGGATGTTAAGTGTCGGAGCGAATACTTCGGTGCTGCAGTTAACACATTAAACATCCTGCCTGAGTAGTACATTCGCAAGAATGAAACTCAAACGGAATTGACGGGGACCCGCACAAGTGGTGGAGCATGTTGCTTAATTCGACGGTACACGAAAAACCTTACCTAGACTTGACATCTTGGGCGAAGCTATAGAAATATAGTGGAGGTCAACCCAATGACAGGTGGTGCATGGTTGTCGTCAGCTCGTGTCGTGAGATGTTGGGTTAAGTCCCGCAACGAGCGCAACCCTTATCGTTAGTTACTTTGTCTGACGAGACTGCCAACGTAAGTTGGAGGAAGGTGGGGATGACGTCAAATCATCATGCCCCTTATGTCTAGGGCTGCAAACGTGCTACAATGGCCAATACAAACAGTTGCAAATCCGTAAGGTGGAGCTAATCTGTAAAGTTGGTCTCAGTTCGGATTGAGGGCTGCAATTCGCCCTCATGAAGTCGGAATCACTAGTAATCGCGAATCAGCCATGTCGCGGTGAATACGTTCTCGGGTCTTGTACACACCGCCCGTCAAACTATGAGAGCTGGTAATATCTAAAACCGTGTTGCTAACCGCAAGGAAGCGCATGTCTAGGGTAGGGCCGGTGATTGGAGTTAAGTCGTA

>*Acholeplasma laidlawii* PG-8A (NR_025961.1)

AGAGTTTGATCCTGGCTCAGGATGAACGCTGGCGGCGTGCCTAATACATGCAAGTCGAACGAAGCATCTTCGGATGCTTAGTGGCGAACGGGTGAGTAACACGTAGATAACCTACCTTTAACTCGAGGATAACTCCGGGAAACTGGAGCTAATACTGGATAGGATGTGTGCATGAAAAAAACACATTTAAAGATTTATCGGTTTAAGAGGGGTCTGCGGCGCATTAGTTAGTTGGTGGAGTAAAAGCCTACCAAGACGATGATGCGTAGCCGGACTGAGAGGTCTACCGGCCCACATTGGGACTGAGAACGGCCCAAACTCCTACGGGAGGCAGCAGTAAGGGAATTTTCGGCAATGGGGGAAACCCTGACCGACCAACGCCGCGTGAACGACGAAGTACTTCGGTATGTAAAGTTCTTTTATATGGGAAGAAAAATTGAAAATTGACGGTACCATATGAATAAGCCCCGGCTAACTATGTGCCAGCAGCCGCGGTAATACATAGGGGGCGAGCGTTATCCGGATTTACTGGGCGTAAAGGGTGCGTAGGTGGTTATAAAAGTTTGTGGTGTAAGTGCAGTGCTTAACGCTGTGAGGCTATGAAAACTATATAACTAGAGTGAGACAGAGGCAAGTGGAATTCCATGTGTAGCGGTAAAATGCGTAAATATATGGAGGAACACCAGTGGTGAAGGCGGCTTGCTGGGTCTATACTGACACTGATGCACGAAAGCGTGGGGAGCAAACAGGATTAGATACCCTGGTAGTCCACGCCGTAAACGATGAGAACTAAGTGTTGGGCAAAAGGTCAGTGCTGCAGTTAACGCATTAAGTTCTCCGCCTGAGTAGTACGTACGCAAGTATGAAACTCAAAGGAATTGACGGGACCCCGCACAAGCGGTGGGATCATGTTGTTTAATTCGAAGATACAACGAAAAACCTTACCAGGTCTTTGACATACTCTGCAAAAGGCTTAGAAATAAGTCCGGAGGCTTACAGATGTACAGGTGGTGCACGGTTGTCGTCAGCTCGTGTCGTGAGATGTTGGGTTAAGTCCCGCAACGAGCGCAACCCTTATTGCTAGTTACCATCATTAAGTTGGGGACTCTAGCGAGACTGCCAGTGATAAATTGGAGGAAGGTGGGGATGACGTCAAATCATCATGCCCCTTATGAACCTGGGCTACCAAACGTGATACAATGGCTGGAACAAAGAGAAGCGATAGGGTGACCTGGAGCGAAACTCACAAAAACAGTCTCAGTTCGGATTGGAGTCTGCAACTCGACTCCATGAAGTCGGAATCGCTAGTAATCCCAAATCAGCATGTTGCGGTGAATACGTTCTCGGGGTTTGTACACACCGCCCGTCAAACCACGAAAGTGGGCAATACCCAACGCCGGTGGCCTAACCCGAAAGGGAGGGAGCCGTCTAAGGTAGGGTCCATGATTGGGGTTAAGTCGTAACAAGGTAACCCTA

>*Bacillus subtilis* 168 (NR_102783.2)

TTATCGGAGAGTTTGATCCTGGCTCAGGACGAACGCTGGCGGCGTGCCTAATACATGCAAGTCGAGCGGACAGATGGGAGCTTGCTCCCTGATGTTAGCGGCGGACGGGTGAGTAACACGTGGGTAACCTGCCTGTAAGACTGGGATAACTCCGGGAAACCGGGGCTAATACCGGATGGTTGTTTGAACCGCATGGTTCAAACATAAAAGGTGGCTTCGGCTACCACTTACAGATGGACCCGCGGCGCATTAGCTAGTTGGTGAGGTAACGGCTCACCAAGGCGACGATGCGTAGCCGACCTGAGAGGGTGATCGGCCACACTGGGACTGAGACACGGCCCAGACTCCTACGGGAGGCAGCAGTAGGGAATCTTCCGCAATGGACGAAAGTCTGACGGAGCAACGCCGCGTGAGTGATGAAGGTTTTCGGATCGTAAAGCTCTGTTGTTAGGGAAGAACAAGTGCCGTTCGAATAGGGCGGTACCTTGACGGTACCTAACCAGAAAGCCACGGCTAACTACGTGCCAGCAGCCGCGGTAATACGTAGGTGGCAAGCGTTGTCCGGAATTATTGGGCGTAAAGGGCTCGCAGGCGGTTTCTTAAGTCTGATGTGAAAGCCCCCGGCTCAACCGGGGAGGGTCATTGGAAACTGGGGAACTTGAGTGCAGAAGAGGAGAGTGGAATTCCACGTGTAGCGGTGAAATGCGTAGAGATGTGGAGGAACACCAGTGGCGAAGGCGACTCTCTGGTCTGTAACTGACGCTGAGGAGCGAAAGCGTGGGGAGCGAACAGGATTAGATACCCTGGTAGTCCACGCCGTAAACGATGAGTGCTAAGTGTTAGGGGGTTTCCGCCCCTTAGTGCTGCAGCTAACGCATTAAGCACTCCGCCTGGGGAGTACGGTCGCAAGACTGAAACTCAAAGGAATTGACGGGGGCCCGCACAAGCGGTGGAGCATGTGGTTTAATTCGAAGCAACGCGAAGAACCTTACCAGGTCTTGACATCCTCTGACAATCCTAGAGATAGGACGTCCCCTTCGGGGGCAGAGTGACAGGTGGTGCATGGTTGTCGTCAGCTCGTGTCGTGAGATGTTGGGTTAAGTCCCGCAACGAGCGCAACCCTTGATCTTAGTTGCCAGCATTCAGTTGGGCACTCTAAGGTGACTGCCGGTGACAAACCGGAGGAAGGTGGGGATGACGTCAAATCATCATGCCCCTTATGACCTGGGCTACACACGTGCTACAATGGACAGAACAAAGGGCAGCGAAACCGCGAGGTTAAGCCAATCCCACAAATCTGTTCTCAGTTCGGATCGCAGTCTGCAACTCGACTGCGTGAAGCTGGAATCGCTAGTAATCGCGGATCAGCATGCCGCGGTGAATACGTTCCCGGGCCTTGTACACACCGCCCGTCACACCACGAGAGTTTGTAACACCCGAAGTCGGTGAGGTAACCTTTTAGGAGCCAGCCGCCGAAGGTGGGACAGATGATTGGGGTGAAGTCGTAACAAGGTAGCCGTATCGGAAGGTGCGGCTGGATCACCTCCTTT

>Candidatus Phytoplasma mali (ATP_R0001)

TTTATATTAAGAGTTTGATCCTGGCTCAGGATGAACGCTGGCGGCGTGCCTAATACATGCAAGTCGAACGGAAACTTTTAGTTTCAGTGGCGAACGGGTGAGTAACACGTAAGTAACCTGCCTCTTAGACGAGGATAACAGTTGGAAACGACTGCTAAGACTGGATAGGAAGTTTTAAGGCATCTTGAAACTTTTAAAAGACCCGCAAGGGTATGCTAAGAGATGGGCTTGCGGCACATTAGTTAGTTGGTAAGGTAACGGCTTACCAAGACTATGATGTGTAGCTGGACTGAGAGGTTGAACGGCCACATTGGGATTGAGATACGGCCCAAACTCCTACGGGAGGCAGCAGTAAGGAATTTTCGGCAATGGAGGAAACTCTGACCGAGCAACGCCGCGTGAACGATGAAATATTTAGGTATGTAAAGTTCTTTTATTAAAGAAGAAAAAATGATGGAAAAATCATTCTGACGGTATTTAATGAATAAGCCCCGGCTAACTATGTGCCAGCAGCTGCGGTAATACATGGGGGGCAAGCGTTATCCGGATTTATTGGGCGTAAAGGGTGTGTAGGCGGTTTAATAAGTCTATGGTATAAGTTCAACGCTTAACGTTGTGATGCTATGGAAACTGTTTGACTAGAGTTGGATAGAGGCAAGTGGAATTCCATGTGTAGCGGTAAAATGCGTAAATATATGGAGGAACACCAGTAGCGAAGGCGGCTTGCTGGGTCTTAACTGACGCTGAGGCACGAAAGCGTGGGGAGCAAACAGGATTAGATACCCTGGTAGTCCACGCCGTAAACGATGAGTACTAAGTGTTGGGTTAAACCAGTGCTGAAGTTAACACATTAAGTACTCCGCCTGAGTAGTACGTACGCAAGTATGAAACTTAAAGGAATTGACGGGACTCCGCACAAGCGGTGGATCATGTTGTTTAATTCGAAGATACACGAAAAACCTTACCAGGTCTTGACATACTCTGCAAAGCTATAGAAATATAGTGGAGGTTATCAGGGATACAGGTGGTGCATGGTTGTCGTCAGCTCGTGTCGTGAGATGTTGGGTTAAGTCCCGCAACGAGCGCAACCCTTATCGCTAGTTACCATCATTTAGTTGGGCACTTTAGTGAGACTGCCAATGATAAATTGGAGGAAGGTGGGGATTACGTCAAATCATCATGCCCCTTATGACCTGGGCTACAAACGTGATACAATGGCTGTTACAAAGAGTAGCTGAAGCGTGAGTTTTTAGCAAATCTCAAAAAAACAGTCTCAGTTCGGATTGAAGTCTGCAACTCGACTTCATGAAGTCGGAATCGCTAGTAATCGCGAATCAGCATGTCGTGGTGAATACGTTCTCGGGGTTTGTACACACCGCCCGTCAAACCATGAAAGTTGACAATACTCGAAACCAGTAGCCTAACTTGCAAAAGAGGGAACTGTCTAAGGTAGGGTCGATGATTGGGGTTAAGTCGTAACAAGGTATCCCTACCGGAAGGTGGGGATGGATCACCTCCTTTCT

>*E. coli* MG1655

AAATTGAAGAGTTTGATCATGGCTCAGATTGAACGCTGGCGGCAGGCCTAACACATGCAAGTCGAACGGTAACAGGAAGAAGCTTGCTTCTTTGCTGACGAGTGGCGGACGGGTGAGTAATGTCTGGGAAACTGCCTGATGGAGGGGGATAACTACTGGAAACGGTAGCTAATACCGCATAACGTCGCAAGACCAAAGAGGGGGACCTTCGGGCCTCTTGCCATCGGATGTGCCCAGATGGGATTAGCTAGTAGGTGGGGTAACGGCTCACCTAGGCGACGATCCCTAGCTGGTCTGAGAGGATGACCAGCCACACTGGAACTGAGACACGGTCCAGACTCCTACGGGAGGCAGCAGTGGGGAATATTGCACAATGGGCGCAAGCCTGATGCAGCCATGCCGCGTGTATGAAGAAGGCCTTCGGGTTGTAAAGTACTTTCAGCGGGGAGGAAGGGAGTAAAGTTAATACCTTTGCTCATTGACGTTACCCGCAGAAGAAGCACCGGCTAACTCCGTGCCAGCAGCCGCGGTAATACGGAGGGTGCAAGCGTTAATCGGAATTACTGGGCGTAAAGCGCACGCAGGCGGTTTGTTAAGTCAGATGTGAAATCCCCGGGCTCAACCTGGGAACTGCATCTGATACTGGCAAGCTTGAGTCTCGTAGAGGGGGGTAGAATTCCAGGTGTAGCGGTGAAATGCGTAGAGATCTGGAGGAATACCGGTGGCGAAGGCGGCCCCCTGGACGAAGACTGACGCTCAGGTGCGAAAGCGTGGGGAGCAAACAGGATTAGATACCCTGGTAGTCCACGCCGTAAACGATGTCGACTTGGAGGTTGTGCCCTTGAGGCGTGGCTTCCGGAGCTAACGCGTTAAGTCGACCGCCTGGGGAGTACGGCCGCAAGGTTAAAACTCAAATGAATTGACGGGGGCCCGCACAAGCGGTGGAGCATGTGGTTTAATTCGATGCAACGCGAAGAACCTTACCTGGTCTTGACATCCACAGAACTTTCCAGAGATGGATTGGTGCCTTCGGGAACTGTGAGACAGGTGCTGCATGGCTGTCGTCAGCTCGTGTTGTGAAATGTTGGGTTAAGTCCCGCAACGAGCGCAACCCTTATCTTTTGTTGCCAGCGGTCCGGCCGGGAACTCAAAGGAGACTGCCAGTGATAAACTGGAGGAAGGTGGGGATGACGTCAAGTCATCATGGCCCTTACGACCAGGGCTACACACGTGCTACAATGGCGCATACAAAGAGAAGCGACCTCGCGAGAGCAAGCGGACCTCATAAAGTGCGTCGTAGTCCGGATTGGAGTCTGCAACTCGACTCCATGAAGTCGGAATCGCTAGTAATCGTGGATCAGAATGCCACGGTGAATACGTTCCCGGGCCTTGTACACACCGCCCGTCACACCATGGGAGTGGGTTGCAAAAGAAGTAGGTAGCTTAACCTTCGGGAGGGCGCTTACCACTTTGTGATTCATGACTGGGGTGAAGTCGTAACAAGGTAACCGTAGGGGAACCTGCGGTTGGATCACCTCCTTA

>*Clostridium botulinum* C (FN552458.1)

TTCGGGGAGTGGATTAGCGGCGGACGGGTGAGTAACACGTGGGTAACCTGCCTCAAAGAGGGGGATAGCCTCCCGAAAGGGAGATTAATACCGCATAACATTATTTTATGGCATCATAGAATAATCAAAGGAGCAATCCGCTTTGAGATGGACCCGCGGCGCATTAGCTAGTTGGTGAGGTAAGGGCTCACCAAGGCGACGATGCGTAGCCGACCTGAGAGGGTGATCGGCCACATTGGAACTGAGACACGGTCCAGACTCCTACGGGAGGCAGCAGTGGGGAATATTGCGCAATGGGGGAAACCCTGACGCAGCAACGCCGCGTGAGTGATGAAGGTTTTCGGATCGTAAAACTCTGTCTTTAGGGACGATAATGACGGTACCTAAGGAGGAAGCCACGGCTAACTACGTGCCAGCAGCCGCGGTAATACGTAGGTGGCAAGCGTTGTCCGGATTTACTGGGCGTAAAGAGTATGTAGGTGGGTGCTTAAGTCAGATGTGAAATTCCCGGGCTTAACCTGGGCGCTGCATTTGAAACTGGGCATCTAGAGTGCAGGAGAGGAAAGTGGAATTCCTAGTGTAGCGGTGAAATGCGTAGAGATTAGGAAGAACACCAGTGGCGAAGGCGACTTTCTGGACTGTAACTGACACTGAGATACGAAAGCGTGGGTAGCAAACAGGATTAGATACCCTGGTAGTCCACGCCGTAAACGATGAATACTAGGTGTCGGGGGGTACCACCCTCGGTGCCGCAGCAAACGCATTAAGTATTCCGCCTGGGGAGTACGGTCGCAAGATTAAAACTCAAAGGAATTGACGGGGACCCGCACAAGCAGCGGAGCATGTGGTTTAATTCGAAGCAACGCGAAGAACCTTACCTAGACTTGACATCTCCTGAATTACTCTTAATCGAGGAAGTCCCTTCGGGGACAGGAAGACAGGTGGTGCATGGTTGTCGTCAGCTCGTGTCGTGAGATGTTGGGTTAAGTCCCGCAAACGAGCGCAACCCTTATTGTTAGTTGCTACTATTAAGTTAAGCACTCTAACGAGACTGCCGCGGTTAACGTGGAGGAAGGTGGGGATGACGTCAAATCATCATGCCCCTTATGTCTAGGGCTACACACGTGCTACAATGGCTGGTACAACGAGCAGCAAACCCGCGAGGGGGAGCAAAACTTGAAAGCCAGTCCCAGTTCGGATTGTAGGCTGAAACTCGCCTACATGAAGTTGGAGTTGCTAGTAATCGCGAATCAGCATGTCGCGGTGAATACGTTCCCGGGTCTTGTACACACCGCCCGTCACACCATGAGAGCCGGTAACACCCGAAGCCCGTGAGGTAACCGTAAGGAGCCAGCGGTCGAAGGTGGGATTGGT
