# Supplementary material for: Minimalistic mycoplasmas harbor different functional toxin-antitoxin systems
Source: PLoS Genet. 2021 Oct 21;17(10):e1009365. doi: 10.1371/journal.pgen.1009365 (PMC8562856; doi:10.1371/journal.pgen.1009365)
Supplement: S6 File — (DOCX) [file pgen.1009365.s015.docx]

***E. coli* codon-optimized sequences of TA elements for heterologous expression in *E. coli* and *B. subtilis* (restriction sites are colour coded and the ATG start codon is underlined)**

*NdeI* restriction site

*SacI* restriction site

*EcoRI* restriction site

*BamHI* restriction site

**A_132_**

ATCTACCATATGAAGAAAGCGAACGTGCTGAACCTGATTCGCTACCACATCGAGGAGAACGACATTAGCTTCCGTAAAGAGGCGCGTATTATTGCGGAGGAGTTCTACAAAATGGGTGACGATGAGCTGGCGGAATATGTGCTGTTCATGCTGCGTGATGCGAACCACTTTGTTCCGCAGATTGATCAAGAGTACGACATTCAGATCCCGTTTACCCAAAAAATCGAGCTGGAACGTAACAGCGAACCGCTGCCGCTGCCGCAAGTGATTAGCGAGGAAATCAAGGGTGTTATTAACGCGATCAGCAAGAACCGTAAAATCAACAAGTTCCTGTTTCAGGGCTTCCCGGGTACCGGCAAAACCGAAACCGTGAAGCAAATTGCGCGTATCCTGAACCGTAACCTGTTCATGGTTGATTTTAACAACCTGATTGACAGCCACCTGGGCCAGAGCAGCAAAAACATTGCGGAACTGTTCCAGAAGATCAACCAAACCCCGAACCCGAAGAAAATCATTATCTGCTTTGACGAGATCGATGCGCTGGCGCTGGATCGTACCAACAAAACCGACCTGCGTGAAATGGGTCGTGTGACCACCGCGGTTTTCCAAGGCCTGGATAAGCTGGACACCGATATTATCGTGTTTGCGACCACCAACCTGTTCAAACACTTTGACAAGGCGCTGATTCGTCGTTTCGATCTGGTTATCGACTTTAACCGTTACACCAAGAAAGACATGCTGGATATCGCGGAGATTATCCTGAAGCACTACATTAAGAAAGTGGATAACATCAAGAGCGAACTGCGTCTGTTCCGTAAGATTATCAGCCTGAGCGAGGAACTGATTTACCCGGGTGACCTGAAAAACATTATCAAGAGCAGCATCTACCTGAGCGACTATGAGGATCAGTACGACTACCTGAAGCGTATCTACAAGAAAATCACCGACGATAAGCTGGACATCCGTCAACTGAACGAAAACAACTTTACCGTTCGTGAGATTGAAATCCTGAAGGGCCTGAGCAAAAGCAGCGTGGCGCTGAAGGTGAAAGAACTGAATAGCAATGAGTAAGGATCCAGACTT

**T_133_**

ATGATCGAGCTCATGAACAACCTGATCGTGCTGAAGGGTAAATTTGAGCCGGGCAAGAACACCAAGAAACCGAACAGCCCGCAGATCCCGAAAACCAGCATCATTAAGCTGGAGGATTGCTACCGTATCCTGGACCAGCTGATTAAGGCGAGCAGCTTCTGGAAGGAACAAAAGATCGATATCAACCCGATCATCAACGTTAAGTACAAGCGTATCATCAGCAAGAGCAACCGTGTGAGCTACCTGCTGCTGAAAAGCCTGCAGAAGAACAACGAACACATCATTGGTAGCAGCTTTCTGGACGAGCTGGTTGAAAAGAAAATCGTGAAGAAACAAGTTATCACCTACTGCCTGACCCAGAAGGATCTGCAAGAGGCGATCAAACGTCTGGACACCATCACCAACATTCTGAAGAAAACCCACTTCAAGCGTATTGACAACAACCTGATCAACCTGATTGCGAACGAACAGTATCTGCCGATCAAGAAAGAGATTCAAAAATACGAGTTCCTGAGCCGTACCGCGTTTATCAGCACCCTGGTGGATCTGAACTACATCGAGGAAATCTTCATCAAGACCACCCACATCGACAACAACGTTGATAGCGTGGTTACCCTGTACGACACCGGTATCAAGGCGATTGATCTGCTGAACAAACTGGACATCAACGTGAACATGAGCGATTTTATTGACGATTATACCCTGTTCCTGGACCGTAACCAGTACAACGAGCTGAAGACCAAAGCGCCGTTTCTGATCAGCATGAGCGTTGACGATCTGACCAAATTCATCATTGACGATAAGCAAGAGGAAATCACCAAAAACGATATCATTAGCATTCCGGACCCGACCAACGAGCCGATTGTGGGCGTTATTGATACCATGTTCTGCAAGGACGTGTATTTTAGCAAATGGGTTGACTTCCGTAAGGAAGTGAGCGACGATATCCTGCTGGACAGCAAAGATTACCAGCACGGTACCCAAGTGAGCAGCATCATTGTTGATGGCCCGAGCTTTAACAAGAAACTGGAGGACGGTTGCGGCCGTTTCCGTGTGCGTCACTTTGGTGTTATGGCGCACAGCAGCGGCAACGTGTTCAGCCTGTTCAAGAAAATCAAGAGCATCGTTATCAACAACCTGGATATTAAAGTGTGGAACCTGAGCCTGGGTAGCATCCGTGAAGTTAGCAGCAACTATATTAGCCTGCTGGGCAGCCTGCTGGATCAGCTGCAATACGAGAACGACGTGATCTTCATTGTTGCGGGTACCAACGACAACGAATGCAAGCAGAAAATCGTTGGCAGCCCGGCGGATAGCATCAACAGCATTGTGGTTAACAGCGTGGACTTTAAGAACAAACCGGCGAACTATAGCCGTAAGGGTCCGGTTCTGACCTACTTCAACAAACCGGATATCAGCTACTATGGTGGCGTTGACAACAACAAGATTACCGTGTGCGGTTGCTATGGCGAAGCGAAAGTTCAAGGCACCAGCTTTGCGGCGCCGTGGATCACCCGTAAGGTGGCGTACCTGATCTACAAGATGAACTACAGCAAAGAGGAAGCGAAGGCGCTGATCATTGATAGCGCGATCAAATTCGACAAGCAGAAAGATAACAACCGTGACCTGATTGGTTACGGCGTGGTTCCGATCCACATTAACGAGATCCTGCAAAGCAAAAACACCGACATTAAGGTGCTGCTGAGCTATAACACCAAGGCGTACTATACCTACAACTTTAACCTGCCGGTTCCGACCAAGGAAAACAAATTCCCGTTTATCGCGAAACTGACCTTCGCGTATTTTGCGGAGAGCCAGCGTAGCCAAGGTGTGGACTACACCCAGGACGAACTGGATATCCAATTCGGCCCGATTGATAACAAGAGCGAGAGCATCAACGACATTAACGAAAACAACCAGAGCAGCAGCAGCAGCAACGCGTACATCTATGAGTACGAAGCGCGTAAAATGTTTGCGAAGTGGAACACCGTTAAAAGCATCATTAAGTGGAGCAAAACCAACAAGGGTAAGAAACGTCAATTCATCAAGACCACCAACAACCGTTGGGGCATCCGTGTGATTCGTAAAACCCGTACCGATAACATCAACAACAAGAGCATCAAGTTCAGCCTGGTTATCACCTTCCGTAGCATTGACAACAAGGATCGTATCGAGGAATTTATTAGCCTGTGCAACAAAAGCGGTTATTGGGTGGCGAGCAAAGTTCAGATCGATAACAAGATCGACATTCACGGCAAAAGCAACGAGTACCTGGACTTCGAATAAGAATTCTAGACT

**T_752_**

ATGATCGAGCTCATGAAAGCGAACTTCCTGGAGGAAGAGTTTGAAATCAACCTGAGCGTTAAGCACCTGCTGGAGCTGTGGGACAAAAACCTGCTGAACACCTTCGAAATTGGTACCTTTAAAGGCCTGAGCCAGATCCACAGCTACATGTTCAAGGACATTTTCGATTTTAACGGTCAAATCCGTAACGTTAACATTAGCAAGAACAACAGCATGTTTTGCCTGGCGCGTTACCTGAAGCAGAACCTGGAGATCATTGACAACATGAAACACGACACCTTCGATCAAATCATCGATAAGTACGTGGAAATGAACATCTGCCACCCGTTTCGTGAGGGTAACGGCCGTAGCATGCGTATCTGGCTGGACCTGATTCTGAAGAAACAGCTGAACGTGGTTGTGAACTGGACCAACATCAACAAAGACGAATACCTGCTGGCGATGATCAACAGCCTGATTGATAGCACCAACCTGAAGCTGCTGATTAAAAACAACCTGACCAACAAGATCACCGATCGTAACGTGTACATCAAGAGCATCATCAAGAGCTACGAATATGAGGGCTTCAAGATCAACATTAAATAAGAATTCTAGACT

**A_753_**

ATCTACCATATGAAGAAGGTTAATGTGAATATCAAAATGGACCCGGAAATCAAGAAACAAGCGAGCCTGCTGTTCAAAGAGTTCGGTATGACCATGAGCAGCGCGATCAACCTGTTCGTGAAGACCGCGGTTGAGCAGAACGACATCCCGTTTGAGTACAACCGTGAAATTACCAACAAGGAAACCCTGGAAGCGTTCAAAGAGGGTGAACGTCTGCTGGCGGATAAAAATGCGAAACGCTACAGCAGCTTCAAGGAGATTCTGGACAGCCTGGACAAATAAGGATCCAGACTT

**T_160_**

GAGCTCATGATCAGCATTCTGGAGAAAATCGTGAAGAGCAAAAACAAGCAGAACTTCATCATTAAGGGTGGCTTTCTGCTGAGCAACATGATGAGCCTGAACGAGCGTACCACCCAAGACATTGATTGCCTGATCAAAGGTATTAACTTCGTGAAAGAAAACATCCTGAAGATCATTAGCGACGTTCTGCGTTACGACTTCACCGATTTCATCGAGTACGAAATCAAGGAGATCACCGAAATCAAGAAACGTGATAAATACAACGGTTTTCGTGTTAAGATCCTGTGCAAACTGGAAAAGCTGGAGGAAATTGTGAAAATCGACCTGGCGATTGGCGATGTTATCACCCCGAGCGAGATCAAGTACAACTTCAAGAACATCTTTAGCAACAGCAGCTTCGAAATCCAGGCGTATAACCTGGAGACCATTCTGGCGGAAAAGATCTTCATCATCAACAACCTGAACATTCAAAGCACCCGTATCAAGGACCTGTACGATATTTTCCTGATCTATAGCCTGAAAGAGTTTGAAATTGACTACAACATCCTGAAGTGCGCGTGCCTGAACACCTTCAAGAAACGTAACACCGTGTTTAACATTGACCTGATCCTGAACCTGCTGAACGAGATTAACCAGCTGGATATCTTCAAGCTGCTGTGGAACAACTATAAACAGAAGTACTTCTATGCGCAAAAAATTGAGTTTGAAATCCTGGTGGAGAGCGTTATCAAGCTGCTGGAAAAACTGAAGAGCAGCTAAGAATTC

**A_161_**

ATGATCCATATGAACTATAAGGAGCAGCTGATTAAAGAAGCGAAGAAACGTGACGGTCTGATCACCCGTAAGGAGATCATTAACCTGAAAATTCCGAGCATCTATATTACCCGTATGATCCGTAGCAACGAGCTGGAAAAGGTGGATATCGGCATTTACGCGCTGACCAGCGAAAACTGGCACTACGACCCGGATTATAACTTCAGCATTCGTCACAAAACCCCGATCTTTAGCTACACCTATGCGCTGACCTTCTTTGACTTCACCGATGTTATTCCGATGTTTCGTGACGTGACCGTTTACCACGGTTATAACGCGCACAACCTGGACCCGCAGACCCGTGTGCACTACGTTAGCAAGGATATCTATGAGCTGGGTGTGGTTGAAGTGAAGACCAACTGCGGCAACAAAGTTCGTGCGTACAACATCGAGCGTACCATTTGCGACTTCATCAAGCACCGTGATAAAATTGAGGCGGAACTGTTTGCGAAGACCATGTACAAATATAGCGAGTACGAAAAACGTGATTGGAAGAAACTGCACGAGTATGCGAAGAAAATGAACATCGGCAAGAAAGTGTACGAAGTTTTCCAAGTGCTGGTTTAAGGATCCAGACTT

**Sequences of TA elements cloned into pMYCO1**

**NATURAL PROMOTER (Capital letters, bold)**

**Spiralin promoter (Small letters, bold)**

pMYCO1 backbone bordering sequences (in yellow)

pMYCO1 with natural promotor and A_132_

CGGGTACCGAGCTCGAATTC**TTAATATAATTAATTTAAAAGTTGCTTTTTATAATAAGGGCAACTTTTTTTAATATTAAAACTATAAAAATAAATTTTTTATTTTTTGTCCTATTTAATTGCTTTTTGTCCATTGTGAAATATAATAACTTTGAAAAGAGTTAAAATC**ATGAAAAAGGCAAATGTTTTAAATTTAATTAGATATCACATAGAAGAAAATGACATATCTTTTAGAAAAGAAGCACGTATTATAGCTGAAGAATTTTACAAAATGGGTGATGATGAGTTAGCTGAATATGTTTTATTTATGTTAAGAGATGCAAATCATTTTGTTCCACAAATAGATCAAGAGTATGATATTCAAATACCGTTTACTCAAAAAATAGAACTTGAAAGAAATAGTGAGCCTTTACCACTTCCACAAGTTATAAGTGAAGAAATAAAAGGAGTTATTAATGCTATTAGTAAAAATAGAAAGATAAATAAGTTTTTATTTCAAGGTTTTCCTGGAACTGGAAAAACTGAAACTGTAAAACAAATAGCAAGAATATTAAATAGAAATCTTTTTATGGTTGATTTTAATAATTTAATAGATTCTCATTTAGGTCAATCAAGTAAAAATATCGCTGAATTATTTCAAAAAATTAATCAAACTCCAAATCCAAAAAAAATTATTATCTGTTTTGATGAAATTGATGCTTTAGCTTTAGATAGAACAAACAAAACTGATCTAAGAGAAATGGGTAGAGTAACAACTGCTGTTTTTCAAGGGTTAGATAAACTAGATACTGATATTATTGTTTTTGCAACAACAAATCTCTTTAAACATTTTGATAAAGCTTTAATTAGAAGATTTGACCTTGTAATTGATTTTAATAGATACACAAAAAAAGATATGTTAGATATAGCTGAAATTATATTAAAGCATTATATTAAAAAAGTAGATAACATTAAAAGTGAATTAAGATTATTTAGAAAAATTATTTCATTATCAGAAGAATTGATTTATCCTGGTGATTTAAAAAATATTATCAAATCAAGTATTTATTTAAGTGATTATGAAGATCAATATGATTATTTAAAAAGGATTTATAAAAAGATAACTGATGATAAGCTTGACATTAGACAACTTAATGAAAACAATTTTACTGTACGAGAAATTGAAATTTTAAAAGGTTTATCAAAAAGTAGCGTAGCTCTGAAAGTGAAGGAGTTAAATTCAAATGAATAAGCCCTATAGTGAGTCGTATT

pMYCO1 with natural promotor and T_133_

CGGGTACCGAGCTCGAATTC**TTAATATAATTAATTTAAAAGTTGCTTTTTATAATAAGGGCAACTTTTTTTAATATTAAAACTATAAAAATAAATTTTTTATTTTTTGTCCTATTTAATTGCTTTTTGTCCATTGTGAAATATAATAACTTTGAAAAGAGTTAAAATC**ATGAATAATCTAATTGTATTAAAAGGAAAGTTTGAACCAGGTAAAAATACTAAGAAACCTAATAGTCCTCAAATACCAAAAACATCTATTATAAAATTAGAAGATTGTTATAGAATTCTTGATCAATTAATAAAAGCATCTAGTTTTTGAAAAGAACAAAAAATAGATATTAATCCAATAATAAATGTTAAATATAAAAGAATTATTTCTAAAAGTAACAGAGTAAGTTATTTGTTGTTAAAAAGTTTACAAAAAAATAACGAGCATATTATAGGTTCAAGTTTTTTGGATGAATTAGTTGAAAAAAAAATAGTGAAAAAGCAAGTCATCACTTATTGTCTGACACAAAAAGATTTACAAGAAGCAATAAAAAGATTAGATACAATAACTAATATTTTAAAAAAAACTCATTTTAAACGAATTGATAATAACTTAATTAACCTAATAGCTAATGAACAATATTTACCTATAAAAAAAGAAATTCAAAAATATGAATTTCTTAGTAGAACAGCTTTTATATCAACTTTGGTTGACCTTAATTACATAGAAGAAATTTTTATAAAAACTACTCATATTGATAATAATGTTGATAGTGTAGTCACTTTATATGATACAGGTATAAAAGCAATTGATTTGTTAAATAAACTTGATATTAATGTTAATATGTCAGATTTTATTGATGATTACACCTTATTTTTAGATAGAAATCAGTATAATGAATTAAAAACTAAGGCACCGTTTTTAATATCTATGTCAGTTGATGATCTTACTAAATTCATAATAGATGATAAACAAGAAGAAATTACAAAAAATGATATTATTTCTATACCAGATCCAACTAATGAACCAATAGTTGGCGTTATTGATACAATGTTTTGTAAAGATGTTTATTTTTCTAAATGAGTAGATTTTAGAAAAGAGGTTTCTGATGATATTTTATTAGATTCTAAAGATTATCAACATGGAACTCAAGTTAGTTCTATTATTGTTGATGGTCCTTCTTTTAATAAAAAATTAGAAGATGGATGTGGAAGGTTTAGAGTTAGACATTTTGGTGTTATGGCACATTCTAGTGGCAATGTCTTTTCTTTATTTAAAAAAATAAAATCGATAGTTATTAATAACTTAGATATAAAAGTTTGAAATTTATCATTAGGTTCAATTCGTGAAGTTAGTTCTAATTATATCTCATTACTAGGTTCTTTACTTGATCAATTACAATATGAAAATGACGTTATTTTTATTGTTGCAGGAACTAATGATAACGAGTGTAAACAAAAAATAGTAGGTTCACCTGCTGATTCAATAAACTCAATTGTTGTTAATTCAGTAGATTTTAAAAATAAACCAGCAAATTACTCTCGTAAAGGTCCTGTTTTAACATATTTTAATAAACCTGATATTAGTTATTATGGTGGAGTTGATAATAATAAAATTACTGTATGTGGCTGTTATGGTGAAGCAAAAGTTCAAGGTACTTCATTTGCAGCCCCATGAATTACTAGAAAAGTAGCTTATTTAATTTATAAAATGAATTATAGTAAAGAAGAAGCAAAAGCGTTAATAATTGATTCAGCAATTAAATTTGATAAACAAAAAGATAATAATAGAGATCTTATTGGATATGGTGTAGTTCCTATACATATTAATGAAATACTACAATCAAAAAATACTGATATAAAAGTTTTATTATCATATAATACAAAAGCTTATTATACATATAATTTCAACTTACCAGTTCCAACTAAAGAAAATAAATTTCCCTTTATTGCAAAACTTACTTTTGCTTATTTTGCAGAATCACAAAGAAGTCAAGGTGTTGACTATACACAAGATGAATTAGATATTCAATTTGGACCAATAGATAACAAATCTGAATCTATTAATGATATAAATGAAAATAATCAATCTAGTTCTAGTTCAAATGCTTATATTTATGAATATGAAGCTAGAAAAATGTTTGCAAAATGAAATACTGTTAAATCTATAATCAAATGATCTAAAACTAATAAAGGTAAAAAAAGACAATTTATAAAAACAACAAATAACAGATGAGGTATTAGAGTAATTAGAAAAACTAGAACTGATAATATAAATAATAAATCAATAAAGTTTAGTCTAGTTATAACTTTTAGATCCATTGATAATAAAGATAGAATTGAAGAGTTTATTAGTTTGTGTAACAAAAGTGGTTATTGAGTTGCAAGTAAAGTTCAAATTGATAATAAAATTGATATTCATGGAAAGTCAAATGAATATTTAGATTTTGAATAGGCCCTATAGTGAGTCGTATT

pMYCO1 with spiralin promotor and T_133_

CGGGTACCGAGCTCGAATTC**gaattaaaagttagtgaacaagaaaacagtgaagcaccagtttctgaaccaaaagaagacgaaaaaacaaaaaaagattaagcaatttatttggaaaatctttttttgtttttttaagaaatatttattgtttttttttaaaaattattgtacaattgctactataagggaaagaaaaaaagaaagatataaattgtataaagtagggttagaagcaattaataattattattaatgttatttttctcttatatattcaatgtaattttaattacatttgcttttaataaaaacactacttaatagagaaaggaaatataagaa**ATGAATAATCTAATTGTATTAAAAGGAAAGTTTGAACCAGGTAAAAATACTAAGAAACCTAATAGTCCTCAAATACCAAAAACATCTATTATAAAATTAGAAGATTGTTATAGAATTCTTGATCAATTAATAAAAGCATCTAGTTTTTGAAAAGAACAAAAAATAGATATTAATCCAATAATAAATGTTAAATATAAAAGAATTATTTCTAAAAGTAACAGAGTAAGTTATTTGTTGTTAAAAAGTTTACAAAAAAATAACGAGCATATTATAGGTTCAAGTTTTTTGGATGAATTAGTTGAAAAAAAAATAGTGAAAAAGCAAGTCATCACTTATTGTCTGACACAAAAAGATTTACAAGAAGCAATAAAAAGATTAGATACAATAACTAATATTTTAAAAAAAACTCATTTTAAACGAATTGATAATAACTTAATTAACCTAATAGCTAATGAACAATATTTACCTATAAAAAAAGAAATTCAAAAATATGAATTTCTTAGTAGAACAGCTTTTATATCAACTTTGGTTGACCTTAATTACATAGAAGAAATTTTTATAAAAACTACTCATATTGATAATAATGTTGATAGTGTAGTCACTTTATATGATACAGGTATAAAAGCAATTGATTTGTTAAATAAACTTGATATTAATGTTAATATGTCAGATTTTATTGATGATTACACCTTATTTTTAGATAGAAATCAGTATAATGAATTAAAAACTAAGGCACCGTTTTTAATATCTATGTCAGTTGATGATCTTACTAAATTCATAATAGATGATAAACAAGAAGAAATTACAAAAAATGATATTATTTCTATACCAGATCCAACTAATGAACCAATAGTTGGCGTTATTGATACAATGTTTTGTAAAGATGTTTATTTTTCTAAATGAGTAGATTTTAGAAAAGAGGTTTCTGATGATATTTTATTAGATTCTAAAGATTATCAACATGGAACTCAAGTTAGTTCTATTATTGTTGATGGTCCTTCTTTTAATAAAAAATTAGAAGATGGATGTGGAAGGTTTAGAGTTAGACATTTTGGTGTTATGGCACATTCTAGTGGCAATGTCTTTTCTTTATTTAAAAAAATAAAATCGATAGTTATTAATAACTTAGATATAAAAGTTTGAAATTTATCATTAGGTTCAATTCGTGAAGTTAGTTCTAATTATATCTCATTACTAGGTTCTTTACTTGATCAATTACAATATGAAAATGACGTTATTTTTATTGTTGCAGGAACTAATGATAACGAGTGTAAACAAAAAATAGTAGGTTCACCTGCTGATTCAATAAACTCAATTGTTGTTAATTCAGTAGATTTTAAAAATAAACCAGCAAATTACTCTCGTAAAGGTCCTGTTTTAACATATTTTAATAAACCTGATATTAGTTATTATGGTGGAGTTGATAATAATAAAATTACTGTATGTGGCTGTTATGGTGAAGCAAAAGTTCAAGGTACTTCATTTGCAGCCCCATGAATTACTAGAAAAGTAGCTTATTTAATTTATAAAATGAATTATAGTAAAGAAGAAGCAAAAGCGTTAATAATTGATTCAGCAATTAAATTTGATAAACAAAAAGATAATAATAGAGATCTTATTGGATATGGTGTAGTTCCTATACATATTAATGAAATACTACAATCAAAAAATACTGATATAAAAGTTTTATTATCATATAATACAAAAGCTTATTATACATATAATTTCAACTTACCAGTTCCAACTAAAGAAAATAAATTTCCCTTTATTGCAAAACTTACTTTTGCTTATTTTGCAGAATCACAAAGAAGTCAAGGTGTTGACTATACACAAGATGAATTAGATATTCAATTTGGACCAATAGATAACAAATCTGAATCTATTAATGATATAAATGAAAATAATCAATCTAGTTCTAGTTCAAATGCTTATATTTATGAATATGAAGCTAGAAAAATGTTTGCAAAATGAAATACTGTTAAATCTATAATCAAATGATCTAAAACTAATAAAGGTAAAAAAAGACAATTTATAAAAACAACAAATAACAGATGAGGTATTAGAGTAATTAGAAAAACTAGAACTGATAATATAAATAATAAATCAATAAAGTTTAGTCTAGTTATAACTTTTAGATCCATTGATAATAAAGATAGAATTGAAGAGTTTATTAGTTTGTGTAACAAAAGTGGTTATTGAGTTGCAAGTAAAGTTCAAATTGATAATAAAATTGATATTCATGGAAAGTCAAATGAATATTTAGATTTTGAATAGGCCCTATAGTGAGTCGTATT

pMYCO1 with natural promotor and TA_133/2_

CGGGTACCGAGCTCGAATTC**TTAATATAATTAATTTAAAAGTTGCTTTTTATAATAAGGGCAACTTTTTTTAATATTAAAACTATAAAAATAAATTTTTTATTTTTTGTCCTATTTAATTGCTTTTTGTCCATTGTGAAATATAATAACTTTGAAAAGAGTTAAAATC**ATGAAAAAGGCAAATGTTTTAAATTTAATTAGATATCACATAGAAGAAAATGACATATCTTTTAGAAAAGAAGCACGTATTATAGCTGAAGAATTTTACAAAATGGGTGATGATGAGTTAGCTGAATATGTTTTATTTATGTTAAGAGATGCAAATCATTTTGTTCCACAAATAGATCAAGAGTATGATATTCAAATACCGTTTACTCAAAAAATAGAACTTGAAAGAAATAGTGAGCCTTTACCACTTCCACAAGTTATAAGTGAAGAAATAAAAGGAGTTATTAATGCTATTAGTAAAAATAGAAAGATAAATAAGTTTTTATTTCAAGGTTTTCCTGGAACTGGAAAAACTGAAACTGTAAAACAAATAGCAAGAATATTAAATAGAAATCTTTTTATGGTTGATTTTAATAATTTAATAGATTCTCATTTAGGTCAATCAAGTAAAAATATCGCTGAATTATTTCAAAAAATTAATCAAACTCCAAATCCAAAAAAAATTATTATCTGTTTTGATGAAATTGATGCTTTAGCTTTAGATAGAACAAACAAAACTGATCTAAGAGAAATGGGTAGAGTAACAACTGCTGTTTTTCAAGGGTTAGATAAACTAGATACTGATATTATTGTTTTTGCAACAACAAATCTCTTTAAACATTTTGATAAAGCTTTAATTAGAAGATTTGACCTTGTAATTGATTTTAATAGATACACAAAAAAAGATATGTTAGATATAGCTGAAATTATATTAAAGCATTATATTAAAAAAGTAGATAACATTAAAAGTGAATTAAGATTATTTAGAAAAATTATTTCATTATCAGAAGAATTGATTTATCCTGGTGATTTAAAAAATATTATCAAATCAAGTATTTATTTAAGTGATTATGAAGATCAATATGATTATTTAAAAAGGATTTATAAAAAGATAACTGATGATAAGCTTGACATTAGACAACTTAATGAAAACAATTTTACTGTACGAGAAATTGAAATTTTAAAAGGTTTATCAAAAAGTAGCGTAGCTCTGAAAGTGAAGGAGTTAAATTCAAATGAATAATCTAATTGTATTAAAAGGAAAGTTTGAACCAGGTAAAAATACTAAGAAACCTAATAGTCCTCAAATACCAAAAACATCTATTATAAAATTAGAAGATTGTTATAGAATTCTTGATCAATTAATAAAAGCATCTAGTTTTTGAAAAGAACAAAAAATAGATATTAATCCAATAATAAATGTTAAATATAAAAGAATTATTTCTAAAAGTAACAGAGTAAGTTATTTGTTGTTAAAAAGTTTACAAAAAAATAACGAGCATATTATAGGTTCAAGTTTTTTGGATGAATTAGTTGAAAAAAAAATAGTGAAAAAGCAAGTCATCACTTATTGTCTGACACAAAAAGATTTACAAGAAGCAATAAAAAGATTAGATACAATAACTAATATTTTAAAAAAAACTCATTTTAAACGAATTGATAATAACTTAATTAACCTAATAGCTAATGAACAATATTTACCTATAAAAAAAGAAATTCAAAAATATGAATTTCTTAGTAGAACAGCTTTTATATCAACTTTGGTTGACCTTAATTACATAGAAGAAATTTTTATAAAAACTACTCATATTGATAATAATGTTGATAGTGTAGTCACTTTATATGATACAGGTATAAAAGCAATTGATTTGTTAAATAAACTTGATATTAATGTTAATATGTCAGATTTTATTGATGATTACACCTTATTTTTAGATAGAAATCAGTATAATGAATTAAAAACTAAGGCACCGTTTTTAATATCTATGTCAGTTGATGATCTTACTAAATTCATAATAGATGATAAACAAGAAGAAATTACAAAAAATGATATTATTTCTATACCAGATCCAACTAATGAACCAATAGTTGGCGTTATTGATACAATGTTTTGTAAAGATGTTTATTTTTCTAAATGAGTAGATTTTAGAAAAGAGGTTTCTGATGATATTTTATTAGATTCTAAAGATTATCAACATGGAACTCAAGTTAGTTCTATTATTGTTGATGGTCCTTCTTTTAATAAAAAATTAGAAGATGGATGTGGAAGGTTTAGAGTTAGACATTTTGGTGTTATGGCACATTCTAGTGGCAATGTCTTTTCTTTATTTAAAAAAATAAAATCGATAGTTATTAATAACTTAGATATAAAAGTTTGAAATTTATCATTAGGTTCAATTCGTGAAGTTAGTTCTAATTATATCTCATTACTAGGTTCTTTACTTGATCAATTACAATATGAAAATGACGTTATTTTTATTGTTGCAGGAACTAATGATAACGAGTGTAAACAAAAAATAGTAGGTTCACCTGCTGATTCAATAAACTCAATTGTTGTTAATTCAGTAGATTTTAAAAATAAACCAGCAAATTACTCTCGTAAAGGTCCTGTTTTAACATATTTTAATAAACCTGATATTAGTTATTATGGTGGAGTTGATAATAATAAAATTACTGTATGTGGCTGTTATGGTGAAGCAAAAGTTCAAGGTACTTCATTTGCAGCCCCATGAATTACTAGAAAAGTAGCTTATTTAATTTATAAAATGAATTATAGTAAAGAAGAAGCAAAAGCGTTAATAATTGATTCAGCAATTAAATTTGATAAACAAAAAGATAATAATAGAGATCTTATTGGATATGGTGTAGTTCCTATACATATTAATGAAATACTACAATCAAAAAATACTGATATAAAAGTTTTATTATCATATAATACAAAAGCTTATTATACATATAATTTCAACTTACCAGTTCCAACTAAAGAAAATAAATTTCCCTTTATTGCAAAACTTACTTTTGCTTATTTTGCAGAATCACAAAGAAGTCAAGGTGTTGACTATACACAAGATGAATTAGATATTCAATTTGGACCAATAGATAACAAATCTGAATCTATTAATGATATAAATGAAAATAATCAATCTAGTTCTAGTTCAAATGCTTATATTTATGAATATGAAGCTAGAAAAATGTTTGCAAAATGAAATACTGTTAAATCTATAATCAAATGATCTAAAACTAATAAAGGTAAAAAAAGACAATTTATAAAAACAACAAATAACAGATGAGGTATTAGAGTAATTAGAAAAACTAGAACTGATAATATAAATAATAAATCAATAAAGTTTAGTCTAGTTATAACTTTTAGATCCATTGATAATAAAGATAGAATTGAAGAGTTTATTAGTTTGTGTAACAAAAGTGGTTATTGAGTTGCAAGTAAAGTTCAAATTGATAATAAAATTGATATTCATGGAAAGTCAAATGAATATTTAGATTTTGAATAGGCCCTATAGTGAGTCGTATT

pMYCO1 with natural promotor and A_753_

CGGGTACCGAGCTCGAATTC**ACTAGGTATTTTTAAAGTTCTTAGAGATTTTTAAAATACTTAGAATTCTTTAATACTAGTTAGTATTTATAAAAAGTAATAATTAATAATAGTTTTGATATTAAAAATTTAAAATCATTTTAAATTAAATGATATTTCTTGTGTTTACTTTTAGGGTATCAGTCCCATGTGTATAATCTAAATGCATAAATAAAACTAGGAGTAAGA**ATGAAAAAAGTAAATGTCAATATTAAAATGGATCCTGAAATTAAAAAACAGGCTAGTTTATTATTTAAAGAATTTGGTATGACCATGTCTTCTGCTATTAATCTATTTGTGAAAACTGCTGTTGAACAAAACGACATTCCATTTGAATATAATAGAGAGATAACTAACAAAGAAACTTTAGAAGCCTTTAAAGAAGGTGAACGTCTTTTAGCTGATAAAAACGCAAAACGTTACTCAAGTTTTAAAGAAATATTAGATTCATTAGATAAATAAGCCCTATAGTGAGTCGTATT

pMYCO1 with natural promotor and T_752_

CGGGTACCGAGCTCGAATTC**ATAACCATCCTTTTTCTATGTTTTACTTTCAAATAATTTGTATTCTATTTAATTTTATTTTATTTATAAATTAGAAAAGGGATTTTGGCACTATTTTTAAAAAAACAATATATTATAAAGCAATTATTCTCATTTGTATATACTATTTTTGACGAAATTTAAAAAAATTCTTTAAATAAGCTAAAAATACGTGGTTTTTTTAAATAAACCCTGTATTTTCTACACTTTTTGTATTTTTTTATAATTTAAAATAAAAATGATTTTTAGACTTAATTTCATAAGATTTTCTATTAATATTTAACTTTTTATAAACTTAATAAAATATATAATTAATTCATAAATAAATAAGTTAATAGTAATAGTAAATAT**ATGAAAGCTAATTTTTTAGAAGAAGAATTTGAGATTAATTTATCTGTTAAACACTTATTAGAGTTATGAGATAAAAATTTATTAAATACATTTGAAATAGGTACTTTTAAAGGTTTAAGTCAAATACATTCTTATATGTTTAAAGATATATTTGACTTTAATGGACAAATTAGAAATGTTAATATCTCTAAAAATAACTTATGTTTTGTTTAGCAAGATATTTAAAACAAAACTTAGAAATTATTGATAATATGAAACATGATACATTTGATCAGATCATAGATAAATATGTTGAAATGAATATTTGTCATCCATTTAGAGAAGGTAATGGCAGATCTATGAGAATTTGATTAGATTTAATATTAAAAAAACAACTAAATGTAGTTGTTAATTGAACTAATATAAATAAAGATGAGTACTTATTAGCAATGATTAATTCTTTAATTGATTCAACTAATCTTAAATTACTAATTAAAAACAACTTAACAAATAAGATTACTGATAGAAATGTATATATTAAAAGTATTATTAAATCTTATGAGTATGAAGGTTTTAAAATAAATATTAAATAAGCCCTATAGTGAGTCGTATT

pMYCO1 with spiralin promotor and T_752_

CGGGTACCGAGCTCGAATTC**gaattaaaagttagtgaacaagaaaacagtgaagcaccagtttctgaaccaaaagaagacgaaaaaacaaaaaaagattaagcaatttatttggaaaatctttttttgtttttttaagaaatatttattgtttttttttaaaaattattgtacaattgctactataagggaaagaaaaaaagaaagatataaattgtataaagtagggttagaagcaattaataattattattaatgttatttttctcttatatattcaatgtaattttaattacatttgcttttaataaaaacactacttaatagagaaaggaaatataagaa**ATGAAAGCTAATTTTTTAGAAGAAGAATTTGAGATTAATTTATCTGTTAAACACTTATTAGAGTTATGAGATAAAAATTTATTAAATACATTTGAAATAGGTACTTTTAAAGGTTTAAGTCAAATACATTCTTATATGTTTAAAGATATATTTGACTTTAATGGACAAATTAGAAATGTTAATATCTCTAAAAATAACTTATGTTTTGTTTAGCAAGATATTTAAAACAAAACTTAGAAATTATTGATAATATGAAACATGATACATTTGATCAGATCATAGATAAATATGTTGAAATGAATATTTGTCATCCATTTAGAGAAGGTAATGGCAGATCTATGAGAATTTGATTAGATTTAATATTAAAAAAACAACTAAATGTAGTTGTTAATTGAACTAATATAAATAAAGATGAGTACTTATTAGCAATGATTAATTCTTTAATTGATTCAACTAATCTTAAATTACTAATTAAAAACAACTTAACAAATAAGATTACTGATAGAAATGTATATATTAAAAGTATTATTAAATCTTATGAGTATGAAGGTTTTAAAATAAATATTAAATAAGCCCTATAGTGAGTCGTATT

pMYCO1 with natural promotor and TA_752/3_

CGGGTACCGAGCTCGAATTC**ATAACCATCCTTTTTCTATGTTTTACTTTCAAATAATTTGTATTCTATTTAATTTTATTTTATTTATAAATTAGAAAAGGGATTTTGGCACTATTTTTAAAAAAACAATATATTATAAAGCAATTATTCTCATTTGTATATACTATTTTTGACGAAATTTAAAAAAATTCTTTAAATAAGCTAAAAATACGTGGTTTTTTTAAATAAACCCTGTATTTTCTACACTTTTTGTATTTTTTTATAATTTAAAATAAAAATGATTTTTAGACTTAATTTCATAAGATTTTCTATTAATATTTAACTTTTTATAAACTTAATAAAATATATAATTAATTCATAAATAAATAAGTTAATAGTAATAGTAAATAT**ATGAAAGCTAATTTTTTAGAAGAAGAATTTGAGATTAATTTATCTGTTAAACACTTATTAGAGTTATGAGATAAAAATTTATTAAATACATTTGAAATAGGTACTTTTAAAGGTTTAAGTCAAATACATTCTTATATGTTTAAAGATATATTTGACTTTAATGGACAAATTAGAAATGTTAATATCTCTAAAAATAACTTATGTTTTGTTTAGCAAGATATTTAAAACAAAACTTAGAAATTATTGATAATATGAAACATGATACATTTGATCAGATCATAGATAAATATGTTGAAATGAATATTTGTCATCCATTTAGAGAAGGTAATGGCAGATCTATGAGAATTTGATTAGATTTAATATTAAAAAAACAACTAAATGTAGTTGTTAATTGAACTAATATAAATAAAGATGAGTACTTATTAGCAATGATTAATTCTTTAATTGATTCAACTAATCTTAAATTACTAATTAAAAACAACTTAACAAATAAGATTACTGATAGAAATGTATATATTAAAAGTATTATTAAATCTTATGAGTATGAAGGTTTTAAAATAAATATTAAATAA**ACTAGGTATTTTTAAAGTTCTTAGAGATTTTTAAAATACTTAGAATTCTTTAATACTAGTTAGTATTTATAAAAAGTAATAATTAATAATAGTTTTGATATTAAAAATTTAAAATCATTTTAAATTAAATGATATTTCTTGTGTTTACTTTTAGGGTATCAGTCCCATGTGTATAATCTAAATGCATAAATAAAACTAGGAGTAAGA**ATGAAAAAAGTAAATGTCAATATTAAAATGGATCCTGAAATTAAAAAACAGGCTAGTTTATTATTTAAAGAATTTGGTATGACCATGTCTTCTGCTATTAATCTATTTGTGAAAACTGCTGTTGAACAAAACGACATTCCATTTGAATATAATAGAGAGATAACTAACAAAGAAACTTTAGAAGCCTTTAAAGAAGGTGAACGTCTTTTAGCTGATAAAAACGCAAAACGTTACTCAAGTTTTAAAGAAATATTAGATTCATTAGATAAATAAGCCCTATAGTGAGTCGTATT

pMYCO1 with natural promotor and A_161_

CGGGTACCGAGCTCGAATTC**AAAATATCTTAAATAACCATAAGTTAAAATTATTTGACTTTTGGTTATTTTTTTATTATTTTTTTCTAATACTATAATGAAAAGTTTAAATACTTTTTAATAAATAAAATAAAAAATTATACAAATTTACTTAATGCTAATTAACTTGTTTGTTTCTTATTTGCTTAAAAATGATAAAATTTAAGAAAAATAAGAACATAGGTGATAATT**ATGAATTATAAAGAACAACTGATAAAAGAAGCCAAAAAAAGAGACGGTCTTATTACAAGAAAAGAAATTATAAATTTAAAAATTCCATCAATTTATATTACAAGAATGATTAGAAGTAATGAATTAGAAAAAGTCGATATTGGTATTTATGCTCTAACTTCTGAAAATTGGCACTATGATCCTGATTATAATTTTAGTATCAGACATAAAACACCTATTTTTTCTTATACTTATGCACTTACATTTTTTGATTTTACAGATGTTATTCCAATGTTTCGTGATGTAACTGTTTATCATGGATATAATGCTCATAATTTAGATCCACAAACTAGAGTACACTATGTTTCAAAAGATATATATGAACTTGGAGTTGTAGAAGTAAAAACAAATTGTGGTAATAAAGTTAGAGCTTATAATATTGAAAGAACAATTTGTGACTTTATTAAACATAGAGATAAAATCGAAGCTGAACTATTTGCAAAAACTATGTATAAATATTCTGAATATGAAAAAAGAGATTGAAAAAAACTTCATGAATATGCTAAAAAAATGAACATTGGTAAGAAAGTTTATGAAGTTTTTCAAGTATTAGTTTAAGCCCTATAGTGAGTCGTATT

pMYCO1 with natural promotor and T_160_

CGGGTACCGAGCTCGAATTC**AAAATATCTTAAATAACCATAAGTTAAAATTATTTGACTTTTGGTTATTTTTTTATTATTTTTTTCTAATACTATAATGAAAAGTTTAAATACTTTTTAATAAATAAAATAAAAAATTATACAAATTTACTTAATGCTAATTAACTTGTTTGTTTCTTATTTGCTTAAAAATGATAAAATTTAAGAAAAATAAGAACATAGGTGATAATT**ATGATATCCATATTAGAAAAAATTGTTAAAAGTAAAAATAAGCAAAACTTTATAATTAAAGGCGGATTCTTACTTTCTAATATGATGAGTTTAAACGAAAGAACCACTCAAGATATAGATTGCCTAATAAAGGGTATCAATTTTGTTAAAGAGAATATTCTAAAAATTATTTCTGATGTACTAAGATATGATTTTACTGACTTTATAGAATATGAAATAAAAGAAATAACAGAAATAAAAAAGAGAGACAAATATAATGGATTTAGAGTCAAAATATTATGTAAATTAGAAAAGTTAGAAGAAATAGTAAAAATAGATTTAGCAATTGGTGATGTTATAACACCATCTGAGATTAAATACAATTTTAAAAATATCTTTTCAAATAGTAGCTTTGAAATACAAGCATATAATTTAGAAACGATATTAGCTGAAAAAATATTTATAATTAATAATTTAAATATACAATCAACTAGAATAAAAGATCTTTATGATATTTTTTTAATATATAGTTTGAAAGAGTTTGAGATTGATTATAATATTCTAAAATGTGCTTGTTTAAATACTTTTAAAAAAAGAAATACTGTTTTTAATATAGATTTAATACTTAATTTATTAAATGAAATTAACCAATTAGATATTTTTAAATTACTTTGAAATAATTACAAGCAAAAATACTTTTACGCTCAAAAAATAGAATTTGAAATACTGGTGGAATCTGTAATTAAGCTTTTAGAAAAGTTAAAAAGCTCTTAGGCCCTATAGTGAGTCGTATT

pMYCO1 with spiralin promotor and T_160_

CGGGTACCGAGCTCGAATTC**gaattaaaagttagtgaacaagaaaacagtgaagcaccagtttctgaaccaaaagaagacgaaaaaacaaaaaaagattaagcaatttatttggaaaatctttttttgtttttttaagaaatatttattgtttttttttaaaaattattgtacaattgctactataagggaaagaaaaaaagaaagatataaattgtataaagtagggttagaagcaattaataattattattaatgttatttttctcttatatattcaatgtaattttaattacatttgcttttaataaaaacactacttaatagagaaaggaaatataagaa**ATGATATCCATATTAGAAAAAATTGTTAAAAGTAAAAATAAGCAAAACTTTATAATTAAAGGCGGATTCTTACTTTCTAATATGATGAGTTTAAACGAAAGAACCACTCAAGATATAGATTGCCTAATAAAGGGTATCAATTTTGTTAAAGAGAATATTCTAAAAATTATTTCTGATGTACTAAGATATGATTTTACTGACTTTATAGAATATGAAATAAAAGAAATAACAGAAATAAAAAAGAGAGACAAATATAATGGATTTAGAGTCAAAATATTATGTAAATTAGAAAAGTTAGAAGAAATAGTAAAAATAGATTTAGCAATTGGTGATGTTATAACACCATCTGAGATTAAATACAATTTTAAAAATATCTTTTCAAATAGTAGCTTTGAAATACAAGCATATAATTTAGAAACGATATTAGCTGAAAAAATATTTATAATTAATAATTTAAATATACAATCAACTAGAATAAAAGATCTTTATGATATTTTTTTAATATATAGTTTGAAAGAGTTTGAGATTGATTATAATATTCTAAAATGTGCTTGTTTAAATACTTTTAAAAAAAGAAATACTGTTTTTAATATAGATTTAATACTTAATTTATTAAATGAAATTAACCAATTAGATATTTTTAAATTACTTTGAAATAATTACAAGCAAAAATACTTTTACGCTCAAAAAATAGAATTTGAAATACTGGTGGAATCTGTAATTAAGCTTTTAGAAAAGTTAAAAAGCTCTTAGGCCCTATAGTGAGTCGTATT

pMYCO1 with natural promotor and TA_160/1_

CGGGTACCGAGCTCGAATTC**AAAATATCTTAAATAACCATAAGTTAAAATTATTTGACTTTTGGTTATTTTTTTATTATTTTTTTCTAATACTATAATGAAAAGTTTAAATACTTTTTAATAAATAAAATAAAAAATTATACAAATTTACTTAATGCTAATTAACTTGTTTGTTTCTTATTTGCTTAAAAATGATAAAATTTAAGAAAAATAAGAACATAGGTGATAATT**ATGAATTATAAAGAACAACTGATAAAAGAAGCCAAAAAAAGAGACGGTCTTATTACAAGAAAAGAAATTATAAATTTAAAAATTCCATCAATTTATATTACAAGAATGATTAGAAGTAATGAATTAGAAAAAGTCGATATTGGTATTTATGCTCTAACTTCTGAAAATTGGCACTATGATCCTGATTATAATTTTAGTATCAGACATAAAACACCTATTTTTTCTTATACTTATGCACTTACATTTTTTGATTTTACAGATGTTATTCCAATGTTTCGTGATGTAACTGTTTATCATGGATATAATGCTCATAATTTAGATCCACAAACTAGAGTACACTATGTTTCAAAAGATATATATGAACTTGGAGTTGTAGAAGTAAAAACAAATTGTGGTAATAAAGTTAGAGCTTATAATATTGAAAGAACAATTTGTGACTTTATTAAACATAGAGATAAAATCGAAGCTGAACTATTTGCAAAAACTATGTATAAATATTCTGAATATGAAAAAAGAGATTGAAAAAAACTTCATGAATATGCTAAAAAAATGAACATTGGTAAGAAAGTTTATGAAGTTTTTCAAGTATTAGTTTAATGAACTTACAAAAATTAAAAGCAATTTGTAAAAGGTTATCAAATTAAACAAAAACAAATTATAATATCATCTTAAAACATTACTTTATGATATCCATATTAGAAAAAATTGTTAAAAGTAAAAATAAGCAAAACTTTATAATTAAAGGCGGATTCTTACTTTCTAATATGATGAGTTTAAACGAAAGAACCACTCAAGATATAGATTGCCTAATAAAGGGTATCAATTTTGTTAAAGAGAATATTCTAAAAATTATTTCTGATGTACTAAGATATGATTTTACTGACTTTATAGAATATGAAATAAAAGAAATAACAGAAATAAAAAAGAGAGACAAATATAATGGATTTAGAGTCAAAATATTATGTAAATTAGAAAAGTTAGAAGAAATAGTAAAAATAGATTTAGCAATTGGTGATGTTATAACACCATCTGAGATTAAATACAATTTTAAAAATATCTTTTCAAATAGTAGCTTTGAAATACAAGCATATAATTTAGAAACGATATTAGCTGAAAAAATATTTATAATTAATAATTTAAATATACAATCAACTAGAATAAAAGATCTTTATGATATTTTTTTAATATATAGTTTGAAAGAGTTTGAGATTGATTATAATATTCTAAAATGTGCTTGTTTAAATACTTTTAAAAAAAGAAATACTGTTTTTAATATAGATTTAATACTTAATTTATTAAATGAAATTAACCAATTAGATATTTTTAAATTACTTTGAAATAATTACAAGCAAAAATACTTTTACGCTCAAAAAATAGAATTTGAAATACTGGTGGAATCTGTAATTAAGCTTTTAGAAAAGTTAAAAAGCTCTTAGGCCCTATAGTGAGTCGTATT
